# Preclinical characterization and anti-SARS-CoV-2 efficacy of ATV014: an oral cyclohexanecarboxylate prodrug of 1′-CN-4-aza-7,9-dideazaadenosine C-nucleoside

**DOI:** 10.1038/s41392-023-01310-0

**Published:** 2023-01-12

**Authors:** Qifan Zhou, Sidi Yang, Liu Cao, Yang Yang, Tiefeng Xu, Qishu Chen, Hongzhou Lu, Yingjun Li, Deyin Guo, Xumu Zhang

**Affiliations:** 1 Shenzhen Key Laboratory of Small Molecule Drug Discovery and Synthesis, Department of Chemistry, College of Science, Academy for Advanced Interdisciplinary Studies and Medi-X Pingshan, Southern University of Science and Technology, Shenzhen, Guangdong 518000 China; 2 Centre for Infection and Immunity Studies (CIIS), School of Medicine, Shenzhen Campus of Sun Yat-sen University, Shenzhen, Guangdong 518107 China; 3 Guangzhou Laboratory, Bio-Island, Guangzhou, China; 4 Shenzhen Key Laboratory of Pathogen and Immunity, National Clinical Research Center for infectious disease, State Key Discipline of Infectious Disease, Shenzhen Third People′s Hospital, Second Hospital Affiliated to Southern University of Science and Technology, Shenzhen, China

**Keywords:** Drug development, Drug delivery, Medicinal chemistry

**Dear Editor**,

Severe acute respiratory syndrome coronavirus 2 (SARS-CoV-2), the causative agent of the global pandemic coronavirus disease 2019 (COVID-19), has proven itself to be a highly virulent respiratory pathogen with an unpredictable evolutionary capacity, posing a persistent threat to mankind. At the time of this manuscript’s publication, the dominant Omicron variant is characterized by significantly greater infectivity, and the emerging subvariants substantially display escaping neutralization induced by both vaccination and previous infection, raising the risk of vaccine breakthrough or reinfection.^1^ Therefore, oral directly-acting antiviral drugs are desperately needed as countermeasures to reduce viral transmission and the risk of disease progression to critical illness or death.

Remdesivir (RDV) is the first nucleotide analogue approved for treating COVID-19. Early administration of RDV to non-hospitalized high-risk patients could reduce the risk of hospitalization or death by 87%.^2^ However, as an obligatory intravenous drug, RDV is not readily accessible to COVID-19 outpatients. Previously, we found that the parent 1′-CN-4-aza-7,9-dideazaadenosine C-nucleoside (GS-441524) was more potent than RDV against SARS-CoV-2 in vitro.^3^ As a continuing effort to improve druggability and oral bioavailability of GS-441524,^4^ herein we report the preclinical characterization and anti-SARS-CoV-2 efficacy of a potential oral nucleoside drug candidate, ATV014.

Initially, we designed and synthesized a series of adenosine derivatives based on prodrug strategy by selectively introducing esters at 5′-OH group of GS-441524 to obtain 4a~4 m (Supplementary Fig. S1). Among these newly synthesized compounds, the cyclohexyl carboxylate analog 4a (ATV014) (Fig. 1a, EC_50_ = 0.48 μM) was identified as a potent agent against SARS-CoV-2 replicon, which was about 3.4-fold more potent than that of GS-441524 (EC_50_ = 1.644 μM) (Supplementary Table S1). ATV014, the tetrahydro-2H-pyran-4-carboxylate analog (4 h) and the palmitate analog (4 l) were chosen to compare the PK profile in SD rats (Supplementary Table S2). With a single oral dose of 25 mg/kg, ATV014 demonstrated an improvement in oral bioavailability (F%) of 53.4% and T_½_ of 1.9 h, superior than that of 4 h and 4 l. Accounting for the favorable oral PK and potency, ATV014 was selected for further anti-SARS-CoV-2 experiments with live viruses. As shown in Fig. 1b, in Vero E6 cells, the antiviral activities of ATV014 against B.1 (IC_50_ = 0.46 μM), Beta (IC_50_ = 0.13 μM), Delta (IC_50_ = 0.24 μM) and Omicron (IC_50_ = 0.013 μM) were significantly improved compared to RDV and GS-441524. The average cytotoxic concentration (CC_50_) values of ATV014 in Vero E6 cells were 263.8 μM. Notably, the therapeutic index (CC_50_/EC_50_) of ATV014 against Omicron variant reached 20292. Additionally, Against the B.1 strain in A549-ACE2 cells, ATV014 exhibited an EC50 value of 0.0562 ± 0.016 µM (Supplementary Fig. S2). These results demonstrated the high potency, especially against the recent prevalent Omicron, and relatively low toxicity of ATV014.

**Fig. 1:**
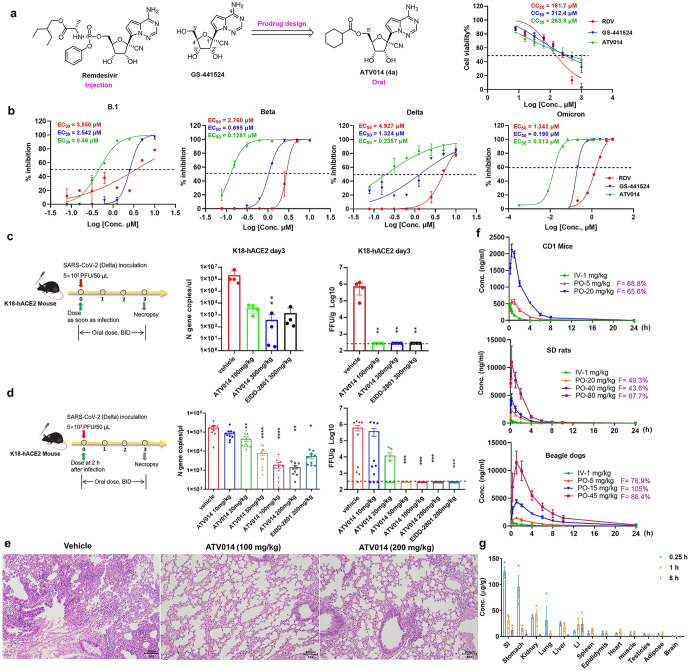
Definition of a potential orally anti-SARS-CoV-2 drug ATV014 and its preclinical characterization. **a** The chemical structures of remdesivir, GS-441524 and ATV014. **b** Antiviral activity of RDV, GS-441524, and ATV014 against B.1, Beta, Delta, and Omicron strains of SARS-CoV-2 and corresponding cytotoxicity in Vero E6 cells. **c** Schematic of the prophylactic efficacy in a K18-hACE2 mice model. K18-hACE2 mice were intranasally inoculated with SARS-CoV-2 Delta variant (5 × 10^2^ PFU virus per mouse) and were immediately oral treated with vehicle, ATV014 (100, 300 mg/kg) or EIDD-2801 (300 mg/kg), before the inoculation and continued for three days (bis in die (BID), *n* = 4 per group). Following results demonstrated the abundance of SARS-CoV-2 N gene copies in mouse lungs *via* qRT-PCR (quantitative real-time polymerase chain reaction) and virus viability via FFA (focus forming assay) at 3 dpi. The detection limit of qRT-PCR was 0.5 copies/μL. The red dashed line indicates the limit of detection for the FFA. **d** Flowsheet of the therapeutic efficacy. K18-hACE2 mice were intranasally inoculated with the SARS-CoV-2 Delta variant (5 × 10^2^ PFU per mouse) and were treated with vehicle, ATV014 (10, 20, 50, 100 or 200 mg/kg), or EIDD-2801 (200 mg/kg) at two hours after SARS-CoV-2 infection (*n* = 10 per group). Following results showed the abundance of SARS-CoV-2 N gene copies in mouse lungs *via* qRT-PCR and virus viability *via* FFA at 3 dpi. **e** Representative H&E images of lung sections of the lungs of the vehicle-treated, 100 mg/kg ATV014-treated and 200 mg/kg ATV014-treated mice. **f** Mean plasma concentrations of ATV014 following single intravenous (1.0 mg/kg) and oral (5 and 20 mg/kg) administration of ATV014 in CD1 mice (*n* = 3 per group). single intravenous (1.0 mg/kg) and oral (20, 40 and 80 mg/kg) administration of ATV014 in SD rats (*n* = 6 per group) and single intravenous (1 mg/kg) and oral (5, 15, and 45 mg/kg) administration in beagle dogs (*n* = 6 per group). **g** Tissue distribution of the key metabolite GS-441524 in male rats after a single oral administration of ATV014 at 80 mg/kg. SI: small intestine; LI: large intestine. Error bars indicate SEM. A Kruskal–Wallis test was used for statistical analysis. ^∗^*P* ≤ 0.05; ^∗∗^*P* ≤ 0.005; ^∗∗∗^*P* ≤ 0.0005; ^∗∗∗∗^*P* ≤ 0.0001

Subsequently, we evaluated the in vivo prophylactic and therapeutic efficacy of oral ATV014 in the Delta variant infected K18-hACE2 transgenic mice (Figs. 1c and 1d) with molnupiravir (EIDD-2801) as a reference compound.^5^ Treatment was initiated at the time of infection in the prophylactic model. At 3 days post inoculation (dpi), virus RNA copies in mice treated with ATV014 (100 and 300 mg/kg, BID) were significantly reduced. Virus titers in ATV014 treated mice as measured by FFA (focus forming assay) were reduced to near-detection limit, indicating that the infectious viruses in the lungs were significantly eliminated. To determine the lowest efficacious dose in the therapeutic setting, ATV014 was administrated 2 h after infection. 20, 50, 100, and 200 mg/kg of ATV014 dose-dependently reduced viral RNA in the lungs. ATV014 at 200 mg/kg significantly reduced viral RNA by approximately two orders of magnitude (1.4 × 10^3^ copies/μl compared to 1.7 × 10^5^ copies/μl in vehicle group), which was more potent relative to 200 mg/kg molnupiravir (5.4 × 10^3^ copies/μl). In focus forming assay, 50 mg/kg and higher dosage of ATV014 reduced concentration of infectious virus to below the limit of detection. Histopathological analysis revealed ATV014 protected lung tissue from interstitial inflammatory lesions and damage caused by SARS-CoV-2 Delta infection (Fig. 1e).

The kilo-scale up synthetic process of ATV014 has been optimized to ensure the supply for the clinical study. ATV014 was obtained as an anhydrate with high crystallinity and physically stable at 40 °C/75%RH (open) and 60 °C (open) for 30 days, displaying a good chemical stability. ATV014 had favorable experimental log P value of 1.99 and pKa value of 3.5. GS-441524 showed moderately low permeability with the Papp (A to B) value of 3.62 cm/s × 10^−6^ in Caco-2 cells permeability assays. In contrast, ATV014 behaved as a meso-hypertonic compound (Papp (A to B) = 8.69 cm/s × 10^−6^) and was a weak substrate efflux transporter with efflux rate of 2.5 (Supplementary Table S3). The improvement of passive absorptive permeability partially explained the improved oral absorption of ATV014. After a single oral administration of 20, 40 and 80 mg/kg of ATV014 to SD rats, the bioavailability (F) was 49%, 43%, and 68%, respectively (Fig. 1f and Supplementary Table S4). By comparison, the oral bioavailability of GS-441524 after administration of RDV was only 14% in rats (Supplementary Table S5). Meanwhile, the oral bioavailability of GS-441524 after administration of ATV014 ranged from 66 to 69% in mice and ranged from 78 to 105% in beagle dogs (Fig. 1f and Supplementary Table S6). C_max_ and AUC dose-dependently increased in all the three species. In the 7-day repeated administration of oral ATV014 in rats (40 mg/kg, BID) and dogs (15 mg/kg, BID), the AUC_last_ and C_max_ values on day 1 and day 7 were equivalent, indicating it did not accumulate in the body when dosing repeatedly (Supplementary Table S7 and S8).

Metabolism study showed that six metabolites of ATV014 were detected in mouse incubation samples, four in rat, dog and human incubation samples and three in monkey incubation samples (Supplementary Table S9 and Fig. S3). The hydrolyzed metabolite M3 (GS-441524) was the major metabolite in hepatocytes of all five species, with peak area percentages of 93.06%, 90.27%, 92.45%, 96.30%, and 95.64%, respectively. Further tissue distribution study showed that the primary metabolite GS-441524 widely and quickly distributed in rat tissues (Fig. 1g and Supplementary Table S10), especially in the lungs to be above the therapeutical concentrations. ATV014, GS-441524 and the active triphosphate form (RTP) demonstrated a favorable off-target selectivity profile in a broad panel of 87 targets screening. The inhibition rates were <50% at 20 μM against all the screened targets, except that **ATV014** inhibited 53% of adenosine transporter activity (Supplementary Table S11). For the single-dose toxicity studies, the maximum tolerated dose (MTD) of ATV014 was both 2000 mg/kg in rats and dogs. For 14 days of repeated dose toxicity studies, the no observed adverse effect levels (NOAELs) of ATV014 were 400 mg/kg and 100 mg/kg, and the MTD was 800 mg/kg and 300 mg/kg in rats and dogs, respectively. ATV014 and GS-441524 exhibited no mutagenicity or clastogenicity regarding the negative results in the Ames test using *Salmonella, E. coli* and micronucleus test in bone marrow cells. These anti-SARS-CoV-2 efficacy studies and preclinical data validated ATV014 as a potent oral antiviral for treating COVID-19 with favorable PK properties and safety profile, which is being investigated in clinical trials (Phase 1, NCT05504746; investigator-initiated trial, ChiCTR2200064093).

## Supplementary information

Preclinical characterization and anti-SARS-CoV-2 efficacy of ATV014: Oral cyclohexanecarboxylate prodrug of 1′-CN-4-aza-7,9-dideazaadenosine C-nucleoside
