# Supplementary material for: Preclinical characterization and anti-SARS-CoV-2 efficacy of ATV014: an oral cyclohexanecarboxylate prodrug of 1′-CN-4-aza-7,9-dideazaadenosine C-nucleoside
Source: Signal Transduct Target Ther. 2023 Jan 12;8:27. doi: 10.1038/s41392-023-01310-0 (PMC9835026; doi:10.1038/s41392-023-01310-0)

Supplementary Materials for

**Preclinical characterization and anti-SARS-CoV-2 efficacy of ATV014: Oral cyclohexane****carboxylate prodrug of 1′-CN-4-aza-7,9-dideazaadenosine C-nucleoside**

Qifan Zhou, Sidi Yang, Liu Cao, Yang Yang, Tiefeng Xu, Qishu Chen, Hongzhou Lu, Yingjun Li, Deyin Guo, and Xumu Zhang

Correspondence to: [zhangxm@sustech.edu.cn](mailto:zhangxm@sustech.edu.cn) (X.M.Z.), [guodeyin@mail.sysu.edu.cn](mailto:guodeyin@mail.sysu.edu.cn) (D.Y.G.) and liyj@sustech.edu.cn (Y.J.L.).

**This PDF file includes:**

Synthetic Procedures

Materials and Methods

Supplementary Table. S1 to S11

Supplementary Fig. S1 to S3

Spectrums of chemically synthesized compounds

## Synthetic Procedures

^a^ Reagents and conditions: i) 2,2-Dimethoxypropane, *p*-TsOH, DCM, rt, 8 h; ii) RCOOH, DIC, DMAP, ACN, rt, 12 h; iii) HCOOH, water, rt, 36 h.

**Scheme S1.**  Synthesis of GS-441524 5’-ester prodrugs.

**General procedure**

All reagents used were commercially available. Reactions were monitored by thin-layer chromatography (TLC) on glass plates coated with silica gel with a fluorescent indicator (GF254). Flash silica gel column chromatography was performed using Tsingdao silica gel (60, particle size 300-400 mesh). All the ^1^H NMR and ^13^C NMR spectra were recorded on a Bruker 400 MHz or 600 MHz spectrometer. Chemical shifts (d) were expressed in parts per million using tetramethylsilane as an internal reference. High-resolution mass spectra (HRMS) were measured with an Agilent Accurate-Mass Q-TOF 6530 in ESI mode (Agilent, Santa Clara, CA, USA). HPLC analyses were performed using a Hewlett Packard Model HP 1100 Series instruments, the compounds are at least ≥ 95% pure (OD-3; eluent, *n*-hexane/isopropanol = 80/20; flow rate 0.8 mL/min; temperature 30 °C; wavelength 254 nm; HPLC analysis data are reported in relative area % and were not adjusted to weight %). Melting points were measured on X-4 digital melting point instrument.

**Synthesis of (3*aR*,4*R*,6*R*,6*aR*)-4-(4-aminopyrrolo[2,1-*f*][1,2,4]triazin-7-yl)-6-(hydroxymethyl)-2,2-dimethyltetrahydrofuro[3,4-*d*][1,3]dioxole-4-carbonitrile (2)**

To a solution of **GS-441524** (4.0 g, 13.7 mmol) in dichloromethane (20 mL) was added 2,2-dimethoxypropane (8.9 g, 85.5 mmol). Then *p*-toluenesulfonic acid (2.9 g, 16.8 mmol) was added and the mixture was stirred at room temperature. After 8 h, the reaction was completed as monitored by TLC. The mixture was quenched with heptane (20 mL) and stirred for additional 2 h. The suspension was filtered and the filter was washed with saturated solution of sodium carbonate. After drying the product at 45 ℃ in the oven, intermediate **2** was obtained as a white solid (4.2 g, 93.3% yield). ^1^H NMR (400 MHz, Chloroform-*d*) δ 7.93 (s, 1H), 7.08 (d, *J* = 4.6 Hz, 1H), 6.66 (d, *J* = 4.9 Hz, 1H), 5.98 (s, 2H), 5.43 (d, *J* = 6.5 Hz, 1H), 5.24 (dd, *J* = 6.6, 2.3 Hz, 1H), 4.67 (q, *J* = 1.9 Hz, 1H), 4.04 – 3.74 (m, 2H), 1.81 (s, 3H), 1.40 (s, 3H).

**General procedure for preparation of compounds 3a~m.**

To a solution of intermediate **2** (1.50 g, 4.5 mmol), corresponding acid (4.5 mmol), 4-dimethylaminopyridine (55.40 mg, 0.45 mmol) in DCM (15 mL) was added *N*,*N'*-diisopropylcarbodiimide (0.62 g, 4.9 mmol). The mixture was stirred at room temperature for 12 h. The suspension was filtered and the solvent was washed with 30 mL of saturated solution of Na_2_CO_3_ and then with 30 mL of an aqueous solution of citric acid (20 % w/v). The organic layer was dried over anhydrous Na_2_SO_4_. After removal of the solvent *in vacuo*, the residue was purified by column chromatography (PE/EA = 1:1).

((3*aR*,4*R*,6*R*,6*aR*)-6-(4-aminopyrrolo[2,1-*f*][1,2,4]triazin-7-yl)-6-cyano-2,2-dimethyltetrahydrofuro[3,4-*d*][1,3]dioxol-4-yl)methyl cyclohexanecarboxylate (**3a**). **3a** was obtained as a white solid (1.9 g, 98% yield).

((3*aR*,4*R*,6*R*,6*aR*)-6-(4-aminopyrrolo[2,1-*f*][1,2,4]triazin-7-yl)-6-cyano-2,2-dimethyltetrahydrofuro[3,4-*d*][1,3]dioxol-4-yl)methyl cyclobutanecarboxylate (**3b**). **3b** was obtained as a white solid (1.7 g, 94% yield).

((3*aR*,4*R*,6*R*,6*aR*)-6-(4-aminopyrrolo[2,1-*f*][1,2,4]triazin-7-yl)-6-cyano-2,2-dimethyltetrahydrofuro[3,4-*d*][1,3]dioxol-4-yl)methyl cycloheptanecarboxylate (**3c**). **3c** was obtained as a white solid (1.9 g, 91% yield).

((3*aR*,4*R*,6*R*,6*aR*)-6-(4-aminopyrrolo[2,1-*f*][1,2,4]triazin-7-yl)-6-cyano-2,2-dimethyltetrahydrofuro[3,4-*d*][1,3]dioxol-4-yl)methyl-1-methylcyclohexane-1-carboxylate (**3d**). **3d** was obtained as a white solid (1.7 g, 85% yield).

((3*aR*,4*R*,6*R*,6*aR*)-6-(4-aminopyrrolo[2,1-*f*][1,2,4]triazin-7-yl)-6-cyano-2,2-dimethyltetrahydrofuro[3,4-*d*][1,3]dioxol-4-yl)methyl(1*R*,4*R*)-bicyclo[2.2.1]hept-5-ene-2-carboxylate (**3e**). **3e** was obtained as a white solid (1.8 g, 91% yield).

((3*aR*,4*R*,6*R*,6*aR*)-6-(4-aminopyrrolo[2,1-*f*][1,2,4]triazin-7-yl)-6-cyano-2,2-dimethyltetrahydrofuro[3,4-*d*][1,3]dioxol-4-yl)methyl(3*R*,5*R*,7*R*)-adamantane-1-carboxylate (**3f**). **3f** was obtained as a white solid (2.0 g, 89% yield).

((3*aR*,4*R*,6*R*,6*aR*)-6-(4-aminopyrrolo[2,1-*f*][1,2,4]triazin-7-yl)-6-cyano-2,2-dimethyltetrahydrofuro[3,4-*d*][1,3]dioxol-4-yl)methyl tetrahydrofuran-3-carboxylate (**3g**). **3g** was obtained as a white solid (1.8 g, 93% yield).

((3*aR*,4*R*,6*R*,6*aR*)-6-(4-aminopyrrolo[2,1-*f*][1,2,4]triazin-7-yl)-6-cyano-2,2-dimethyltetrahydrofuro[3,4-*d*][1,3]dioxol-4-yl)methyl-tetrahydro-2H-pyran-4-carboxylate (**3h**). **3h** was obtained as a white solid (1.9 g, 94% yield).

((3*aR*,4*R*,6*R*,6*aR*)-6-(4-aminopyrrolo[2,1-*f*][1,2,4]triazin-7-yl)-6-cyano-2,2-dimethyltetrahydrofuro[3,4-*d*][1,3]dioxol-4-yl)methyl 1-methylpiperidine-4-carboxylate (**3i**). Compound **3i** was prepared using the general method, but after reaction completed, the mixture was filtered and the solution was directly removed *in vacuo* without any washing. The crude product was purified by column chromatography (DCM/MeOH/Et_3_N = 20:1:0.2) to give **3i** as a white solid (1.4 g, 67% yield).

((3*aR*,4*R*,6*R*,6*aR*)-6-(4-aminopyrrolo[2,1-*f*][1,2,4]triazin-7-yl)-6-cyano-2,2-dimethyltetrahydrofuro[3,4-*d*][1,3]dioxol-4-yl)methyl 1-methylpiperidine-2-carboxylate (**3j**). Compound **3j** was prepared using a procedure analogous to the synthesis of compound **3i**. **3j** was obtained as a white solid (1.2 g, 60% yield).

((3*aR*,4*R*,6*R*,6*aR*)-6-(4-aminopyrrolo[2,*1-f*][1,2,4]triazin-7-yl)-6-cyano-2,2-dimethyltetrahydrofuro[3,4-*d*][1,3]dioxol-4-yl)methyl pentadecanoate (**3k**). **3k** was obtained as a white solid (2.1 g, 82% yield).

((3*aR*,4*R*,6*R*,6*aR*)-6-(4-aminopyrrolo[2,*1-f*][1,2,4]triazin-7-yl)-6-cyano-2,2-dimethyltetrahydrofuro[3,4-*d*][1,3]dioxol-4-yl)methyl palmitate (**3l**). **3l** was obtained as a white solid (2.0 g, 79% yield).

((3*aR*,4*R*,6*R*,6*aR*)-6-(4-aminopyrrolo[2,*1-f*][1,2,4]triazin-7-yl)-6-cyano-2,2-dimethyltetrahydrofuro[3,*4-d*][1,3]dioxol-4-yl)methyl nicotinate (**3m**). **3m** was obtained as a white solid (1.3 g, 68% yield).

**General procedure for preparation of compounds 4a~m.**

**((2*R*,3*S*,4*R*,5*R*)-5-(4-aminopyrrolo[2,1-*f*][1,2,4]triazin-7-yl)-5-cyano-3,4-dihydroxytetrahydrofuran-2-yl)methyl cyclohexanecarboxylate (4a).**

Compound **3a** (1.9 g, 4.3 mmol) was added to the mixture of formic acid (13 mL) and water (6.5 mL). The reaction solution was stirred at 30 ^o^C. After 36 h, the reaction was completed as monitored by TLC. The formic acid was removed *in vacuo*, and the resulting residue was dissolved with ethyl acetate (5 mL). Then the pH was adjusted to 7-8 with 50% Na_2_CO_3_ and stirred for at least 1 h at 0~5 ℃. The precipitate was collected by filtration and washed with ethyl acetate to give **4a** as a white solid (1.3 g, 73% yield). M.p. 225−228 ^o^C. HPLC purity: 99.4%. ^1^H NMR (600 MHz, DMSO-*d*_6_) δ 8.01 – 7.79 (m, 3H), 6.92 (d, *J* = 4.5 Hz, 1H), 6.82 (d, *J* = 4.5 Hz, 1H), 6.34 (d, *J* = 6.0 Hz, 1H), 5.38 (d, *J* = 5.9 Hz, 1H), 4.71 (t, *J* = 5.2 Hz, 1H), 4.31 (dd, *J* = 12.2, 2.9 Hz, 1H), 4.26 – 4.20 (m, 1H), 4.15 (dd, *J* = 12.2, 5.0 Hz, 1H), 3.97 (q, *J* = 5.4 Hz, 1H), 2.29 – 2.19 (m, 1H), 1.79 – 1.53 (m, 5H), 1.31 – 1.10 (m, 5H). ^13^C NMR (151 MHz, DMSO-*d*_6_)) δ 175.2, 156.1, 148.4, 124.0, 117.4, 117.0, 110.7, 101.2, 81.7, 79.4, 74.5, 70.6, 63.0, 42.6, 29.0, 28.9, 25.7, 25.2, 25.2. ESI-HRMS: m/z [M+H]^+^ calcd for C_19_H_24_N_5_O_5_: 402.1772; found: 402.1765.

Compounds **4b~m** were synthesized using a procedure analogous to the synthesis of compound **4a**.

**((2*R*,3*S*,4*R*,5*R*)-5-(4-aminopyrrolo[2,1-*f*][1,2,4]triazin-7-yl)-5-cyano-3,4-dihydroxytetrahydrofuran-2-yl)methyl cyclobutanecarboxylate (4b).**

**4b** was obtained as a white solid (0.95 g, 62% yield). M.p. 184−186 ^o^C. HPLC purity: 99.24%. ^1^H NMR (400 MHz, DMSO-*d_6_*) δ 7.93 (s, 1H), 7.90 (br, 2H), 6.93 (d, *J* = 4.5 Hz, 1H), 6.80 (d, *J* = 4.5 Hz, 1H), 6.34 (d, *J* = 5.9 Hz, 1H), 5.38 (d, *J* = 5.8 Hz, 1H), 4.68 (t, *J* = 5.2 Hz, 1H), 4.35-4.31 (m, 1H), 4.25-4.15 (m, 2H), 3.96-3.92 (m, 1H), 3.19-3.11 (m, 1H), 2.15-2.09 (m, 4H), 1.97-1.73 (m, 2H). ^1^H NMR (101 MHz, DMSO-*d_6_*) δ 174.7, 156.1, 148.4, 124.0, 117.4, 117.0, 110.7, 101.3, 81.6, 79.5, 74.5, 70.6, 63.3, 37.6, 25.2, 25.1, 18.2. ESI-HRMS: m/z [M+H]^+^ calcd for C_17_H_20_N_5_O_5_: 374.1459; found: 374.1452.

**((2*R*,3*S*,4*R*,5*R*)-5-(4-aminopyrrolo[2,1-*f*][1,2,4]triazin-7-yl)-5-cyano-3,4-dihydroxytetrahydrofuran-2-yl)methyl cycloheptanecarboxylate (4c).**

**4c** was obtained as a white solid (0.87 g, 50% yield). M.p. 198−200 ^o^C. HPLC purity: 97.75%. ^1^H NMR (600 MHz, DMSO-*d_6_*) δ 7.93 (s, 1H), 7.90 (br, 1H), 6.92 (d, *J* = 4.5 Hz, 1H), 6.81 (d, *J* = 4.5 Hz, 1H), 4.70 (d, *J* = 4.8 Hz, 1H), 4.31-4.29 (m, 1H), 4.24-4.22 (m, 1H), 4.17-4.14 (m, 1H), 3.97-3.95 (m, 1H), 2.46-2.41 (m, 1H), 1.80-1.77 (m, 2H), 1.63-1.37 (m, 10H). ^1^H NMR (151 MHz, DMSO-*d_6_*) δ 176.1, 156.1, 148.4, 124.1, 117.4, 117.0, 110.6, 101.2, 81.7, 79.4, 74.6, 70.6, 63.1, 60.2, 44.4, 30.7, 30.6, 28.2, 28.1, 26.2. ESI-HRMS: m/z [M+H]^+^ calcd for C_20_H_26_N_5_O_5_: 416.1928; found: 416.1920.

**((2*R*,3*S*,4*R*,5*R*)-5-(4-aminopyrrolo[2,1-*f*][1,2,4]triazin-7-yl)-5-cyano-3,4-dihydroxytetrahydrofuran-2-yl)methyl 1-methylcyclohexane-1-carboxylate (4d).**

**4d** was obtained as a white solid (0.51 g, 33% yield). M.p. 104−105 ^o^C. HPLC purity: 98.59%. ^1^H NMR (400 MHz, Methanol-*d*_4_) δ 7.86 (s, 1H), 6.95 – 6.80 (m, 2H), 4.90 – 4.86 (m, 1H), 4.42 – 4.32 (m, 3H), 4.17 (t, *J* = 5.7 Hz, 1H), 2.03 – 1.88 (m, 2H), 1.55 – 1.42 (m, 3H), 1.34 – 1.13 (m, 5H), 1.09 (s, 3H). ^13^C NMR (101 MHz, Methanol-*d*_4_) δ 177.5, 155.9, 146.9, 124.3, 116.6, 116.2, 110.7, 101.1, 82.0, 79.8, 74.3, 70.7, 62.9, 43.1, 35.2, 25.3, 22.9. ESI-HRMS: m/z [M+H]^+^ calcd for C_20_H_26_N_5_O_5_: 416.1928; found: 416.1922.

**((2*R*,3*S*,4*R*,5*R*)-5-(4-aminopyrrolo[2,1-*f*][1,2,4]triazin-7-yl)-5-cyano-3,4-dihydroxytetrahydrofuran-2-yl)methyl (1*R*,4*R*)-bicyclo[2.2.1]hept-5-ene-2-carboxylate (4e).**

The anomers **4e** were obtained as a white solid (1.0 g, 61% yield). M.p. 176−179 ^o^C. HPLC purity: >99%. ^1^H NMR (400 MHz, DMSO-*d_6_*) δ 7.93 (s, 1H), 8.0 (br, 2H), 6.93-6.91 (m, 1H), 6.85-6.82 (m, 1H), 6.35-6.32 (m, 1H), 6.17-6.11 (m, 1H), 5.85-5.78 (m, 1H), 5.40-5.37 (m, 1H), 4.73-4.69 (m, 1H), 4.25-4.09 (m, 3H), 3.99-3.93 (m, 1H), 3.07-2.84 (m, 3H), 1.87-1.75 (m, 1H), 1.35-1.16 (m, 3H). ^13^C NMR (101 MHz, DMSO-*d_6_*) δ 174.0, 156.1, 148.4, 138.0, 136.1, 132.8, 124.0, 117.4, 117.0, 110.7, 101.3, 81.7, 79.3, 74.5, 70.6, 63.5, 49.5, 45.5, 43.0, 42.4, 29.2. ESI-HRMS: m/z [M+H]^+^ calcd for C_20_H_22_N_5_O_5_: 412.1615; found: 412.1607.

**((2*R*,3*S*,4*R*,5*R*)-5-(4-aminopyrrolo[2,1-f][1,2,4]triazin-7-yl)-5-cyano-3,4-dihydroxytetrahydrofuran-2-yl)methyl (3*R*,5*R*,7*R*)-adamantane-1-carboxylate (4f).**

**4f** was obtained as a white solid (1.23 g, 67% yield). M.p. 145−146 ^o^C. HPLC purity: 98.30%. ^1^H NMR (600 MHz, DMSO-*d_6_*) δ 7.93 (s, 1H), 7.90 (br, 2H), 6.93 (d, *J* = 4.5 Hz, 1H), 6.83 (d, *J* = 4.5 Hz, 1H), 6.37 (d, *J* = 5.9 Hz, 1H), 5.37 (d, *J* = 5.9 Hz, 1H), 4.71 (t, *J* = 5.3 Hz, 1H), 4.29-4.23 (m, 2H), 4.16-4.13 (m, 1H), 4.01-3.98 (m, 1H), 1.94-1.91 (m, 3H), 1.71-1.60 (m, 12H). ^13^C NMR (151 MHz, DMSO-*d_6_*) δ 176.7, 156.1, 148.4, 124.1, 117.4, 117.0, 110.5, 101.2, 81.6, 79.2, 74.7, 70.4, 62.7, 38.7, 36.3, 27.7. ESI-HRMS: m/z [M+H]^+^ calcd for C_23_H_28_N_5_O_5_: 454.2085; found: 454.2077.

**((2*R*,3*S*,4*R*,5*R*)-5-(4-aminopyrrolo[2,1-*f*][1,2,4]triazin-7-yl)-5-cyano-3,4-dihydroxytetrahydrofuran-2-yl)methyl tetrahydrofuran-3-carboxylate (4g).**

The anomers **4g** was obtained as a white solid (1.13 g, 69% yield). M.p. 188−192 ^o^C. HPLC purity: 99.20%. ^1^H NMR (600 MHz, DMSO-*d_6_*) δ 7.94 (s, 1H), 7.90 (br, 2H), 6.93 (d, *J* = 4.4 Hz, 1H), 6.82 (d, *J* = 4.4 Hz, 1 H), 6.33 (t, *J* = 5.5 Hz, 1H), 5.40 (d, *J* = 5.8 Hz, 1H), 4.71 (t, *J* = 5.3 Hz, 1H), 4.37-4.34 (m, 1H), 4.25-4.20 (m, 2H), 3.97-3.96 (m, 1H), 3.81-3.78 (m, 1H), 3.75-3.69 (m, 2H), 3.66-3.62 (m, 1H), 3.15-3.10 (m, 1H), 2.08-1.95 (m, 2H). ^13^C NMR (151 MHz, DMSO-*d_6_*) δ 150.1, 148.4, 124.0, 117.4, 117.1, 110.8, 101.3, 81.6, 81.5, 79.5, 74.5, 70.7, 70.6, 69.8, 69.7, 67.8, 63.9, 63.8, 43.4, 29.5, 29.4. ESI-HRMS: m/z [M+H]^+^ calcd for C_17_H_20_N_5_O_6_: 390.1408; found: 390.1401.

**((2*R*,3*S*,4*R*,5*R*)-5-(4-aminopyrrolo[2,1-*f*][1,2,4]triazin-7-yl)-5-cyano-3,4-dihydroxytetrahydrofuran-2-yl)methyl tetrahydro-2H-pyran-4-carboxylate (4h).**

**4h** as obtained as a white solid (1.23 g, 71% yield). M.p. 227−229 ^o^C. HPLC purity: 99.5%. ^1^H NMR (600 MHz, DMSO-*d_6_*) δ 7.93 (s, 1H), 7.90 (br, 1H), 6.92 (d, *J* = 4.6 Hz, 1H), 6.82 (d, *J* = 4.6 Hz, 1H), 6.35 (d, *J* = 5.7 Hz, 1H), 5.41 (d, *J* = 5.6 Hz, 1H), 4.71 (t, *J* = 5.3 Hz, 1H), 4.34-4.32 (m, 1H), 4.25-4.22 (m, 1H), 4.19-4.16 (m, 1H), 3.99-3.96 (m, 1H), 3.79-3.77 (m, 2H), 3.33-3.29 (m, 2H), 2.55-2.50 (m, 1H), 1.68-1.63 (m, 2H), 1.53-1.45 (m, 2H). ^13^C NMR (151 MHz, DMSO-*d_6_*) δ 156.2, 148.5, 124.0, 117.4, 117.1, 110.7, 101.2, 81.6, 79.4, 74.5, 70.6, 66.5, 63.3, 28.8, 28.7. ESI-HRMS: m/z [M+H]^+^ calcd for C_18_H_22_N_5_O_6_: 404.1565; found: 404.1559.

**((2*R*,3*S*,4*R*,5*R*)-5-(4-aminopyrrolo[2,1-*f*][1,2,4]triazin-7-yl)-5-cyano-3,4-dihydroxytetrahydrofuran-2-yl)methyl 1-methylpiperidine-4-carboxylate (4i).**

**4i** was obtained as a white solid (0.38 g, 30% yield). M.p. 133−136 ^o^C. HPLC purity: 95.91%. ^1^H NMR (600 MHz, Methanol-*d*_4_) δ 7.86 (s, 1H), 6.89 (q, *J* = 4.6 Hz, 2H), 4.90 – 4.88 (m, 1H), 4.46 – 4.39 (m, 1H), 4.39 – 4.30 (m, 2H), 4.16 (t, *J* = 5.7 Hz, 1H), 3.02 – 2.86 (m, 2H), 2.45 – 2.33 (m, 6H), 1.97 – 1.86 (m, 2H), 1.82 – 1.65 (m, 2H). ^13^C NMR (151 MHz, Methanol-*d*_4_) δ 175.3, 157.3, 148.4, 125.6, 118.0, 117.7, 112.2, 102.6, 83.4, 81.5, 75.6, 72.2, 64.7, 55.1, 49.5, 49.3, 49.2, 49.0, 48.9, 48.8, 48.6, 45.5, 40.3, 28.1. ESI-HRMS: m/z [M+H]^+^ calcd for C_19_H_25_N_6_O_5_: 417.1881; found: 417.1873.

**((2*R*,3*S*,4*R*,5*R*)-5-(4-aminopyrrolo[2,1-*f*][1,2,4]triazin-7-yl)-5-cyano-3,4-dihydroxytetrahydrofuran-2-yl)methyl 1-methylpiperidine-2-carboxylate (4j).**

The anomers **4j** was obtained as a white solid (0.40 g, 37% yield). M.p. 124−135 ^o^C. HPLC purity: 98.3%. ^1^H NMR (600 MHz, Methanol-*d*_4_) δ 7.86 (s, 1H), 6.90 (d, *J* = 2.5 Hz, 2H), 4.91 – 4.87 (m, 1H), 4.49 – 4.34 (m, 3H), 4.22 – 4.12 (m, 1H), 2.89 (dd, *J* = 11.9, 3.3 Hz, 1H), 2.79 – 2.68 (m, 1H), 2.19 (s, 1H), 2.17 (s, 2H), 2.13 – 2.06 (m, 1H), 1.82 – 1.49 (m, 5H), 1.35 – 1.24 (m, 1H). ^13^C NMR (151 MHz, Methanol-*d*_4_) δ 174.1, 174.0, 157.3, 148.4, 125.7, 118.0, 117.6, 112.2, 102.6, 102.5, 83.3, 81.5, 81.5, 75.6, 72.3, 72.2, 68.6, 64.7, 64.6, 55.9, 44.4, 30.7, 30.6, 26.0, 23.8. ESI-HRMS: m/z [M+H]^+^ calcd for C_19_H_25_N_6_O_5_: 417.1881; found: 417.1874.

**((2*R*,3*S*,4*R*,5*R*)-5-(4-aminopyrrolo[2,1-*f*][1,2,4]triazin-7-yl)-5-cyano-3,4-dihydroxytetrahydrofuran-2-yl)methyl pentadecanoate (4k).**

**4k** was obtained as a white solid (0.78 g, 40% yield). M.p. 126−127 ^o^C. HPLC purity: 98.91%. ^1^H NMR (600 MHz, Methanol-*d*_4_) δ 7.86 (s, 1H), 6.89 (q, *J* = 4.6 Hz, 2H), 4.42 (dd, *J* = 12.0, 3.1 Hz, 1H), 4.37 (m, 1H), 4.31 (dd, *J* = 11.9, 5.2 Hz, 1H), 4.17 – 4.12 (m, 1H), 2.29 (m, 2H), 1.55 (p, *J* = 7.2 Hz, 2H), 1.39 – 1.19 (m, 24H), 0.89 (t, *J* = 7.0 Hz, 3H). ^13^C NMR (151 MHz, Methanol-*d*_4_) δ 175.1, 157.3, 148.3, 125.7, 118.0, 117.6, 112.1, 102.6, 83.4, 81.5, 75.7, 72.1, 64.2, 35.0, 33.1, 30.8, 30.8, 30.8, 30.7, 30.6, 30.5, 30.4, 30.2, 26.0, 23.8, 14.5. ESI-HRMS: m/z [M+H]^+^ calcd for C_27_H_42_N_5_O_5_: 516.3180; found: 516.3173.

**((2*R*,3*S*,4*R*,5*R*)-5-(4-aminopyrrolo[2,1-*f*][1,2,4]triazin-7-yl)-5-cyano-3,4-dihydroxytetrahydrofuran-2-yl)methyl palmitate (4l).**

**4l** was obtained as a white solid (0.71 g, 38% yield). M.p. 125−126 ^o^C. HPLC purity: 99.58%. ^1^H NMR (600 MHz, Methanol-*d*_4_) δ 7.86 (s, 1H), 6.89 (q, *J* = 4.6 Hz, 2H), 4.42 (dd, *J* = 11.9, 3.1 Hz, 1H), 4.38 – 4.34 (m, 1H), 4.32 – 4.27 (m, 1H), 4.16 – 4.08 (m, 1H), 2.36 – 2.24 (m, 2H), 1.60 – 1.50 (m, 2H), 1.36 – 1.24 (m, 24H), 0.89 (t, *J* = 7.0 Hz, 3H). ^13^C NMR (151 MHz, Methanol-*d*_4_) δ 173.7, 155.8, 146.9, 124.3, 116.5, 116.2, 110.7, 101.1, 82.0, 80.1, 74.2, 70.7, 62.8, 33.5, 31.7, 29.4, 29.4, 29.4, 29.4, 29.3, 29.2, 29.1, 29.0, 28.7, 24.6, 22.3, 13.0. ESI-HRMS: m/z [M+H]^+^ calcd for C_28_H_44_N_5_O_5_: 530.3337; found: 530.3329.

**((2*R*,3*S*,4*R*,5*R*)-5-(4-aminopyrrolo[2,1-*f*][1,2,4]triazin-7-yl)-5-cyano-3,4-dihydroxytetrahydrofuran-2-yl)methyl nicotinate (4m).**

**4m** was obtained as a white solid (0.41 g, 35% yield). M.p. 139−141 ^o^C. HPLC purity: 97.37%. ^1^H NMR (600 MHz, Methanol-*d*_4_) δ 9.05 (d, *J* = 2.2 Hz, 1H), 8.73 (dd, *J* = 5.0, 1.7 Hz, 1H), 8.35 – 8.25 (m, 1H), 7.76 (s, 1H), 7.53 (dd, *J* = 8.0, 4.9 Hz, 1H), 6.83 (dd, *J* = 32.8, 4.6 Hz, 2H), 4.97 (d, *J* = 5.3 Hz, 1H), 4.82 – 4.74 (m, 1H), 4.61 – 4.45 (m, 2H), 4.39 – 4.30 (m, 1H). ^13^C NMR (151 MHz, Methanol-*d*_4_) δ 164.5, 155.8, 152.8, 149.8, 146.9, 137.6, 126.2, 124.0, 123.8, 116.6, 116.2, 110.8, 101.1, 81.9, 80.2, 74.1, 70.6, 63.4. ESI-HRMS: m/z [M+H]^+^ calcd for C_18_H_17_N_6_O_5_: 397.1255; found: 397.1250.

## Materials and Method

### Viruses, cells and animals

HEK 293T, other cells were obtained from American Tissue Culture Collection (ATCC). African green monkey kidney Vero E6 cell line (Vero E6) was kindly provided by Dr. Hui Zhang (Sun Yat-sen University). A549-ACE2 cells, HEK 293T and Vero E6, other cells were cultured in DMEM supplemented with 10% fetal bovine serum (FBS), 1% penicillin and streptomycin at 37 °C incubator with 5% CO_2_.

SARS-CoV-2 strain B.1 (hCoV-19/CHN/SYSU-IHV/2020 strain, Accession ID on GISAID: EPI_ISL_444969) was isolated from a sputum sample from a woman admitted to the Eighth People′s Hospital of Guangzhou by Sun Yat-Sen University. The SARS-CoV-2 Beta variant (B.1.351, SARS_CoV-2_human_CHN_20SF18530_2020, Accession ID on GWH: WHBDSE01000000) and Delta variant (B.1.617.2, GDPCC 2.00096) variants were isolated from patients with COVID-19 patients admitted in the Guangzhou Eighth People′s Hospital by Center for Disease Control and Prevention of Guangdong Province. The SARS-CoV-2 Omicron variant (B.1.1.529) was isolated from a patient with COVID-19 patient admitted in to the Shenzhen Third People′s Hospital. All experiments related to SARS-CoV-2 infection were performed at the Biosafety Level 3 (BSL-3) facilities with a license for SARS-CoV-2 study approved by the China National Health Commission.

### SARS-CoV-2 replicon assays

The luciferase assays were carried out according to the manufacturer′s instructions (Promega Corporation, Fitchburg, WI, USA) ^1^. Firstly, the HEK293T cells in 24-well plate transfected with pBAC-SARS-CoV-2-Replicon-Luciferase plasmid (250 ng) and RL-TK plasmid (15 ng). After 6-8 h, the cells were transfected, the supernatant was removed and changed with fresh DMEM medium, followed by adding tested compounds to the media with the final concentration of 10 μM, 5 μM, 2 μM, 1 μM, 0.5 μM, 0.1 μM. After 60 h, cells were lysed in 200 μL Passive Lysis Buffer (PLB). Each lysate (20 μL) was transferred into 96-well white plate and then mixed with 20 μL Luciferase Assay Reagent II, followed by 20 μL of Stop & Glo solution. The luminescence values of the two-step reaction were recorded using a luminescence detector in Synergy H1 Hybrid Multi-Mode Reader (BioTek). Data analysis was performed with GraphPad Prism 8.0 software.

### *In vitro* anti-SARS-CoV-2 activity assays

Vero E6 cells or A549-ACE2 cells were cultured in Dulbecco′s modified Eagle′s medium (DMEM) with 10% fetal bovine serum (FBS) and 1% penicillin–streptomycin antibiotics. The cells were kept at 37 ^o^C under 5% CO_2_ atmosphere. Vero E6 cells were pre-seeded at 2 × 10^4^ cells per well to 48-well plates. Cells were allowed to adhere for 16-24 h and inoculated with SARS-CoV-2 at multiplicity of infection (MOI) of 0.05 for 1 h. The supernatant was removed, and cells were washed with pre-warmed PBS for 2 times, and treated with fresh medium containing gradient concentration of compounds (RDV, GS-441524 and ATV014), or DMSO. After 48 h post infection, the supernatants or cells were collected, antiviral activities were evaluated by quantification of viral copies in the cell supernatant *via* real-time fluorescence quantitative PCR (qRT-PCR). The inhibition rate of compounds was calculated based on the viral copies, and the 50% effective concentration (EC_50_) was calculated using Graphpad Prism 8.0 software. The test results were at least repeated three times.

For SARS-CoV-2 RNA quantification ^2,3^ , total RNA was extracted from cell culture supernatant (or tissues) using Trizol (Invitrogen) with Magbead Viral DNA/RNA Kit (CWBIO) according to the manufacturer′s instructions. Virus was detected by using SARS-CoV-2 nucleic acid detection kit (Da′an Company, Guangzhou, China). For detection of viral genomes, the following primers were used to amplify the genomic RNA for the nucleocapsid protein: 2019-nCoV_N1-F: 5′-AAGAAATTCAACTCCAGGCAGC-3′; 2019-nCoV_N1-R: 5′-GCTGGTTCAATCTGTCAAGCAG -3′; Prb: 5’-TCACCGCCATTGCCAGCCA-3’. The following primers were used to amplify the subgenomic RNA for the E protein: F: 5′- CCAGGTAACAAACCAACAA-3′; R: 5′-TGAGTGAGAGCGGTGAACCAA-3′.

### CCK-8 cell viability assay

To investigate the cytotoxicity of drugs, Vero E6 cells were seeded in a 96-well plate (20,000 cells/well) and were treated with (GS-441524, RDV or ATV014) at gradient concentrations (0, 0.01, 0.1, 1, 5, 10, 50, 75, 100, 200 μM) for 48 hours. Cell viability was tested *via* CCK8 assay kit (CCK-8, Bimake, B34302). CC_50_ values of nucleoside analogs were calculated by using GraphPad Prism 8.0 software.

### *In vivo* anti-SARS-Cov-2 activity in the K18-hACE2 mouse model ^4,5^

The mouse infection experiments of SARS-CoV-2 were performed at the BSL-3 facilities of Guangzhou Customs District Technology Center. For the prophylactic testing, the C57BL/6 (B6.Cg-Tg(K18-ACE2)2Prlmn/J) mice were divided into four groups (n = 4 for each group), the vehicle group, the group receiving ATV014 100 mg/kg orally BID, the group receiving ATV014 300 mg/kg orally BID and the group receiving EIDD-2801 300 mg/kg orally BID. K18-hACE2 mice were anesthetized by inhalation of isoflurane and were intranasally inoculated with SARS-CoV-2 Delta variant (5 × 10^2^ PFU virus per mouse). Meanwhile, mice were treated with vehicle (20% PG+5% solutol+75% ddH_2_O), ATV014 or EIDD-2801 according to group description as described above. Mice were observed for clinical signs daily from day 0 to day 3. At day 3, mice were sacrificed. The lung tissues from euthanized mice were homogenized with DPBS, and the homogenized tissues were centrifuged at 3000 rpm for 10 min at 4 °C. The RNA was extracted from the supernatant for the qRT-PCR testing. The virus titers were determined by FFA (focus forming assay) following a general procedure.

For the transgenic efficacy testing, the C57BL/6 (B6.Cg-Tg(K18-ACE2)2Prlmn/J) mice were divided into seven groups (n = 10 for each group), the vehicle group, the groups receiving ATV014 10/20/50/100/200 mg/kg orally BID and the group receiving EIDD-2801 200 mg/kg BID. After anesthetized by inhalation of isoflurane, the mice were intranasally inoculated with SARS-CoV-2 Delta variant (5 × 10^2^ PFU virus per mouse). Two hours later, the mice were administrated with the vehicle (20% SBE-β-CD + 5% TPGS deionized aqueous solution), ATV014 or EIDD-2801. The remaining steps are similar to the prophylactic testing.

### Permeability in Caco-2 cells

Caco-2 cells were seeded onto polyethylene membranes (PET) in 96-well Falcon insert systems at 2×10^5^ cells/cm^2^ until to 21-28 days for confluent cell monolayer formation. Medium was changed every 3-4 days. Test compounds were diluted with the transport buffer (HBSS without BSA) from a 10 mM stock solution to a concentration of 10 µM and applied to the apical or basolateral side of the cell monolayer. Permeation of the test compounds from A to B direction or B to A direction was determined in duplicate over a 120 minutes incubation at 37°C and 5% CO_2_ with a relative humidity of 95%. In addition, the efflux ratio of each compound was also determined. Test and reference compounds were quantified by LC-MS/MS analysis based on the peak area ratio of analyte/IS. The apparent permeability coefficient Papp (cm/s) was calculated using the equation:

Papp = (dC_r_/d_t_) ×V_r_ / (A×C_0_)

Where dC_r_/dt is the cumulative concentration of compound in the receiver chamber as a function of time (S); V_r_ is the solution volume in the receiver chamber (0.1 mL on the apical side, 0.25 mL on the basolateral side); A is the surface area for the transport, i.e. 0.0804 cm^2^ for the area of the monolayer; C_0_ is the initial concentration in the donor chamber.

The efflux ratio was calculated using the equation:

Efflux Ratio = Papp (BA) / Papp (AB)

Percent recovery was calculated using the equation:

% Recovery = 100 × [(V_r_ × Cr) + (V_d_ ×C_d_)] / (Vd × C_0_)

Where V_d_ is the volume in the donor chambers (0.1 mL on the apical side, 0.25 mL on the basolateral side); C_d_ and C_r_ are the final concentrations of transport compound in donor and receiver chambers, respectively.

### Pharmacokinetic study in SD rats

Male SD rats (180-220 g, N = 3) were fasted for 12 h before drug administration. 4a (ATV014), 4h and 4l were respectively administered intravenously at 5 mg/kg or intragastrically at 25 mg/kg. Blood samples were collected from the jugular vein into anticoagulant EDTA-K2 tubes at 0.083, 0.25, 0.5, 1, 2, 3, 4, 6, 8 and 24 h for the IV group, and 0.25, 1, 0.5, 2, 3, 4, 6, 8 and 24 h for the IG group, respectively. All samples were centrifuged under 4000 rpm/min for 10 min at 4 ^o^C and the plasma (supernatants) were collected and stored at -65 ^o^C for future analysis. An aliquot of 50 μL each plasma sample was treated with 250 μL of acetonitrile. The samples were centrifuged under 4000 rpm/min for 10 min and filtered through 0.2 μm membrane filters. The concentration of analytes in each sample were analyzed by LC/MS/MS. PK parameters were determined following a noncompartmental analysis of the plasma concentration−time data by using Phoenix WinNonlin7.0. The following PK parameters are reported: clearance (CL; L/h/kg), volume of distribution at steady state (Vss; L/kg), terminal half-life (T1/2; h), maximum concentration (Cmax; μM), and area under the concentration−time curve from time 0 to infinity (AUCinf; μM·h). The PK profile test of RDV was conducted following as this protocol, with administration intravenously at 1 mg/kg or intragastrically at 20 mg/kg (N = 3 per group). The PK profile test of ATV014 in CD1 mice was also performed as this protocol, with single intravenous (1.0 mg/kg) and oral (5 and 20 mg/kg) ATV014 (N = 3 per group).

### Preclinical Pharmacokinetic study

The preclinical PK studies of ATV014 in two species (SD rats and Beagle dogs) were conducted at Pharmaron Co., Ltd. according to the NMPA “Guidelines for Nonclinical Pharmacokinetics studies”.

The PK study in SD rats: 30 animals divided into 5 groups (N = 6 for each group with 3 male and 3 female) were fasted for 12 h before dosing. Animals in Group 1 were administered intravenously with ATV014 (dissolved in 20% SBE-β-CD deionized aqueous solution) at a dose of 1 mg/kg. For Group 2-4, ATV014 (dissolved in 20% SBE-β-CD + 5% TPGS deionized aqueous solution) was administered orally at 20 mg/kg, 40 mg/kg and 80 mg/kg, respectively, and the animals in Group 5 received multiple oral doses of ATV014 at 40 mg/kg (BID) for seven days. Blood samples were collected from the jugular vein into EDTA-K2 tubes at various time points post-dose. Serum samples were obtained following general procedures and the concentrations of analytes in the supernatant were analyzed by LC-MS/MS.

The PK study in Beagle dogs: 12 animals divided into 2 groups (N = 6 for each group with 3 male and 3 female) were fasted for 12 h before dosing. For Group 1, ATV014 (dissolved in 20% SBE-β-CD + 5% TPGS deionized aqueous solution) was administered orally at 5 mg/kg, 15 mg/kg and 45 mg/kg, respectively, and administered intravenously with ATV014 (dissolved in 20% SBE-β-CD deionized aqueous solution) at a dose of 1 mg/kg. One week washout period before administration of each dose. Animals in Group 2 received multiple oral doses of ATV014 at 15 mg/kg (BID) for 7 days. Blood samples were collected from the forelimb vein into EDTA-K2 anticoagulant tubes at various time points post-dose. Following general procedures, the serum samples were obtained and the concentrations of analytes in the supernatant were analyzed by LC-MS/MS.

### Preclinical safety evaluation of ATV014

The single dose oral toxicity study and the 14-day repeated dose oral toxicity study in two species (SD rats and beagle dogs) were conducted at Pharmaron Co., Ltd.. The Ames test and rat micronucleus assay were conducted according to NMPA and ICHS7B guidelines.

The single dose oral toxicity study in rats: 2 groups of male and female SD rats (3 animals/sex/group) received a single oral administration of ATV014 (dissolved in 20% SBE-β-CD + 5% TPGS deionized aqueous solution) at dose of 1000 and 2000 mg/kg, and were observed for 4 days before sacrificed. All animals were alive before sacrificed and no abnormal clinical signs were observed during the experimental period at all the doses. There were no drug-related significant changes in body weight and food consumption. After dissection, no morphological change was observed. The acute maximum tolerated single oral gavage dose in SD rats was at least 2000 mg/Kg.

The single dose oral toxicity study in dogs: 4 animals were divided into 2 groups (1 animals/sex/group) received a single oral gavage of ATV014 at dose of 500 mg/kg and 1000 mg/kg, respectively. After 6 days of washout period, the first group was oral-dosed with ATV014 (2000 mg/kg). All groups were observed for 4 days post-dose before sacrificed. In this study, no drug-related clinical morphological changes were noted in all groups. The acute maximum tolerated single oral gavage dose in Beagle dogs was at least 2000 mg/Kg.

The 14-day repeated dose oral toxicity study in rats: Groups of male and female SD rats (8 animals/sex/group) received repeated oral doses of ATV014 (BID) at 0 (vehicle control), 200 (low), 400 (mild) and 800 mg/Kg (high) for 14 days. Animals for TK study (3 animals/sex/group) were sacrificed on Day 15 and after a recovery period of 14 days, the other animals were sacrificed on Day 29. It was found in all animals that abnormal clinical parameter changes including increased creatine kinase (CK), increased albumin (ALB) and albumin/globulin (A/G). Most of the changes were reduced or reversed after a 14-day recovery period. Taken together, dose level of 400 mg/Kg/day may be considered a NOAEL.

The 14-day repeated dose oral toxicity study in dogs: Groups of male and female Beagle dogs (5 animals/sex/group) received repeated oral doses of ATV014 at 0 (vehicle control), 50 (low), 100 (mild) and 200 mg/Kg/day (high) for 14 days. Three-fifths of animals (3 animals/sex/group) were sacrificed on Day 15 and after a recovery period of 14 days, the other animals were sacrificed on Day 29. All animals were alive before sacrificed. Abnormal changes associated with ATV014 included watery stools, increased alkaline phosphatase (ALP), white blood cell count (WBC), neutrophils (ABNEUT), mildly decreased reticulocytes (ABRETIC) and increased adrenal weight. Most of the changes were reversed after a 14-day recovery period for the mild and high dose group. In this study, the NOAEL was 100 mg/Kg/day.

The micronucleus assay in SD rat: Groups of male and female SD rats (5 animals/sex/group) received two oral doses of ATV014 (dissolved in 20% SBE-β-CD + 5% TPGS deionized aqueous solution) at 0 (vehicle control), 200, 800 and 2000 mg/Kg for 24 hours apart. The positive control substance (cyclophosphamide monohydrate, CP) was administered by single intraperitoneal injection a 20 mg/kg. The animals were euthanized for bone marrow collection at 18-24 h after the last dose, and the numbers of polychromatic erythrocytes (PCE) and polychromatic erythrocytes containing micronuclei (MN-PCE) were counted.

The Ames test: In the presence or absence of an exogenous metabolically activated system (*β*-naphthoflavone and phenobarbital induced rat liver S9), we evaluated the ability of ATV014 to induce recovery mutations in Salmonella typhimurium strains (TA98, TA100, TA1535, TA1537) and tryptophan-deficient Escherichia coli (WP2uvrA). The test doses were 5000, 2000, 1000, 500 and 200 μg/dish (corresponding to the test concentration of 50, 20, 10, 5, 2 mg/mL). The corresponding negative (solvent) control and positive control (TA98:2-nitrofluorene; TA1537: Acridine mutant ICR-191; TA100, TA1535 and WP2 uvrA: *N*-methyl-*N*-nitro-*N*-nitroguanidine). At the doses tested, ATV014 with or without the S9 mixture did not induce a mean of reverting mutant colonies greater than two times (for TA98, TA100, and WP2 uvrA) or three times (for TA1535 and TA1537) the mean of the corresponding solvent control group. There was no concentration dependent increase in the number of reverting mutant colonies.

### Metabolism in mouse, rat, dog, monkey and human hepatocytes

ATV014 was incubated in singlicate with incubation media (Williams’ Medium E with 1× GlutaMAX) containing mouse, rat, dog, monkey or human hepatocytes (1 × 10^6^ cells/mL). The incubations were carried out at a final test concentration of 10 µM over a total incubation period of 240 minutes. Samples were taken at 0, 120 and 240 minutes and the reaction terminated by addition of 600 µL of acetonitrile. The supernatants were transferred and placed in the evaporator under steady stream of nitrogen at room temperature until dry. The dried residues were reconstituted with diluents. The control compound verapamil was included in the experiment under the same incubation conditions with the time points taken at 0 min and 240 min. The percentage of verapamil remaining at 240 minutes was used to monitor the metabolic activity of hepatocytes. The samples were analyzed by UPLC-UV-MS/MS. The peak area percentages of parent drug and each metabolite was determined by semi-quantitative estimation using the extracted ion chromatogram peak areas of all detected drug-related material in the sample. Metabolites with percentage of peak area ≥10% are proposed as major metabolites of ATV014.

### Tissue distribution

SD rats were randomly divided into three groups (n = 6, 3 male and 3 male). All rats were intragastrically administered with a single dose of 80 mg/kg ATV014. At 0.25, 1, 8 h post-dosing, the rats were anesthetized, and tissues including small intestine, large intestine, kidney, stomach, liver, spleen, heart, lung, skeletal muscle, epididymis, plasma, testis, subcutaneous fat, brain and ovary tissue were harvested. Blood samples were collected, and part of them were centrifuged to obtain the plasma. Tissue samples were individually homogenized, and the concentrations of the key metabolite GS-441524 in plasma, whole blood and tissue homogenates were analyzed by LC-MS/MS.

### pKa Determination

The pKa value of ATV014 was detected by using a fast-UV Method with Sirius T3 Titrator(Pion, USA). Firstly, the blank calibration and Fast UV buffer calibration were performed: An empty sample vial was placed in sample position. And a full titration of the buffer was performed and reference spectra were collected at all pHs. Then sample was detected as following process: Methanol was used as cosolvent to measure the psKa (cosolvent dissociation constant). 5 μL of the compound stock solution was added to a sample vial. Select three weight ratios of methanol cosolvent and set up the total starting volume at 1.50 mL. The turbidity was detected by spectrometer and the titration range of pH by fast UV method was 2~ 12.

The titration data was obtained from a proper titration and the pKa test result was obtained by Yasuda-Shedlovsky extrapolation method for fast UV psKa method.

### LogP Determination

The stock solution of ATV014 was prepared in DMSO at the concentration of 10 mM or 30 mM. Place 5 µL of stock solution (30 mM) or 15 µL of stock solution (10 mM) of each sample in order into their proper 96-well rack. Add 500 µL of PBS pH 7.4 into each vial of the cap-less LogP plate followed by the addition of 500 µL of 1-octanol. The assay is performed in duplicate. Add one stir stick to each vial and seal using a molded PTFE/Silicone plug. Then transfer the LogP plate to the Eppendorf Thermomixer Comfort plate shaker and shake at 25°C at 1,100 RPM for 1 hour. After completion of incubation, remove plugs and then remove the stir sticks using a big magnet. Transfer the samples to centrifuge tubes and then centrifuge the samples at 25°C at 20,000 g for 20 minutes to separate the phases, and use pipette and syringe to remove the upper (1-octanol) and lower (buffer) phases to the empty tubes, respectively. Take aliquots of 5 µL from upper phases followed by addition of 495 µL of methanol. Vortex for 1 minute, and then take aliquots of 50 µL from the diluent followed by addition of 450 µL of methanol. And take aliquots of 50 µL from lower phases followed by addition of 450 µL of methanol.

The samples were evaluated by LC-MS/MS analysis and all calculations were carried out using Microsoft Excel.

### Statistical analysis

Quantitative experiments were carried out in triplicate and are indicated as the means ± standard deviations (SDs). The statistical tests utilized are two-tailed and respective details have been indicated in figure legends. A p value less than 0.05 was considered statistically significant (*, p-value of ≤ 0.05. **, p-value of ≤ 0.005. ***, p-value of ≤ 0.0005. ****, p-value of ≤ 0.0001).

## Reference

1 Jin, Y. Y. *et al.* A Convenient and Biosafe Replicon with Accessory Genes of SARS-CoV-2 and Its Potential Application in Antiviral Drug Discovery. *Virol Sin* **36**, 913-923, doi:10.1007/s12250-021-00385-9 (2021).

2 Li, K. *et al.* Middle East Respiratory Syndrome Coronavirus Causes Multiple Organ Damage and Lethal Disease in Mice Transgenic for Human Dipeptidyl Peptidase 4. *J Infect Dis* **213**, 712-722, doi:10.1093/infdis/jiv499 (2016).

3 Jung, Y. *et al.* Comparative Analysis of Primer-Probe Sets for RT-qPCR of COVID-19 Causative Virus (SARS-CoV-2). *Acs Infect Dis* **6**, 2513-2523, doi:10.1021/acsinfecdis.0c00464 (2020).

4 Zheng, J. *et al.* COVID-19 treatments and pathogenesis including anosmia in K18-hACE2 mice. *Nature* **589**, 603-607, doi:10.1038/s41586-020-2943-z (2021).

5 Yang, S. D. *et al.* Comparison of model-specific histopathology in mouse models of COVID-19. *J Med Virol* **94**, 3605-3612, doi:10.1002/jmv.27747 (2022).

## Supplementary Tables

### Table S1. Antiviral activities of compounds in SARS-CoV-2 replicon system, the stability of compounds in human plasma and calculated logP value.

|  | | | |
| --- | --- | --- | --- |
| **Compd.** | **R** | **SARS-CoV-2 replicon**  **EC_50_ (μM) ^a^** | **ClogP ^b^** |
| **GS-441524** | —— | 1.644 | -1.43 |
| **4a (ATV014)** |  | 0.48 | 1.075 |
| **4b** |  | 1.05 | 0.163 |
| **4c** |  | 1.23 | 1.532 |
| **4d** |  | 1.39 | 1.492 |
| **4e** |  | 0.44 | 0.307 |
| **4f** |  | 0.99 | 1.505 |
| **4g** |  | 0.48 | -1.015 |
| **4h** |  | 1.61 | -0.694 |
| **4i** |  | 9.37 | -0.424 |
| **4j** |  | 0.64 | 0.008 |
| **4k** |  | 1.16 | 5.09 |
| **4l** |  | 0.95 | 5.547 |
| **4m** |  | 9.12 | -0.537 |

^a^ The analysis of the antiviral effect of compounds were performed through a luciferase-based SARS-CoV-2 replicon system in HEK293T cells. The EC_50_ values were measured with GraphPad Prism Software.

^b^ Octanol-water partition coefficient (logP) was calculated according to the Pharma Algorithms' AB/LogP v2.0 algorithm.

### Table S2. Single-dose PK parameters for 4a, 4h, and 4l in SD rats. ^a^

| **Compd.** | **Route** | **Dose** | **T_1/2_** | **T_max_** | **C_max_** | **AUC_(0-∞)_** | **MRT_(0-∞)_** | **F** |
| --- | --- | --- | --- | --- | --- | --- | --- | --- |
|  |  | mg/kg | h | h | μM/L | h*μM/L | h | % |
| **4a**  (**ATV014**) | po | 25.0 | 1.9±0.7 | 2.7±1.1 | 8.48±0.49 | 20.90±0.00 | 2.51±0.00 | 53.4±0.5 |
|  | iv | 5.0 | 1.7±0.9 | -- ^b^ | 10.41±1.55 | 8.10±1.22 | 0.96±0.07 | -- |
| **4h** | po | 25.0 | 1.36±0.24 | 1.67±2.02 | 2.89±0.62 | 12.17±1.27 | 3.34±0.38 | 33.30±3.01 |
|  | iv ^c^ | 5.0 | 0.94±0.22 | -- | 9.46±2.17 | 7.02±1.55 | 0.92±0.14 | -- |
| **4l** | po | 25.0 | 1.2±0.08 | 2.17±1.76 | 2.82±0.50 | 12.93±1.23 | 3.21±0.30 | 24.74±2.51 |
|  | iv ^c^ | 5.0 | 0.82±0.16 | -- | 21.93±2.85 | 10.25±0.84 | 0.65±0.02 | -- |

^a^ Calculation of PK parameters for **GS-441524** after a single po dose (25 mg/kg) or single iv dose (5 mg/kg) of **4a**, **4h**, and **4l** in SD rats. Results are shown as the mean ± SD, n = 3.

^b^ Not determined (Parameters not determined due to inadequately defined terminal elimination phase).

^c^ For **4h** and **4l**, PK parameters were calculated based on the total concentration of **4h** or **4l** and their metabolite **GS-441524** after a single iv dose (5 mg/kg).

### Table S3. Bidirectional permeability assays of ATV014 and GS-441524 were assessed *in vitro* using the Caco-2 cell model.

| **Compd.** | **P_app_ (cm/s×10^-6^) ^b^** | | **Recovery rate (%)** | | **efflux rate** |
| --- | --- | --- | --- | --- | --- |
|  | **A→B** | **B→A** | **A→B** | **B→A** |  |
| **ATV014** (0.9 μM) ^a^ | < 8.06 | 24.8 | < 121 | 87.5 | > 3.07 |
| **ATV014** (9 μM) ^a^ | 8.69 | 21.8 | 114 | 102 | 2.50 |
| **ATV014** (90 μM) ^a^ | 10.3 | 27.0 | 106 | 112 | 2.61 |
| **GS-441524** (10 μM) | 3.62 | 23.4 | 110 | 105 | 6.46 |

^a^ During the incubation with Caco-2 cells, the prodrug **ATV014** was partially transformed to **GS-441524**, so the parameter calculation was based on the total concentration of **ATV014** and **GS-441524**.

^b^ Papp (A to B) < 2, low permeability; 2 < Papp (A to B) < 10, moderate permeability; Papp (A to B) > 10, high permeability.

### Table S4. Single-dose PK parameters for ATV014 in SD rats (preclinical PK studies). Calculation of PK parameters for GS-441524 following administration of intravenous (1 mg/kg) and oral (20/40/80 mg/kg) (N = 3 per group).

| **Dose** | **Gender** | **T_max_ (hr)** | **C_max_** **(ng/mL)** | **T_1/2_ (hr)** | **AUC_last_ (hr*ng/mL)** | **AUC_inf_ (hr*ng/mL)** | **MRT_last_ (hr)** | **F (%)** |
| --- | --- | --- | --- | --- | --- | --- | --- | --- |
| **1 mg/kg (iv)** | **Male×3** | 0.0833 | 550±62.9 | 0.671 | 398±35.5 | 442 | 0.708±0.0622 | NA^a^ |
|  | **Female×3** | 0.0833 | 612±25.8 | 0.785±0.036 | 411±36.8 | 495±48.0 | 0.700±0.00766 | NA |
|  | **Total** | 0.0833 | 581±54.6 | 0.74±0.074 | 405±33.2 | 474±52.1 | 0.704±0.0399 | NA |
| **20 mg/kg (po)** | **Male×3** | 0.5 | 2163±306 | 1.10±0.0169 | 3533±789 | 3636±806 | 1.59±0.0488 | 44.4±9.91 |
|  | **Female×3** | 0.5 | 2367±316 | 1.30±0.0910 | 4463±480 | 4593±483 | 1.81±0.0808 | 54.2±5.84 |
|  | **Total** | 0.5 | 2265±300 | 1.20±0.124 | 3998±775 | 4115±793 | 1.70±0.135 | 49.3±9.06 |
| **40 mg/kg (po)** | **Male×3** | 0.5 | 3840±306 | 1.45±0.252 | 6132±423 | 6268±452 | 1.77±0.193 | 38.5±2.65 |
|  | **Female×3** | 0.5 | 4903±1177 | 1.17±0.168 | 8024±2201 | 8235±2164 | 1.83±0.114 | 48.8±13.4 |
|  | **Total** | 0.5 | 4372±965 | 1.58±0.240 | 7078±1756 | 7252±1765 | 1.80±0.145 | 43.6±10.3 |
| **80 mg/kg (po)** | **Male×3** | 0.5 | 9113±1590 | 2.27±0.369 | 19541±1745 | 19858±1745 | 1.90±0.0985 | 61.4±5.48 |
|  | **Female×3** | 0.5 | 12933±2650 | 1.88±0.484 | 24346±3733 | 24702±3640 | 1.97±0.180 | 74.0±11.3 |
|  | **Total** | 0.5 | 11023±2863 | 2.08±0.440 | 21943±3704 | 22280±3682 | 1.93±0.315 | 67.7±10.5 |

^a^ NA: the accuracy deviation of this value exceeds ± 15% of the theoretical value (± 20% for the lower limit of quantification), so its accuracy cannot be calculated by Watson.

### Table S5. Single-dose PK parameters for RDV in SD rats. Calculation of PK parameters for GS-441524 following administration of intravenous (1 mg/kg) and oral (20 mg/kg) (N = 3 per group).

| **Dose** | **T_max_ (hr)** | **C_max_** **(ng/mL)** | **T_1/2_ (hr)** | **AUC_0-t_ (hr*ng/mL)** | **AUC_0-∞_ (hr*ng/mL)** | **MRT_0-t_ (hr)** | **F (%)** |
| --- | --- | --- | --- | --- | --- | --- | --- |
| **1 mg/kg (iv)** | 0.0833 | 332±76.1 | 10.3±8.86 | 486±109 | 537±154 | 3.28±0.549 | NA |
| **20 mg/kg (po)** | 2.0 | 256±104 | 4.73±1.20 | 1478±276 | 1517±302 | 4.94±0.0947 | 14.13% |

### Table S6. Single-dose PK parameters for ATV014 in Beagle dogs (preclinical PK studies). Calculation of PK parameters for GS-441524 following administration of intravenous (1 mg/kg) and oral (5/15/45 mg/kg) (N = 3 per group).

| **Dose** | **Gender** | **T_max_ (hr)** | **C_max_** **(ng/mL)** | **T_1/2_ (hr)** | **AUC_last_ (hr*ng/mL)** | **AUC_inf_ (hr*ng/mL)** | **MRT_last_ (hr)** | **F (%)** |
| --- | --- | --- | --- | --- | --- | --- | --- | --- |
| **1 mg/kg (iv)** | **Male×3** | 0.0833 | 897±85.3 | 2.43±0.603 | 1377±259 | 1590±310 | 2.11±0.429 | NA^a^ |
|  | **Female×3** | 0.0833 | 801±44.6 | 2.37±0.249 | 1679±35.5 | 1864±29.1 | 2.45±0.172 | NA |
|  | **Total** | 0.0833 | 849±80.4 | 2.40±0.414 | 1528±234 | 1727±248 | 2.28±0.346 | NA |
| **5 mg/kg (po)** | **Male×3** | 1 | 1517±110 | 2.28 | 6166±1137 | 6772 | 3.44±0.337 | 89.5±16.5 |
|  | **Female×3** | 1 | 1390±233 | 3.30±0.319 | 5403±376 | 6052±448 | 3.31±0.165 | 64.4±4.48 |
|  | **Total** | 1 | 1453±177 | 2.90±0.602 | 5784±865 | 6340±988 | 3.38±0.247 | 76.9±17.5 |
| **15 mg/kg (po)** | **Male×3** | 1 | 4107±56.9 | 4.35±0.595 | 23785±634 | 24256±529 | 5.46±0.126 | 115±3.07 |
|  | **Female×3** | 1 | 4700±165 | 4.76±0.643 | 23939±2697 | 24543±2729 | 5.48±0.556 | 95.1±10.7 |
|  | **Total** | 1 | 529±343 | 4.55±0.598 | 23862±1755 | 24400±1765 | 5.47±0.361 | 105±13.1 |
| **45 mg/kg (po)** | **Male×3** | 1 | 11510±2050 | 4.61±0.327 | 64024±5531 | 65704±5008 | 5.75±0.751 | 103±8.92 |
|  | **Female×3** | 1 | 11317±2532 | 4.42±0.444 | 52468±7199 | 53252±7025 | 4.64±0.452 | 69.4±9.53 |
|  | **Total** | 1 | 11413±2063 | 4.51±0.365 | 58246±8546 | 59478±8734 | 5.19±0.823 | 86.4±20.3 |

^a^ NA: the accuracy deviation of this value exceeds ± 15% of the theoretical value (± 20% for the lower limit of quantification), so its accuracy cannot be calculated by Watson.

### Table S7. Multidose PK parameters for ATV014 in SD Rats (preclinical PK studies). Calculation of PK parameters for GS-441524 after multiple p.o. doses of ATV014 at 40 mg/kg BID (N = 6 per group, M/F=3/3).

| **Day** | **Gender** | **T_max_ (hr)** | **C_max_** **(ng/Ml)** | **AUC_last_ (hr*ng/mL)** | **MRT_last_ (hr)** | **AUC_0-6hr_ (hr*ng/mL)** | **C_avg_ (ng/mL)** | **AUC__tau_**  **(hr*ng/mL)** | **DF** |
| --- | --- | --- | --- | --- | --- | --- | --- | --- | --- |
| **Day 1** | **Male** | 1 | 3157±55.1 | 12340±2226 | 3.40±0.367 | 9452±1395 | NA ^a^ | NA | NA |
|  | **Female** | 0.5 | 4247±118 | 17386±7198 | 4.62±3.10 | 11122±475 | NA | NA | NA |
|  | **Total** | 0.75 | 3702±603 | 14863±5508 | 4.01±2.08 | 10287±1306 | NA | NA | NA |
| **Day 7** | **Male** | 1 | 3210±210 | 9422±772 | NA | 7741±415 | 838 | 20115 | 3.84 |
|  | **Female** | 1 | 4553±647 | 15066±247 | NA | 11819±538 | 1817 | 43608 | 2.6 |
|  | **Total** | 1 | 3882±852 | 12244±3134 | NA | 9780±2275 | 1328±579 | 31861±13899 | 3.22±0.846 |

^a^ NA: the accuracy deviation of this value exceeds ± 15% of the theoretical value (± 20% for the lower limit of quantification), so its accuracy cannot be calculated by Watson.

### Table S8. Multidose PK parameters for ATV014 in Beagle dogs (preclinical PK studies). Calculation of PK parameters for GS-441524 after multiple p.o. doses of ATV014 at 15 mg/kg BID (N = 6 per group, M/F=3/3).

| **Day** | **Gender** | **T_max_ (hr)** | **C_max_** **(ng/mL)** | **AUC_last_ (hr*ng/mL)** | **MRT_last_ (hr)** | **AUC_0-6hr_ (hr*ng/mL)** | **C_avg_ (ng/mL)** | **AUC__tau_ (hr*ng/mL)** | **DF** |
| --- | --- | --- | --- | --- | --- | --- | --- | --- | --- |
| **Day 1** | **Male** | 2 | 3750±145.3 | 39838±4891 | 8.16±0.844 | 16656±381 | NA | NA | NA |
|  | **Female** | 8 | 4833±410 | 50163±3937 | 8.08±0.236 | 19112±1909 | NA | NA | NA |
|  | **Total** | 5 | 4292±654 | 45001±6910 | 8.12±0.556 | 17884±1824 | NA | NA | NA |
| **Day 7** | **Male** | 8 | 5840±308 | 55070±737 | NA | 16960±1062 | 2295±30.7 | 55070±737 | 2.40±0.159 |
|  | **Female** | 8 | 6333±1088 | 55997±5847 | NA | 17303±1243 | 2333±244 | 55997±5847 | 2.58±0.209 |
|  | **Total** | 8 | 6087±765 | 55534±3762 | NA | 17131±1051 | 2314±157 | 55534±3762 | 2.49±0.194 |

### Table S9. The metabolites information of ATV014

| **Peak No.** | **R.T. (min)** | **Meas. m/z**  **[M+H]^+^** | **Biotransformation** | **Percentages peak area at 240 min ^a^** | | | | |
| --- | --- | --- | --- | --- | --- | --- | --- | --- |
|  |  |  |  | **Mouse** | **Rat** | **Dog** | **Monkey** | **Human** |
| M1 | 1.05 | 468.13571 | Hydrolysis + Glucuronidation | 0.11% | - | - | 1.29% | - |
| M2 | 1.07 | 454.15631 | Hydrolysis + Glucose Conjugation | 0.54% | 0.04% | 0.05% | - | - |
| M3 | 2.08 | 292.10391 | Hydrolysis | 93.06% | 90.27% | 92.45% | 96.30% | 95.64% |
| M4 | 3.84 | 320.13538 | Hydrolysis + Methylation | 0.45% | -^b^ | 0.61% | - | 0.04% |
| M5 | 5.28 | 508.16751 | Hydrolysis + De-hydrogenation + Methylation + Glucuronidation | 0.06% | 0.02% | - | 0.60% | 0.04% |
| M6 | 6.18 | 332.13525 | Hydrolysis + De-hydrogenation + Methylation | 0.39% | 1.19% | 0.31% | - | 1.32% |
| P | 7.99 | 402.17715 | Parent drug | 5.39% | 8.47% | 6.58% | 1.81% | 2.95% |

^a^ Relative peak area determined from extracted ion chromatograms of hepatocytes samples at 240 min. *m/z* acquired from full MS scan spectrum of hepatocytes samples.

^b^ “-”: not observed.

### Table S10. PK parameters of GS-441524 in rat plasma and tissues after oral administration of ATV014.

| **Source** | **T_max_** | **C_max_** | **AUC_last_** |
| --- | --- | --- | --- |
|  | **(hr)** | **(ng/g)** | **(hr*ng/g)** |
| Heart | 1.00 | 9100 | 28005 |
| Kidney | 1.00 | 41200 | 137464 |
| Large intestine | 8.00 | 23900 | 180392 |
| Liver | 0.250 | 25017 | 81114 |
| Lung | 0.250 | 31455 | 27361 |
| Skeletal muscle | 1.00 | 6897 | 22729 |
| Small intestine | 0.250 | 125667 | 207435 |
| Spleen | 1.00 | 11633 | 36186 |
| Subcutaneous fat | 1.00 | 4473 | 14396 |
| Brain | 0.250 | 909 | 525 |
| Stomach | 0.250 | 96000 | 106758 |
| Epididymis | 1.00 | 6073 | 22443 |
| Testis | 0.250 | 3910 | 14488 |

### Table S11. The radioligand binding assays of ATV014, GS-441524 and RTP at the concentration of 20 μM in enzymes.

| **Assay Name** | **Spec.** | **% Inhibition** | | |
| --- | --- | --- | --- | --- |
|  |  | **ATV014** | **GS-441524** | **RTP** |
| **ATPase, Na+/K+, Heart, Pig** | **Pig** | -5 | 0 | 0 |
| **Cholinesterase, Acetyl, ACES** | **Hum** | 6 | 16 | 9 |
| **Cyclooxygenase COX-1** | **Hum** | 9 | 3 | 20 |
| **Cyclooxygenase COX-2** | **Hum** | 18 | 11 | 22 |
| **Monoamine Oxidase MAO-A** | **Hum** | -1 | -2 | -5 |
| **Monoamine Oxidase MAO-B** | **Hum** | 13 | 11 | 15 |
| **Peptidase, Angiotensin Converting Enzyme** | **Hum** | -20 | -15 | -25 |
| **Peptidase, CTSG (Cathepsin G)** | **Hum** | 4 | -1 | 2 |
| **Phosphodiesterase PDE3A** | **Hum** | 3 | -5 | 0 |
| **Phosphodiesterase PDE4D2** | **Hum** | 6 | -2 | -4 |
| **Protein Serine/Threonine Kinase, PRKCA (PKCα)** | **Hum** | -10 | -10 | -12 |
| **Protein Tyrosine Kinase, Insulin Receptor** | **Hum** | 1 | -11 | 0 |
| **Protein Tyrosine Kinase, LCK** | **Hum** | 12 | -9 | 18 |
| **Adenosine A1** | **Hum** | 13 | 9 | -4 |
| **Adenosine A2A** | **Hum** | 15 | 9 | 14 |
| **Adrenergic α1A** | **Hum** | 2 | 3 | 0 |
| **Adrenergic α1B** | **Hum** | 9 | 2 | -2 |
| **Adrenergic α1D** | **Hum** | 2 | -22 | 6 |
| **Adrenergic α2A** | **Hum** | 10 | 11 | 7 |
| **Adrenergic α2B** | **Hum** | 3 | -1 | 9 |
| **Adrenergic β1** | **Hum** | 11 | -4 | 14 |
| **Adrenergic β2** | **Hum** | 5 | -1 | 3 |
| **Androgen (Testosterone)** | **Hum** | 15 | 14 | 6 |
| **Angiotensin AT1** | **Hum** | 4 | 12 | -10 |
| **Bradykinin B2** | **Hum** | -3 | -7 | 6 |
| **Calcium Channel L-Type, Benzothiazepine** | **Rat** | -2 | 3 | 8 |
| **Calcium Channel L-Type, Dihydropyridine** | **Rat** | 0 | 2 | 3 |
| **Calcium Channel L-Type, Phenylalkylamine** | **Rat** | -4 | 13 | 5 |
| **Calcium Channel N-Type** | **Rat** | 2 | 3 | 0 |
| **Cannabinoid CB1** | **Hum** | -6 | 10 | 5 |
| **Cannabinoid CB2** | **Hum** | 9 | -7 | 13 |
| **Chemokine CCR1** | **Hum** | -12 | -10 | 5 |
| **Chemokine CXCR2 (IL-8RB)** | **Hum** | -3 | -1 | -6 |
| **Cholecystokinin CCK1 (CCKA)** | **Hum** | -1 | -17 | -1 |
| **Cholecystokinin CCK2 (CCKB)** | **Hum** | 14 | 12 | -5 |
| **Dopamine D1** | **Hum** | 3 | -2 | -7 |
| **Dopamine D2L** | **Hum** | -2 | 2 | 7 |
| **Dopamine D2S** | **Hum** | -1 | 4 | -9 |
| **Endothelin ETA** | **Hum** | -1 | -2 | -2 |
| **Estrogen ERα** | **Hum** | -4 | 1 | -2 |
| **GABAA, Chloride Channel, TBOB** | **Rat** | -7 | -5 | -9 |
| **GABAA, Flunitrazepam, Central** | **Rat** | 11 | 10 | 2 |
| **GABAA, Ro-15-1788, Hippocampus** | **Rat** | -4 | -12 | -5 |
| **Vasopressin V1A** | **Hum** | -3 | -1 | -6 |
| **GABAB1A** | **Hum** | 17 | 6 | -2 |
| **Glucocorticoid** | **Hum** | -3 | 0 | 0 |
| **Glutamate, AMPA** | **Rat** | -5 | -11 | -16 |
| **Glutamate, Kainate** | **Rat** | 2 | 6 | 4 |
| **Glutamate, Metabotropic, mGlu5** | **Hum** | 20 | 13 | 5 |
| **Glutamate, NMDA, Agonism** | **Rat** | 5 | 3 | 2 |
| **Glutamate, NMDA, Glycine** | **Rat** | 0 | -1 | 3 |
| **Glutamate, NMDA, Phencyclidine** | **Rat** | 5 | -5 | 3 |
| **Glutamate, NMDA, Polyamine** | **Rat** | 16 | 7 | 9 |
| **Glycine, Strychnine-Sensitive** | **Rat** | 10 | -15 | -5 |
| **Histamine H1** | **Hum** | -8 | 3 | -8 |
| **Histamine H2** | **Hum** | 4 | -25 | -12 |
| **Leukotriene, Cysteinyl CysLT1** | **Hum** | -11 | 1 | 4 |
| **Melanocortin MC1** | **Hum** | 0 | -4 | -1 |
| **Melanocortin MC4** | **Hum** | 9 | 0 | 6 |
| **Muscarinic M1** | **Hum** | -4 | -1 | 7 |
| **Muscarinic M2** | **Hum** | -1 | -3 | -11 |
| **Muscarinic M3** | **Hum** | 14 | 8 | 16 |
| **Muscarinic M4** | **Hum** | 0 | 6 | 0 |
| **Neuropeptide Y Y1** | **Hum** | 0 | -3 | 13 |
| **Nicotinic Acetylcholine α1, Bungarotoxin** | **Hum** | -2 | 4 | -6 |
| **Nicotinic Acetylcholine α3β4** | **Hum** | -2 | -2 | 5 |
| **Opiate δ1 (OP1, DOP)** | **Hum** | 3 | 9 | 3 |
| **Opiate κ (OP2, KOP)** | **Hum** | 26 | 5 | 6 |
| **Opiate µ (OP3, MOP)** | **Hum** | 17 | 12 | 0 |
| **Platelet Activating Factor (PAF)** | **Hum** | 2 | -6 | -5 |
| **Potassium Channel [KATP]** | **Hum** | 2 | -4 | 4 |
| **Potassium Channel hERG** | **Hum** | -2 | -4 | 6 |
| **PPARγ** | **Hum** | 6 | 6 | 2 |
| **Progesterone PR-B** | **Hum** | 7 | 9 | 3 |
| **Serotonin (5-Hydroxytryptamine) 5-HT1A** | **Hum** | -1 | 15 | 10 |
| **Serotonin (5-Hydroxytryptamine) 5-HT1B** | **Hum** | 2 | 7 | 9 |
| **Serotonin (5-Hydroxytryptamine) 5-HT2A** | **Hum** | 3 | 9 | 12 |
| **Serotonin (5-Hydroxytryptamine) 5-HT2B** | **Hum** | 12 | 12 | 3 |
| **Serotonin (5-Hydroxytryptamine) 5-HT2C** | **Hum** | 8 | 20 | 4 |
| **Serotonin (5-Hydroxytryptamine) 5-HT3** | **Hum** | 2 | 3 | 11 |
| **Sodium Channel, Site 2** | **Rat** | 9 | 14 | 18 |
| **Tachykinin NK1** | **Hum** | 40 | 3 | 9 |
| **Transporter, Adenosine** | **Gp** | 53 | 7 | -13 |
| **Transporter, Dopamine (DAT)** | **Hum** | 26 | 12 | 24 |
| **Transporter, GABA** | **Rat** | 8 | 4 | -2 |
| **Transporter, Norepinephrine (NET)** | **Hum** | 0 | -3 | 4 |
| **Transporter, Serotonin (5-Hydroxytryptamine) (SERT)** | **Hum** | 12 | 18 | 7 |

## Supplementary Figures

**Figure S1.** Schematic diagram of oral prodrug design of cyclic or long-chain carboxylates **GS-441524**.

**Figure S2.** Antiviral activity of **RDV**, **GS-441524**, and **ATV014** against B.1 strain of SARS-CoV-2 in A549-ACE2 cells.

**Figure S3.** The proposed metabolic pathways of **ATV014**.

## Spectrums of chemically synthesized compounds

#### Spectrums of 4a (ATV014)

#### ^1^H NMR of 4a


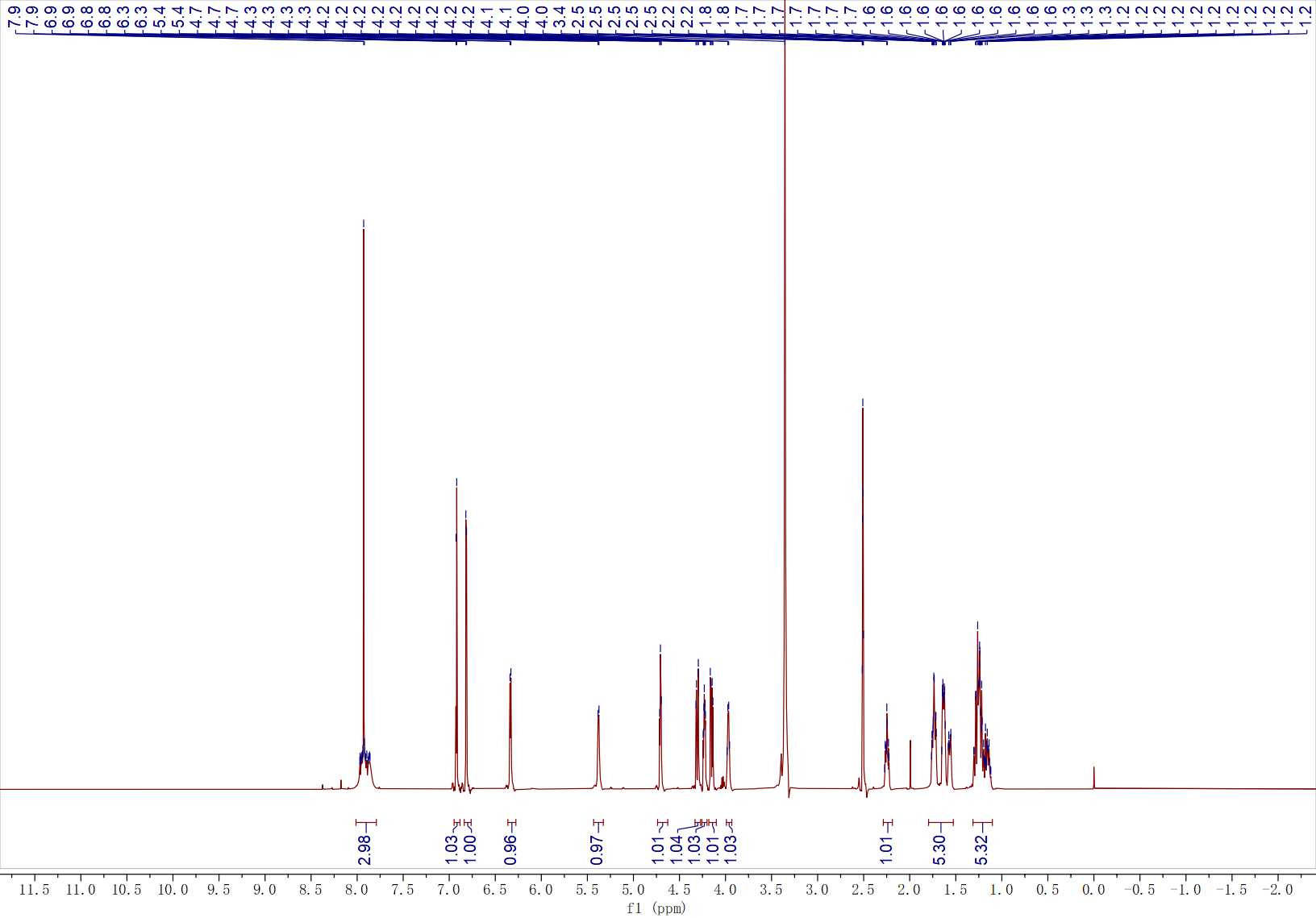


#### ^13^C NMR of 4a


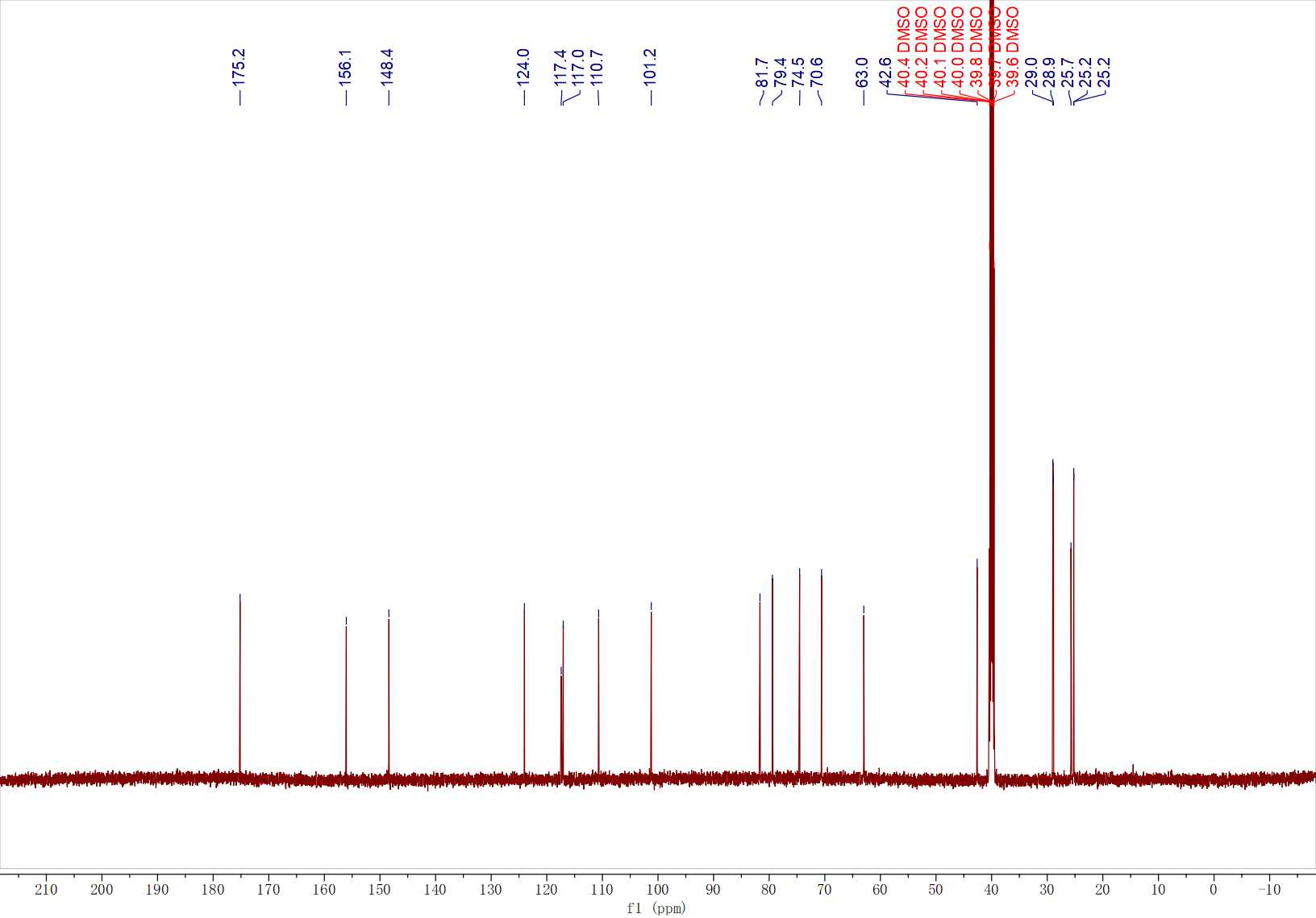


#### ESI-HRMS spectrum of 4a


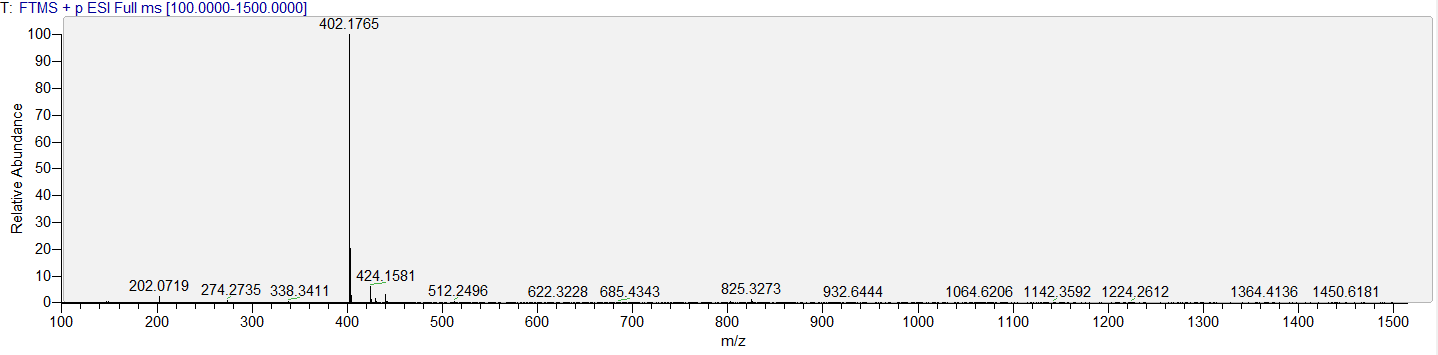


#### HPLC spectrum of 4a


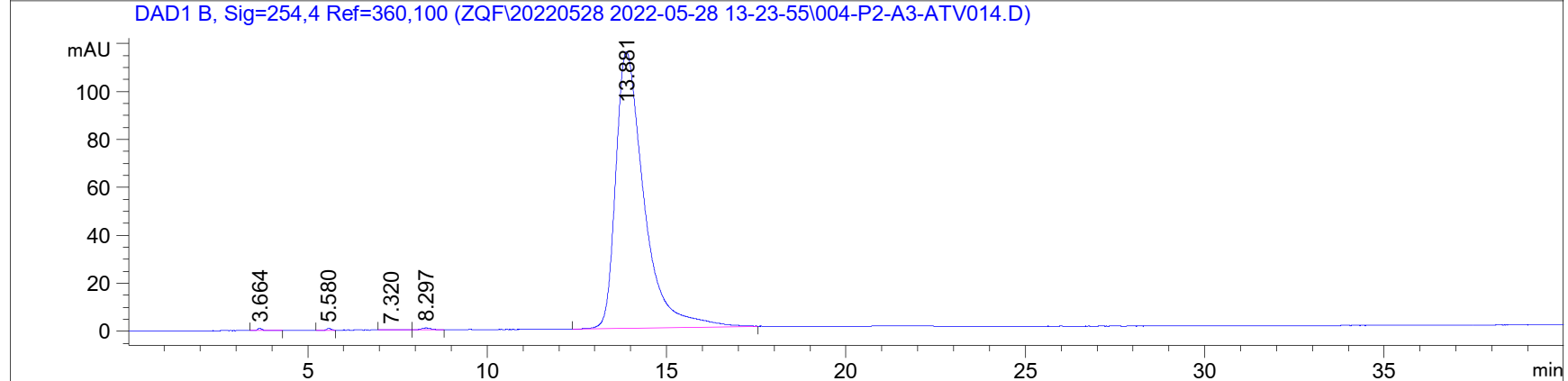


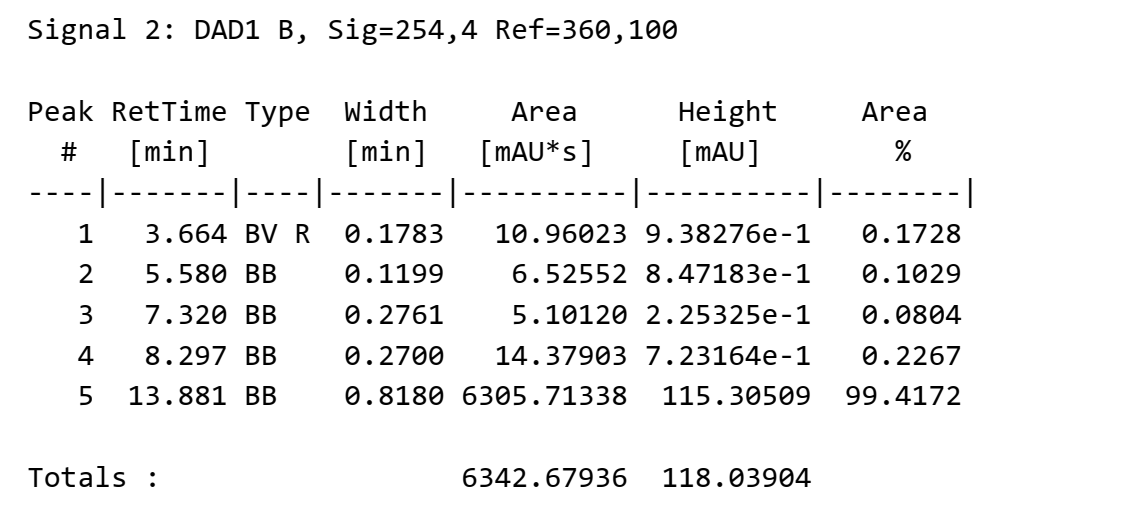


#### Single crystal of 4a


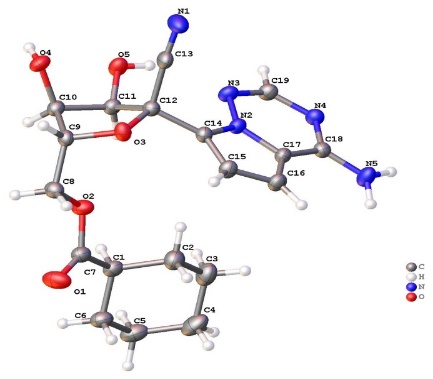


| **Molecular formula** | C_19_H_23_N_5_O_5_ |
| --- | --- |
| **Molecular weight** | 401.42 |
| **T/K** | 180.00 (10) |
| **Crystalline system** | orthorhombic |
| **Space group** | P2_1_2_1_2_1_ |
| **a/Å** | 10.35640 (10) |
| **b/Å** | 10.5219 (2) |
| **c/Å** | 17.3653 (2) |
| **α/°** | 90 |
| **β/°** | 90 |
| **γ/°** | 90 |
| **Unit cell volume /Å^3^** | 1892.28 (5) |
| **Z** | 4 |
| **ρ_calc_g/cm^3^** | 1.409 |
| **μ/mm^‑1^** | 0.868 |
| **F(000)** | 848.0 |
| **Crystal size /mm^3^** | 0.11 × 0.07 × 0.04 |
| **Diffractive light source** | Cu Kα (λ = 1.54184) |
| **2 θ range /°** | 9.828 to 147.012 |
| **Diffraction index range** | -12 ≤ h ≤ 12, -11 ≤ k ≤ 12, -20 ≤ l ≤ 20 |
| **Diffraction number** | 38338 |
| **Independent diffraction number** | 3659 [R_int_ = 0.0405, R_sigma_ = 0.0182] |
| **Data / limits / parameters** | 3659/2/270 |
| **Goodness of fit based on F2** | 1.026 |
| **Final R factor [I>=2σ (I)]** | R_1_ = 0.0280, wR_2_ = 0.0736 |
| **Final R factor [all data]** | R_1_ = 0.0299, wR_2_ = 0.0770 |
| **Peak / valley of maximum residual electron density / e Å^-3^** | 0.14/-0.21 |
| **Flack parameter** | -0.06 (5) |

#### Spectrums of 4b

#### ^1^H NMR of 4b


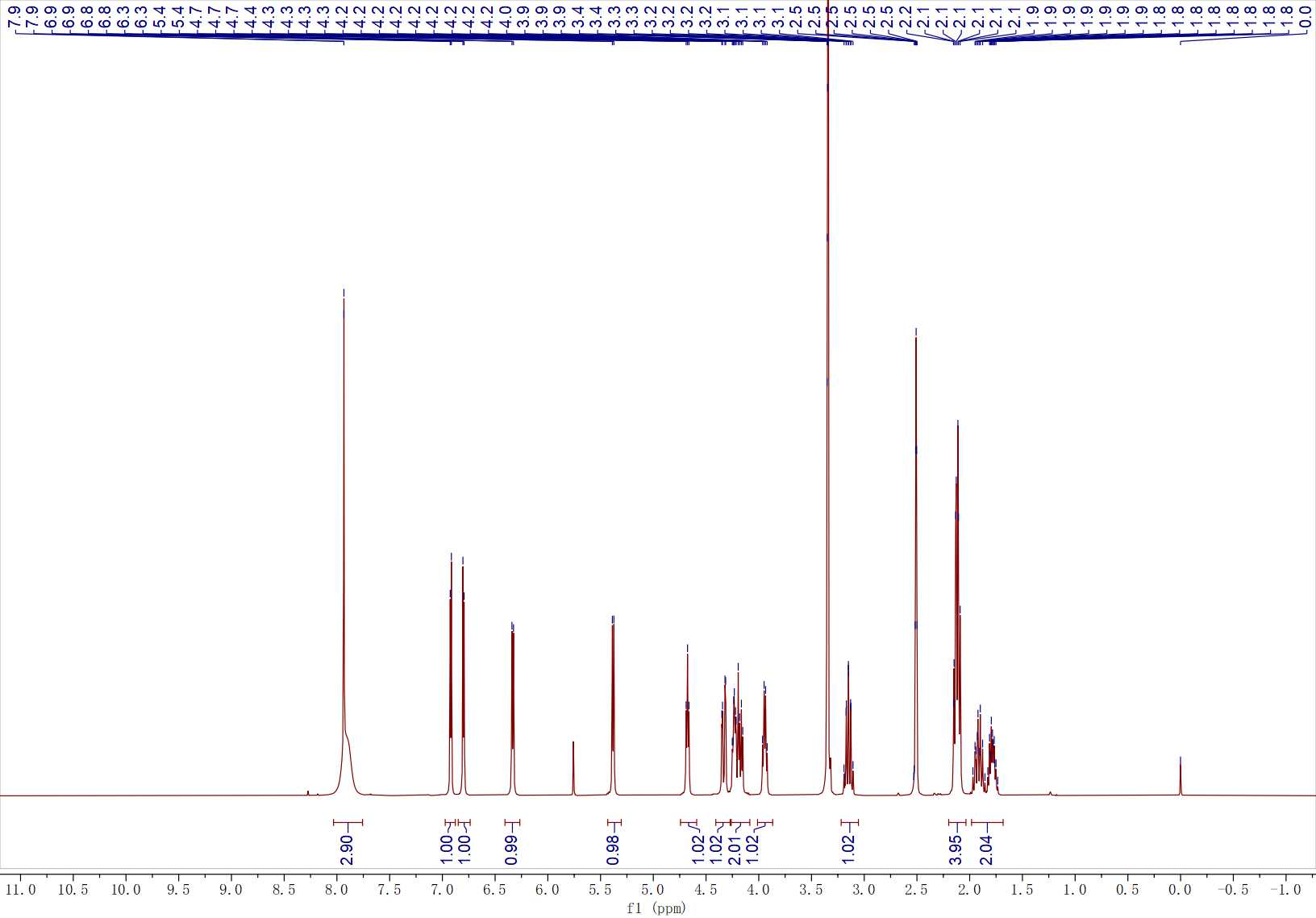


#### ^13^C NMR of 4b


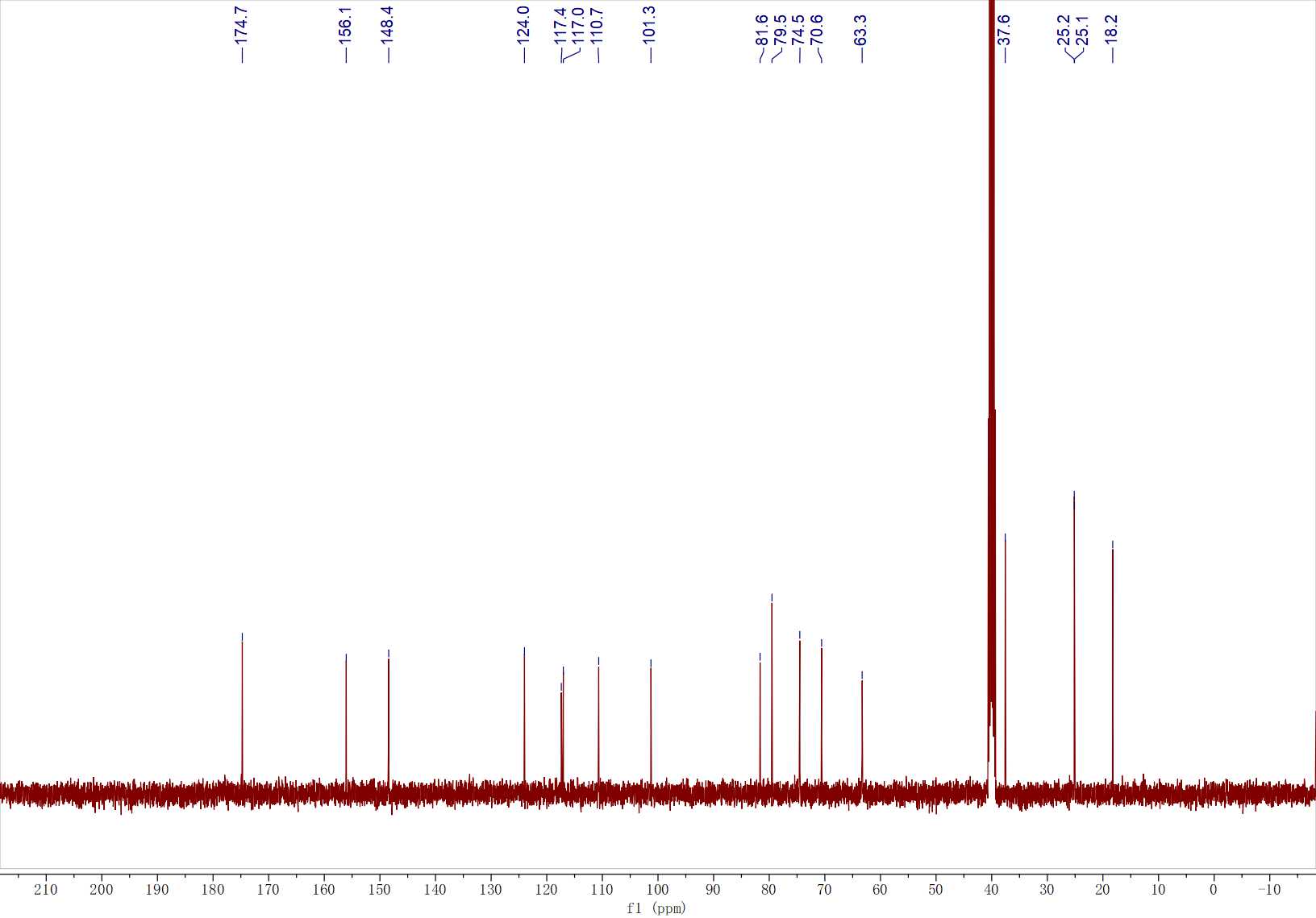


#### ESI-HRMS spectrum of 4b


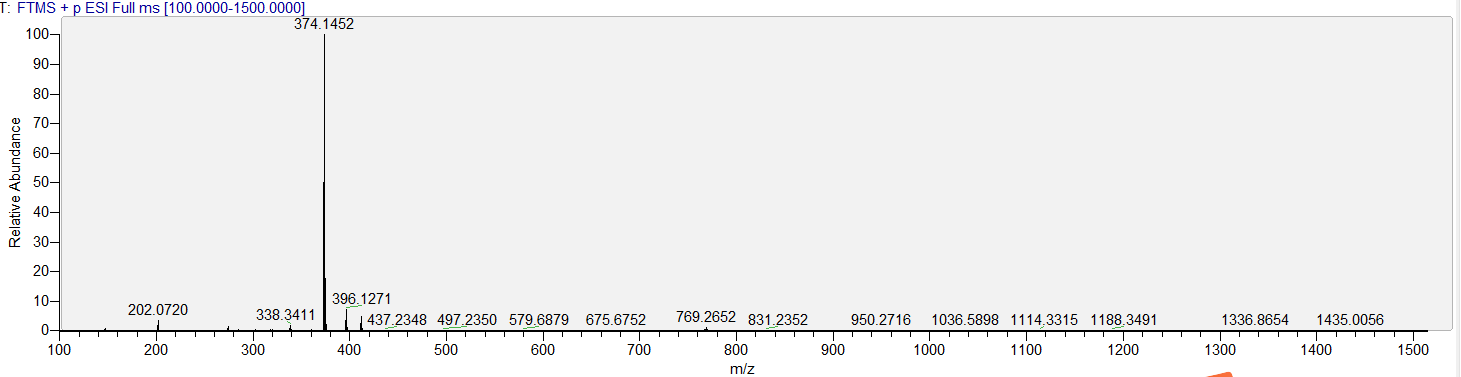


#### HPLC spectrum of 4b


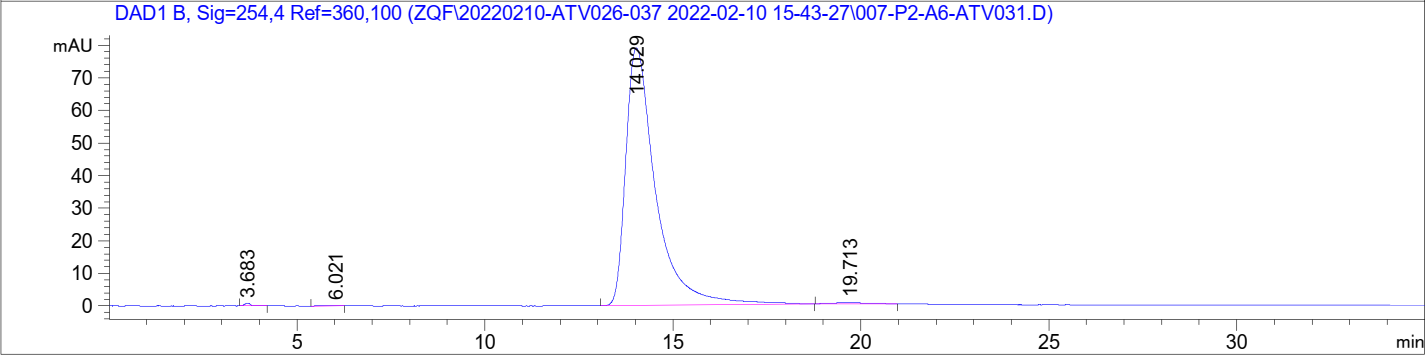


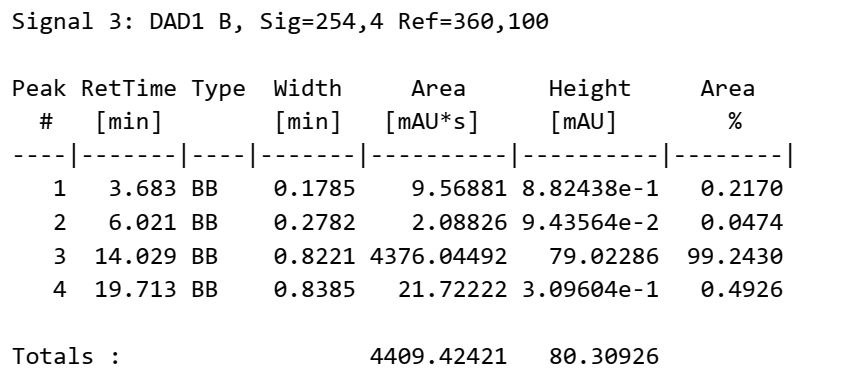


#### Spectrums of 4c

#### ^1^H NMR of 4c


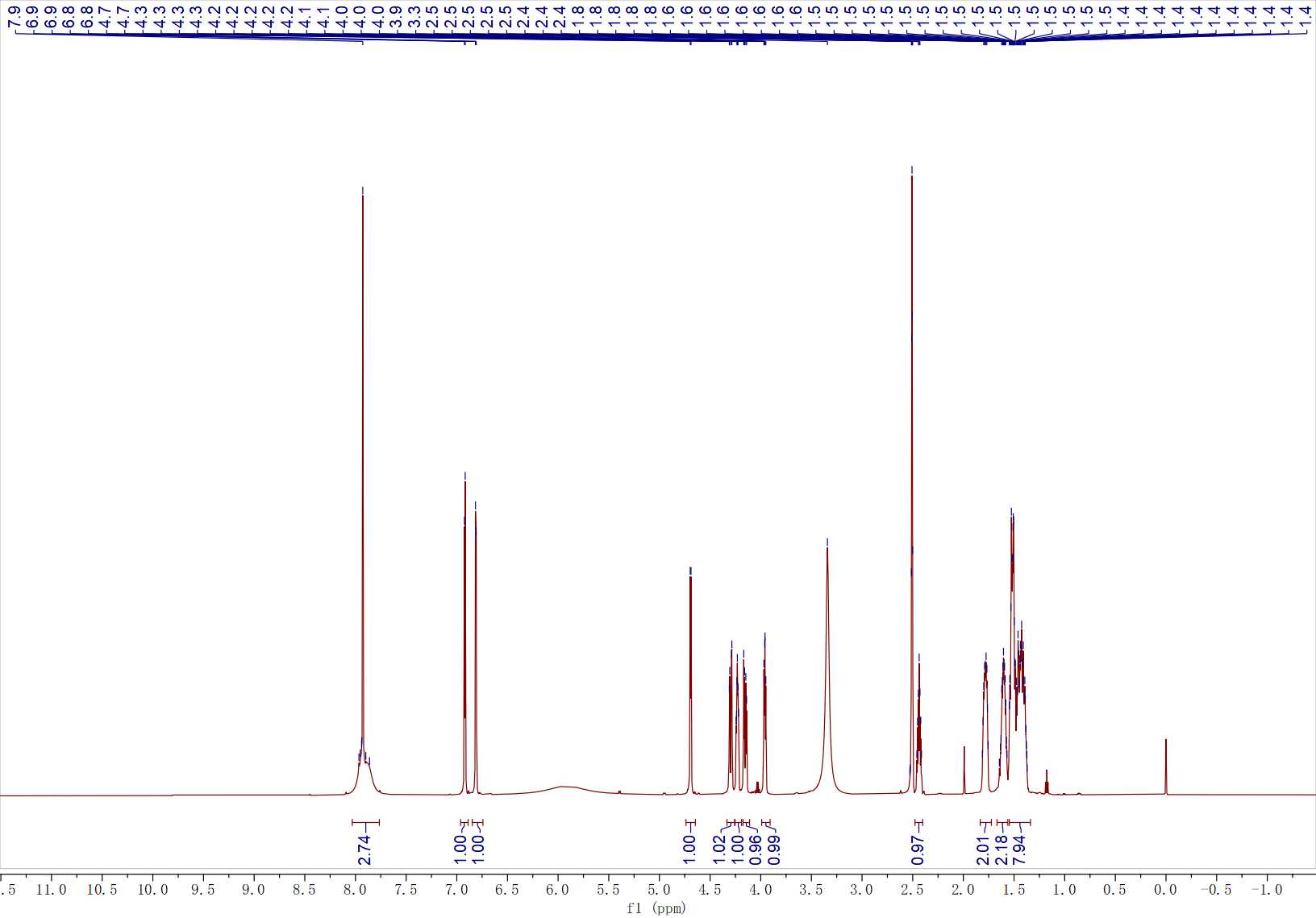


#### ^13^C NMR of 4c


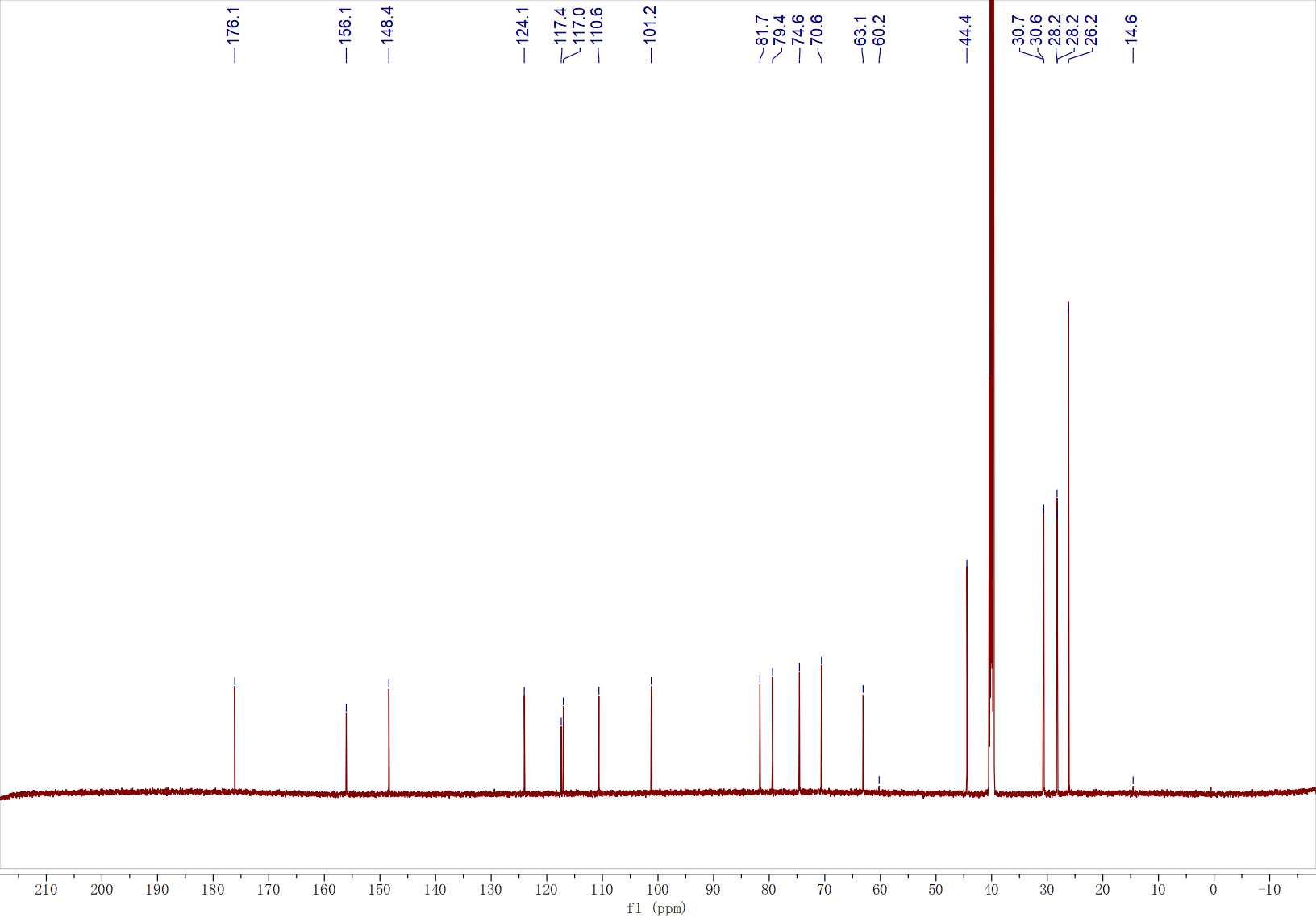


#### ESI-HRMS spectrum of 4c


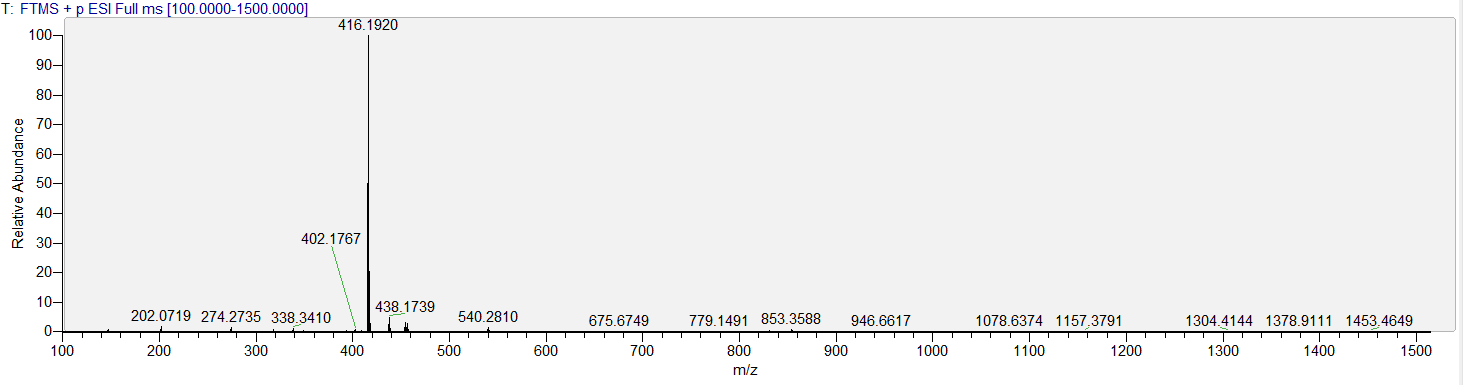


#### HPLC spectrum of 4c


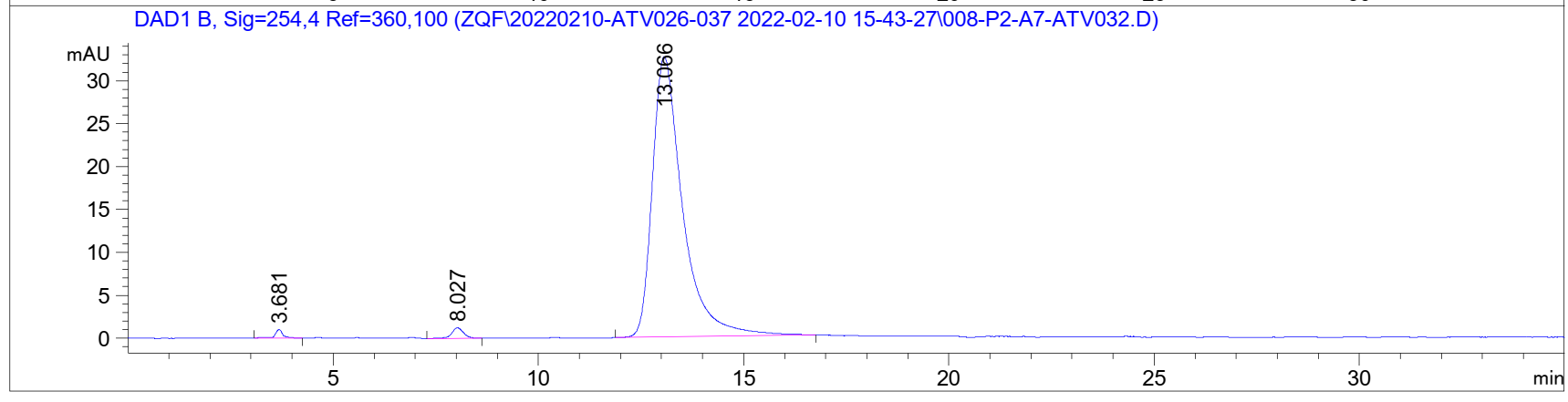


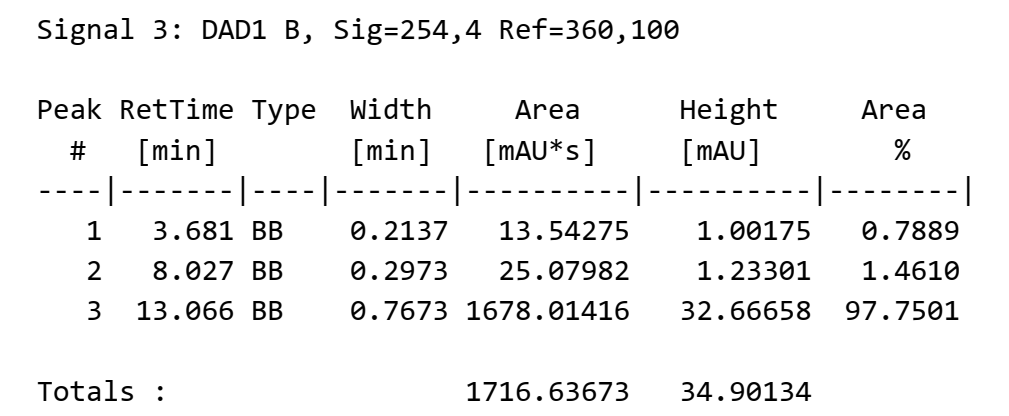


#### Spectrums of 4d

#### ^1^H NMR of 4d


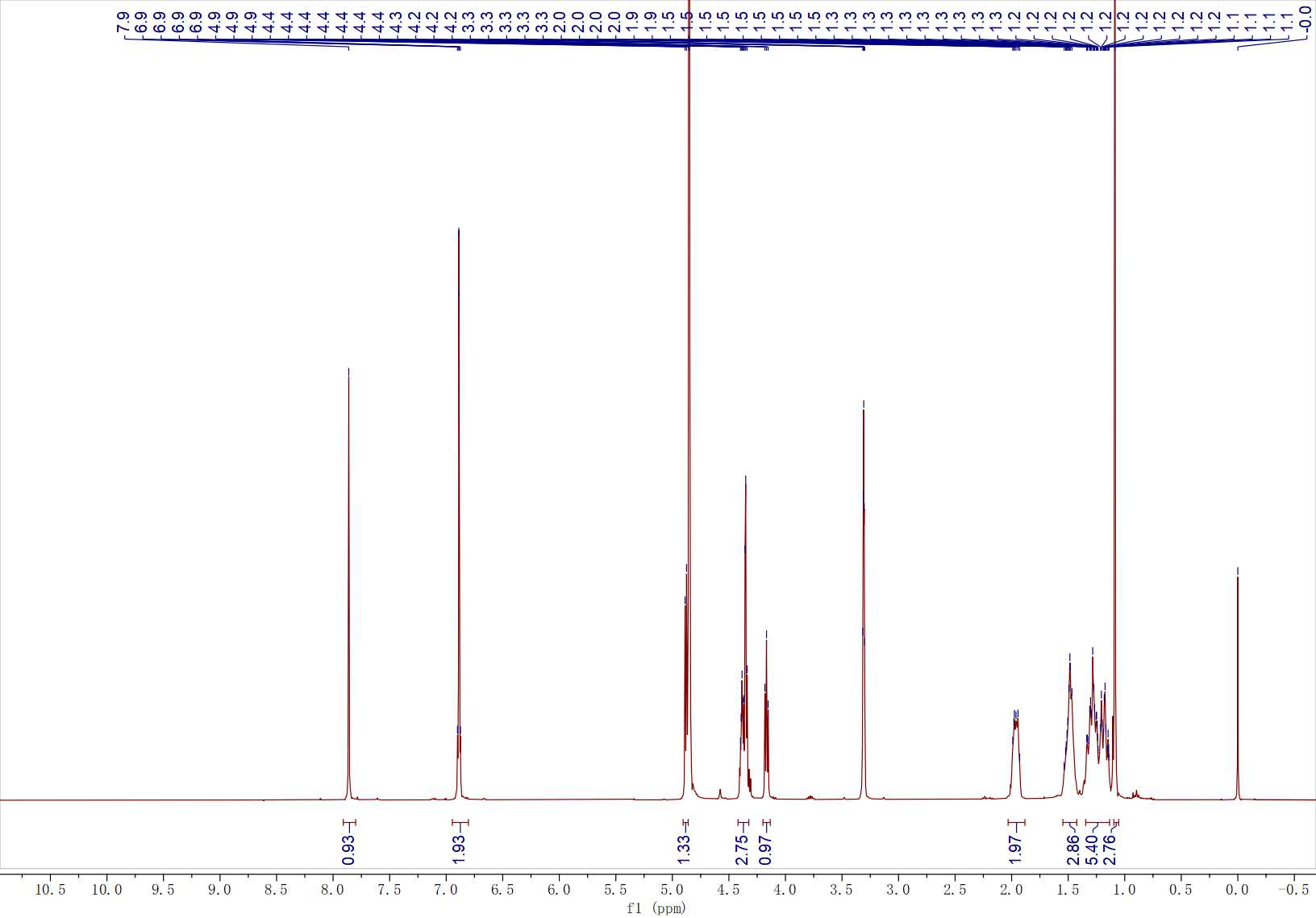


#### ^13^C NMR of 4d


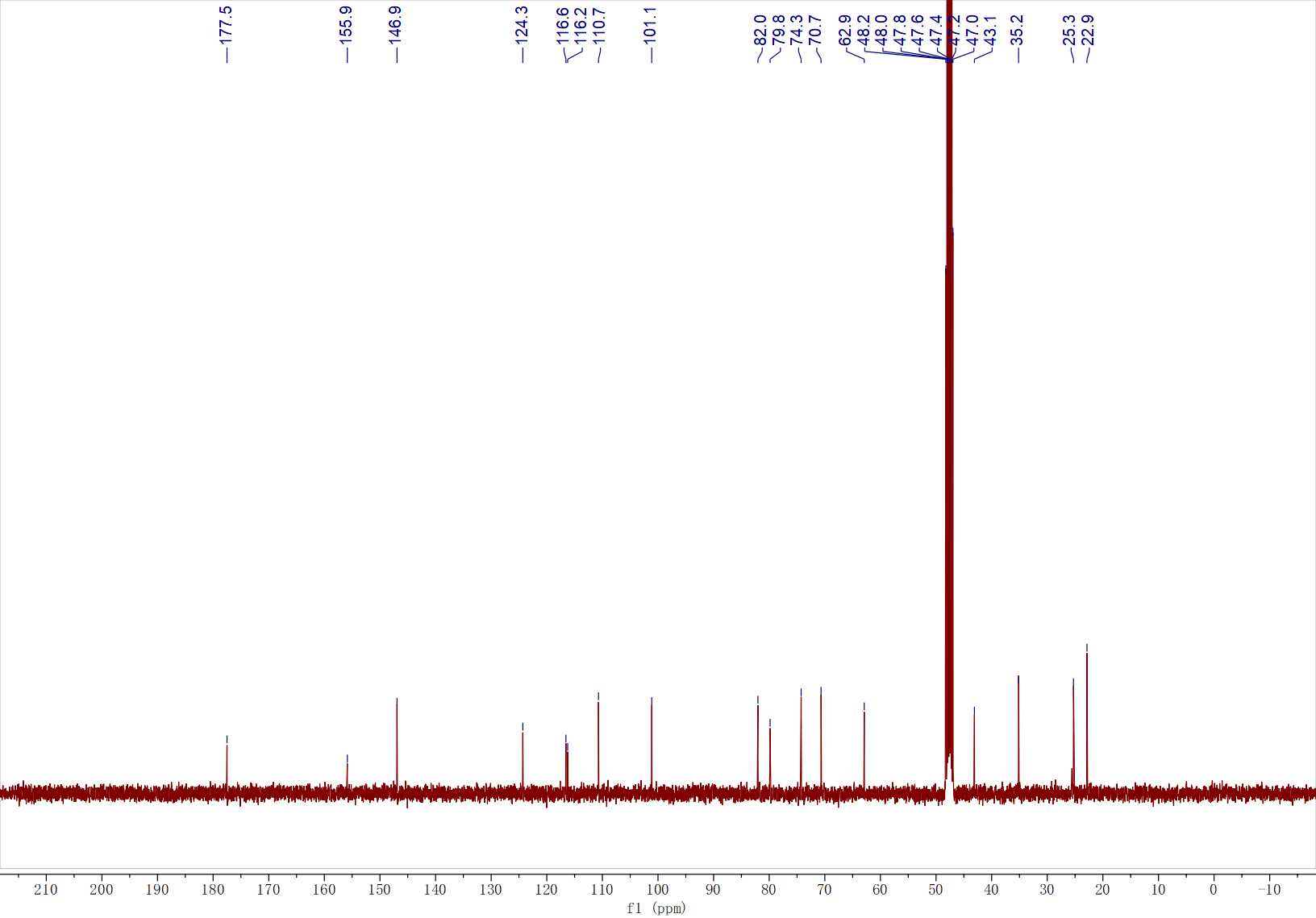


#### ESI-HRMS spectrum of 4d


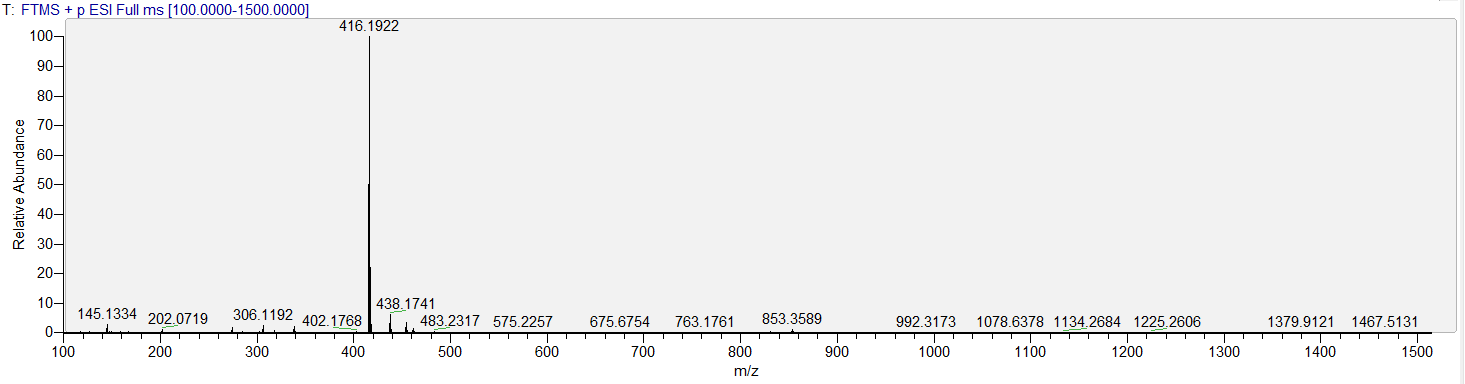


#### HPLC spectrum of 4d


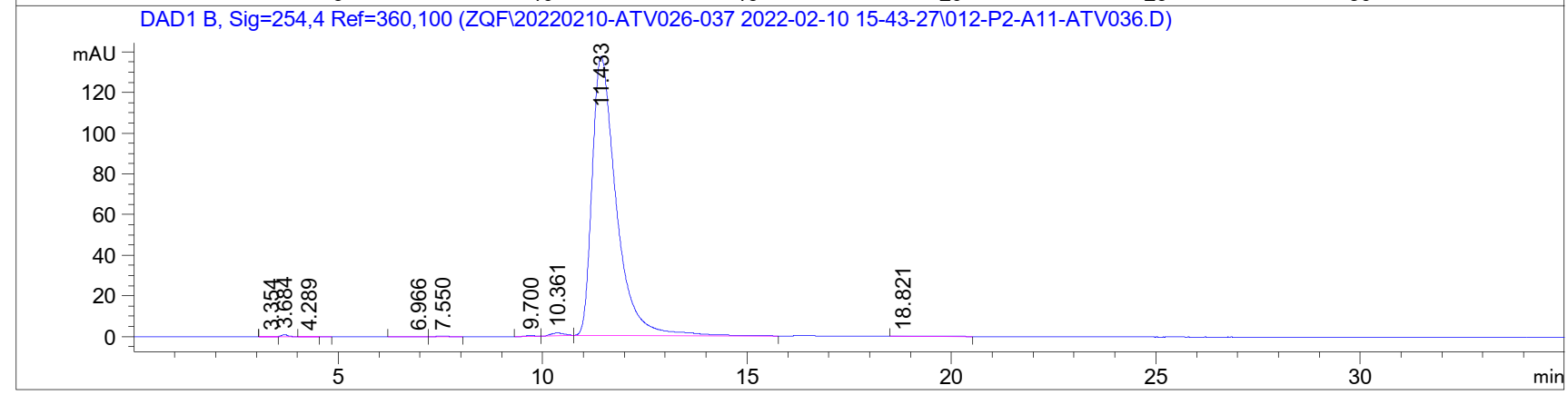


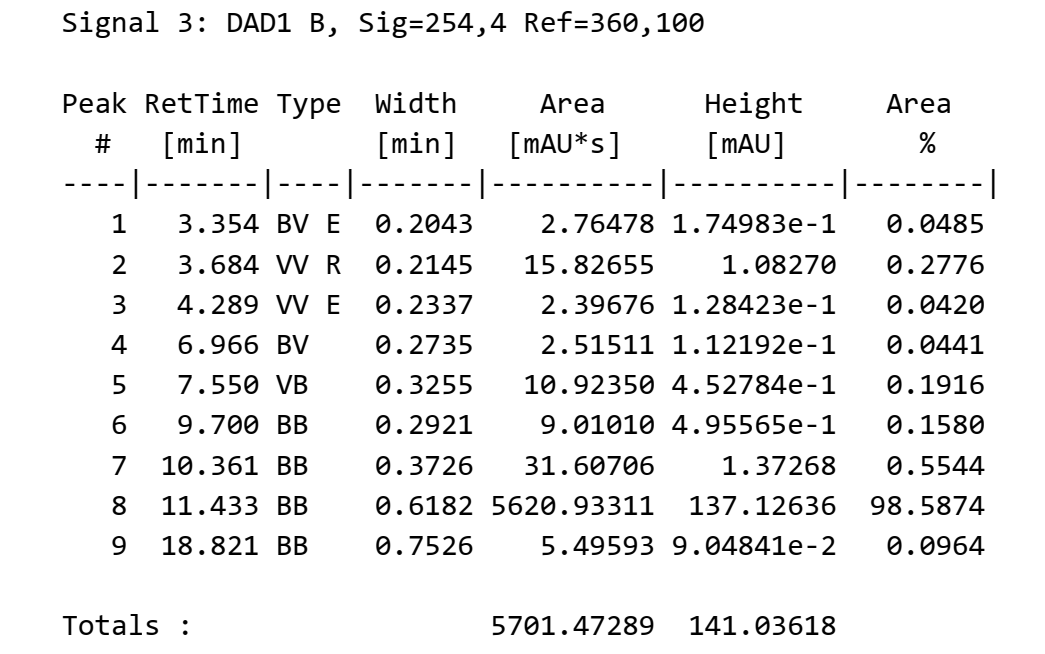


#### Spectrums of 4e

#### ^1^H NMR of 4e


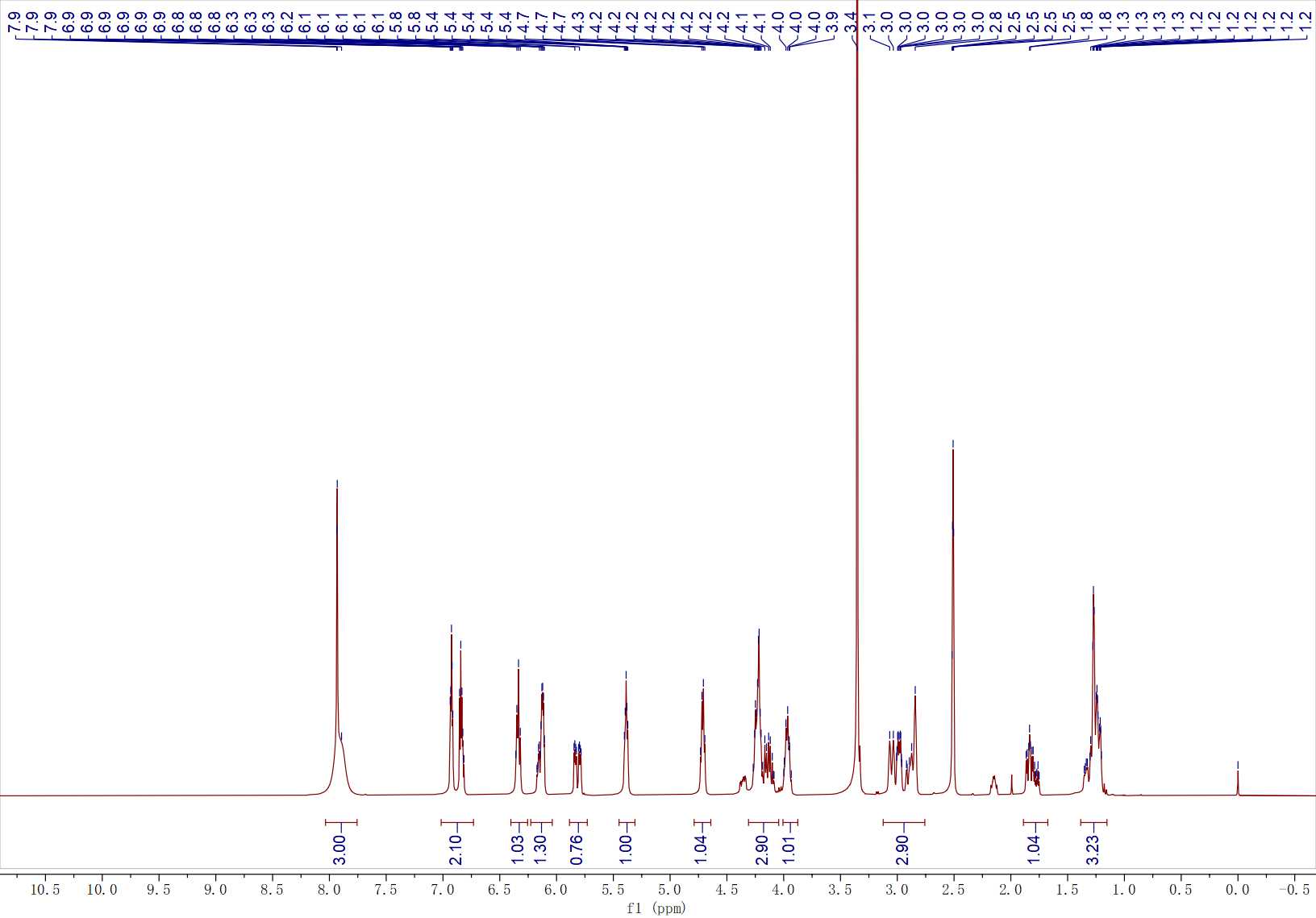


#### ^13^C NMR of 4e


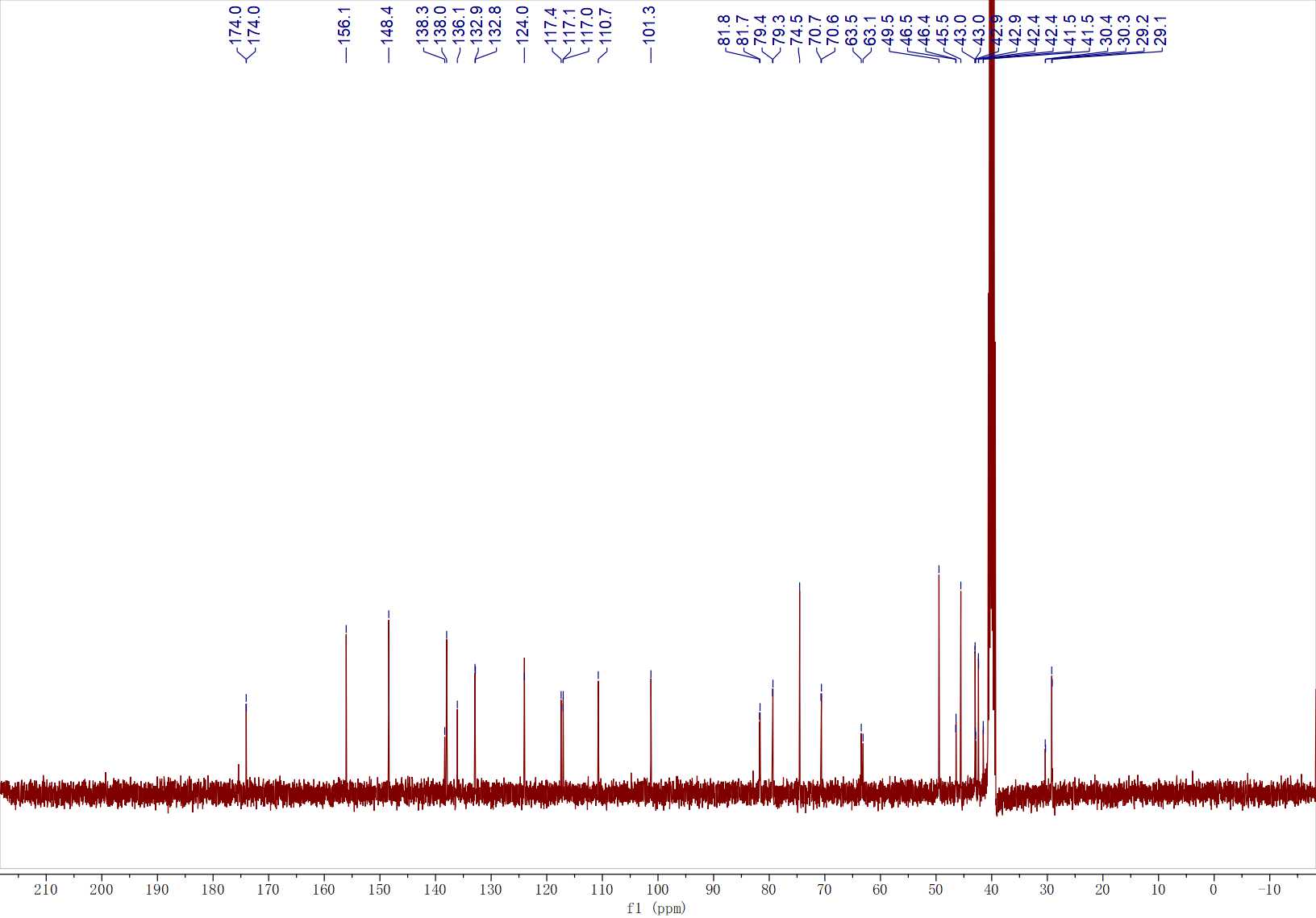


#### ESI-HRMS spectrum of 4e


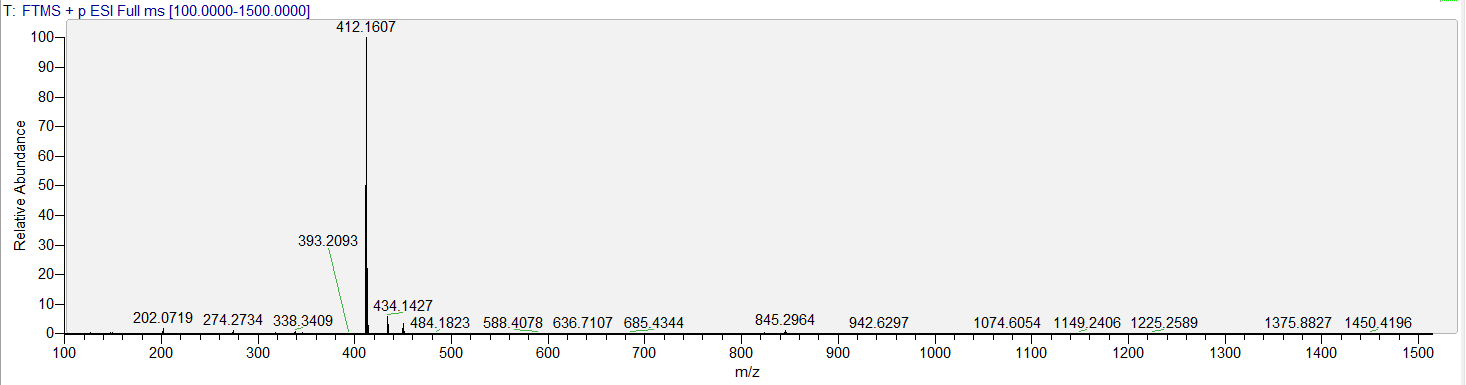


#### HPLC spectrum of 4e


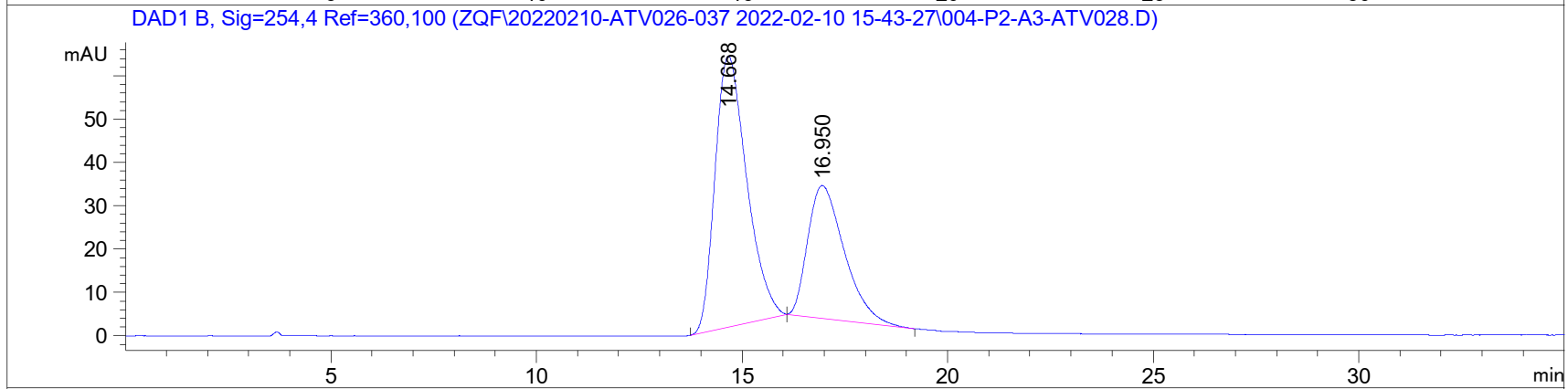


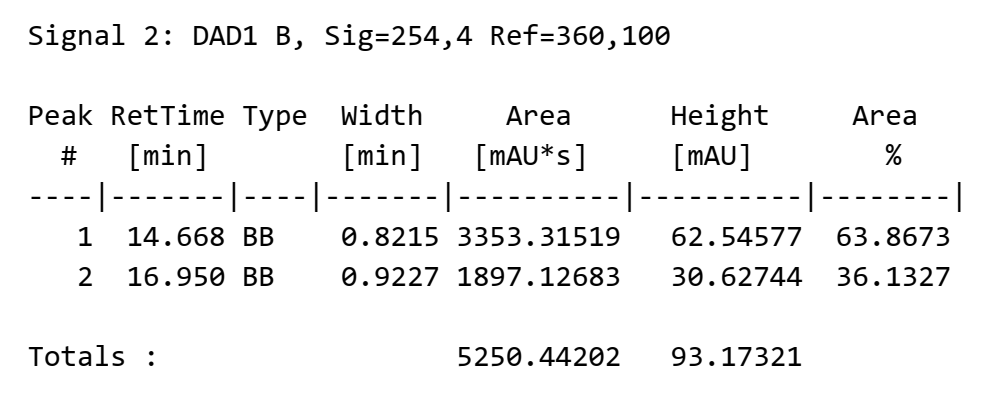


#### Spectrums of 4f

#### ^1^H NMR of 4f


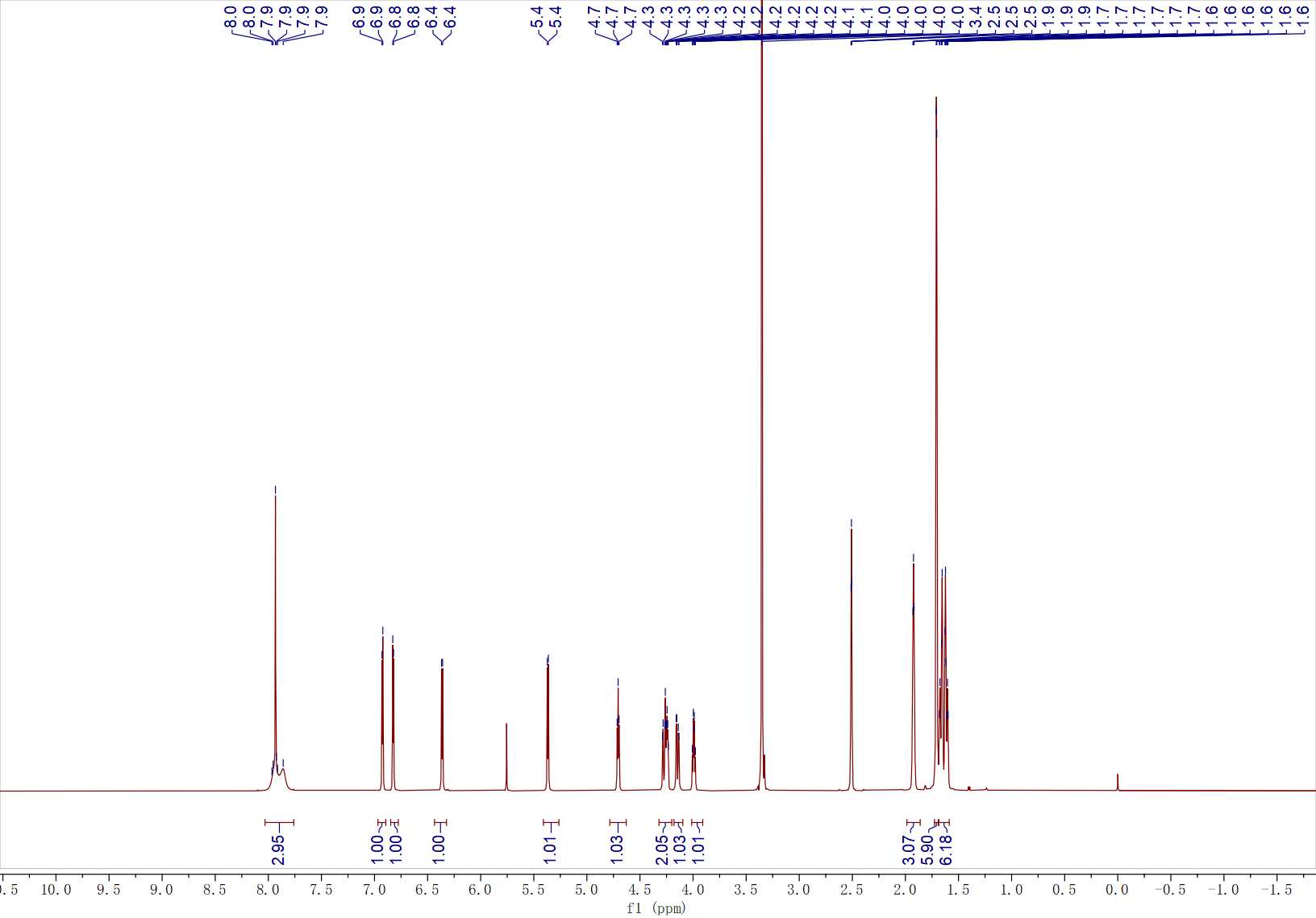


#### ^13^C NMR of 4f


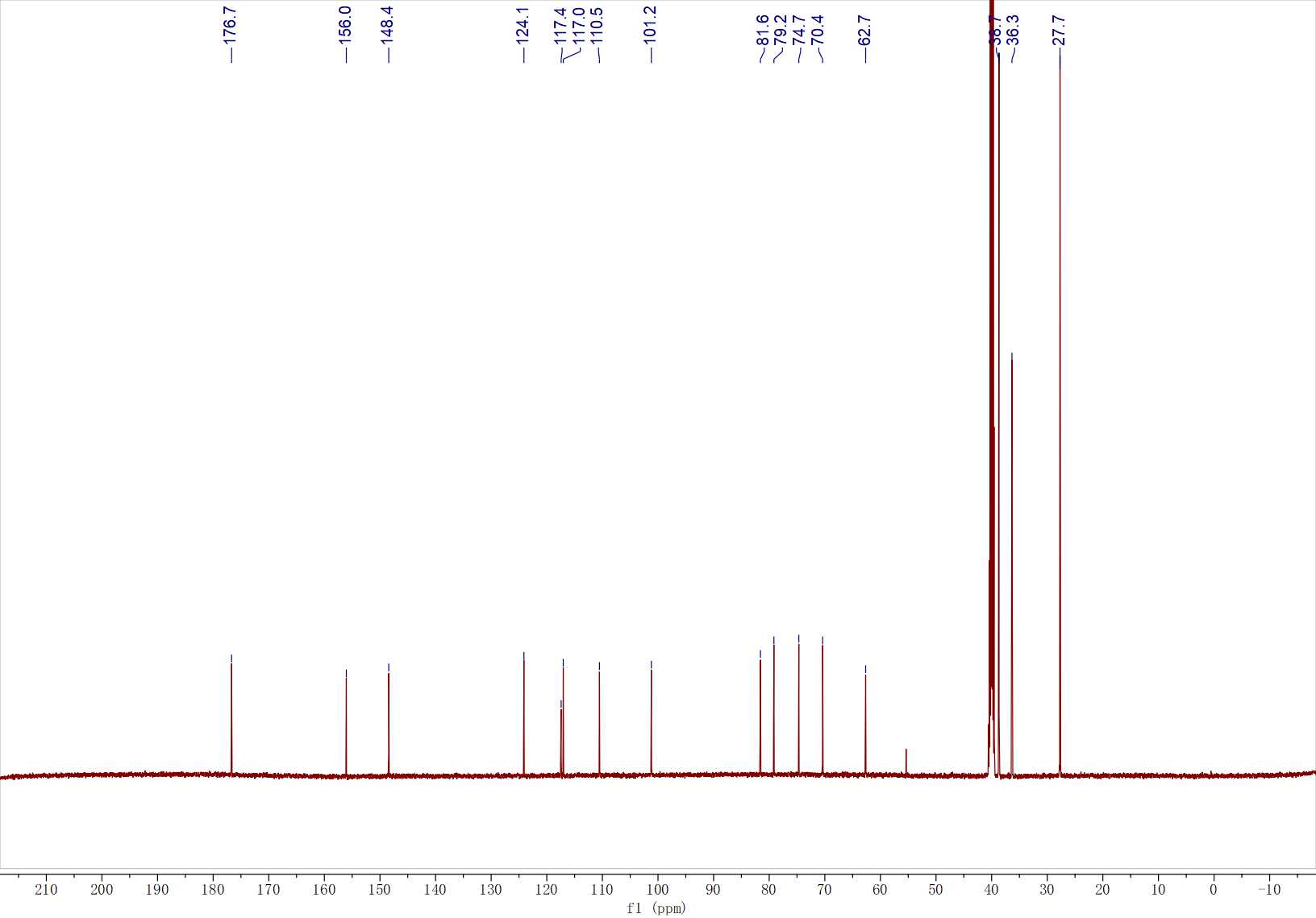


#### ESI-HRMS spectrum of 4f


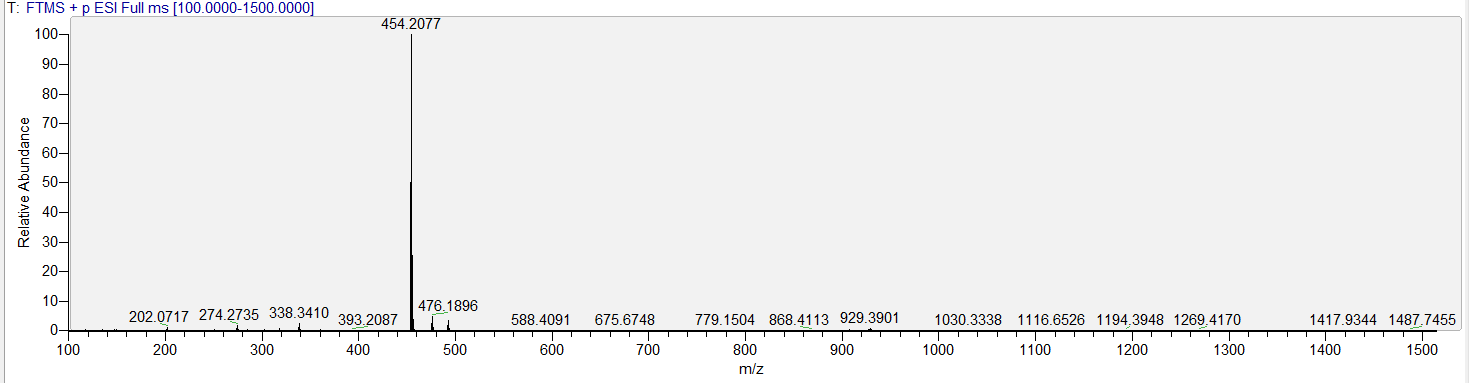


#### HPLC spectrum of 4f


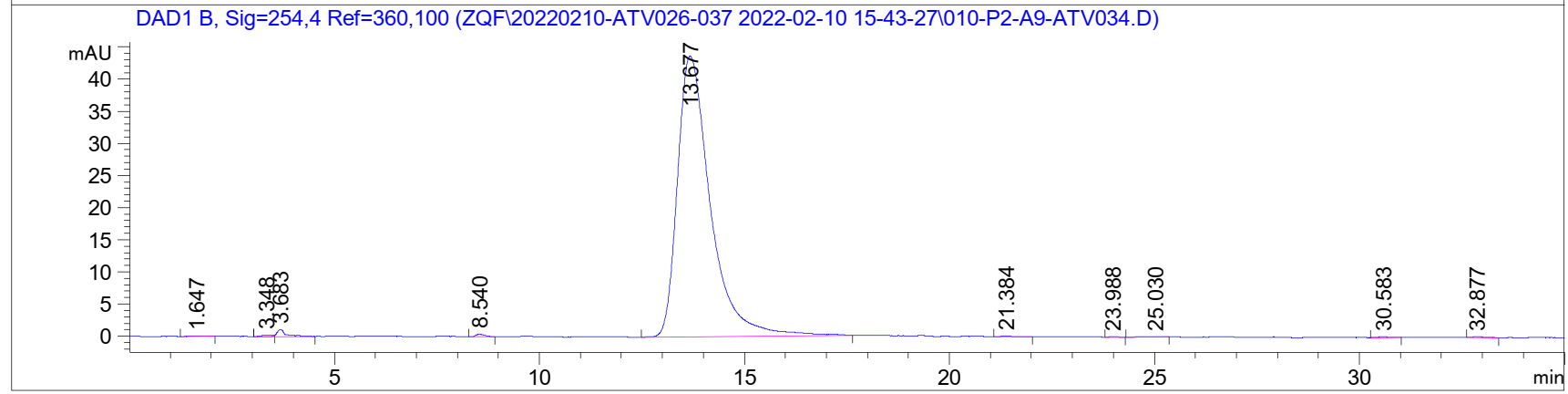


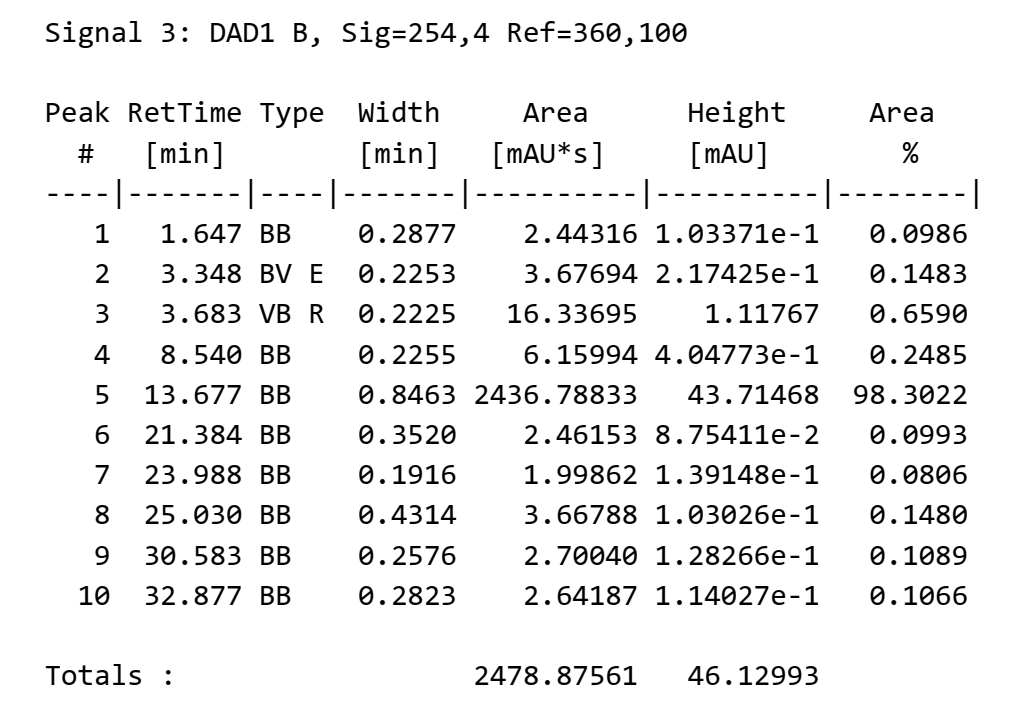


#### Spectrums of 4g

#### ^1^H NMR of 4g


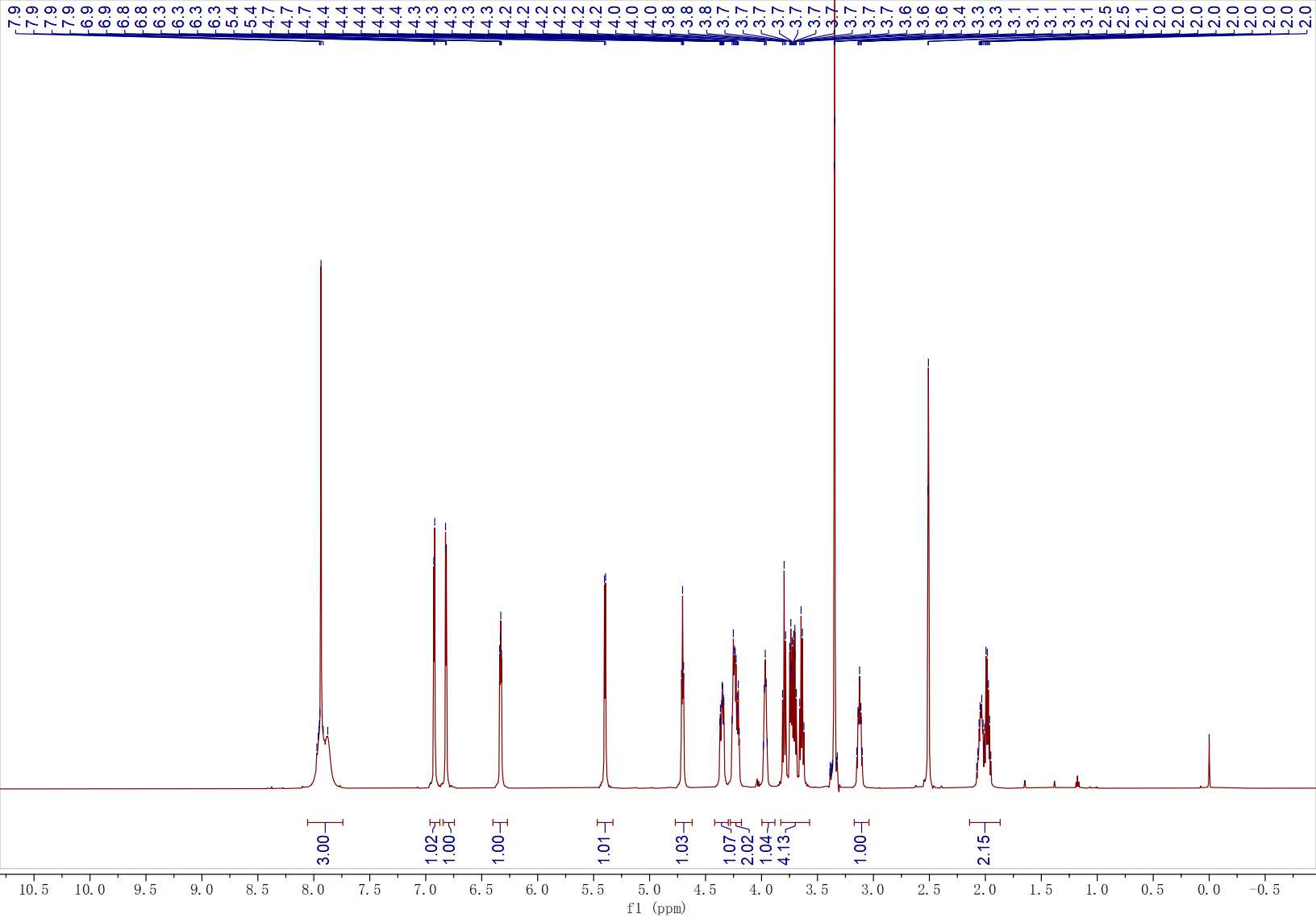


#### ^13^C NMR of 4g


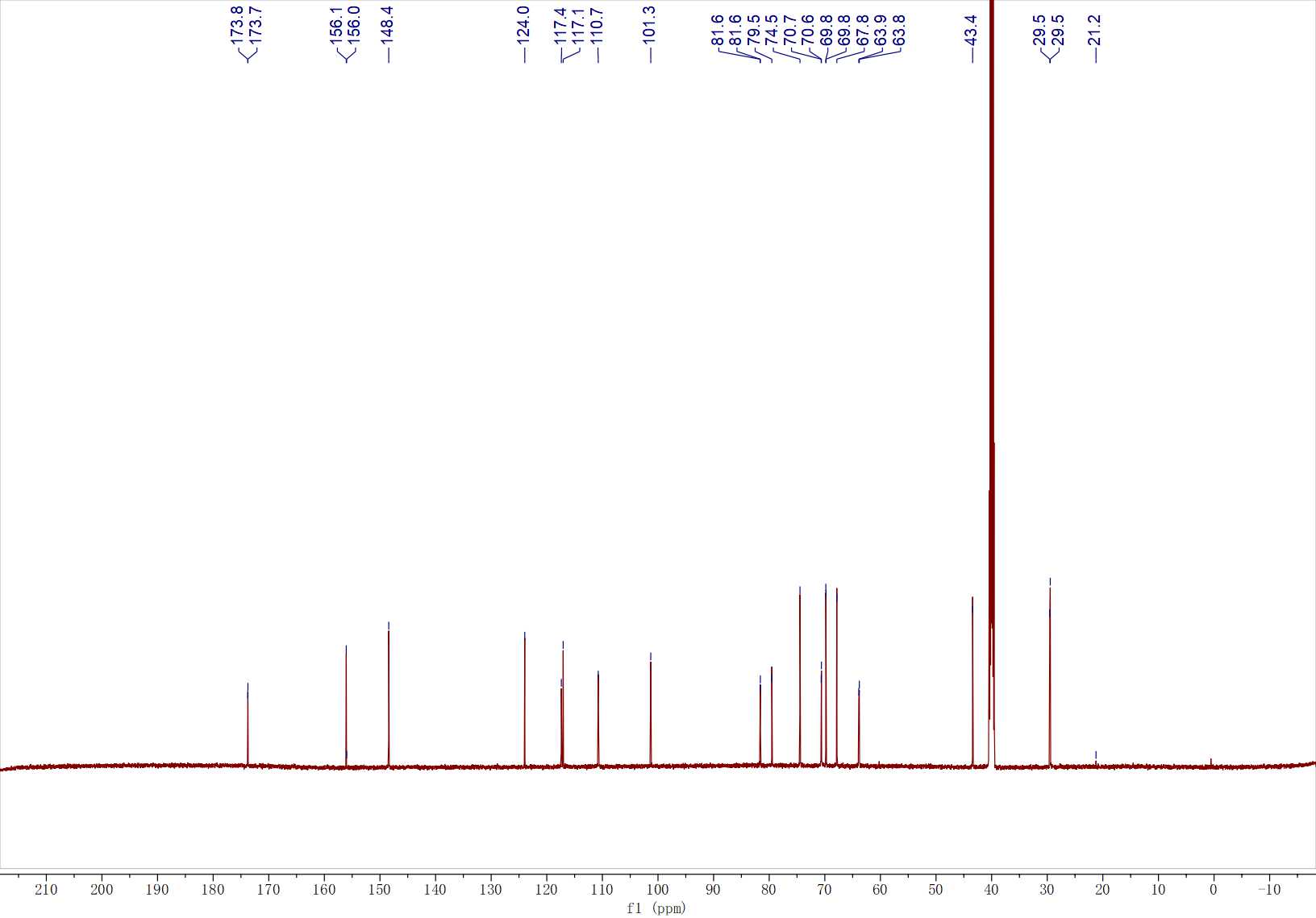


#### ESI-HRMS spectrum of 4g


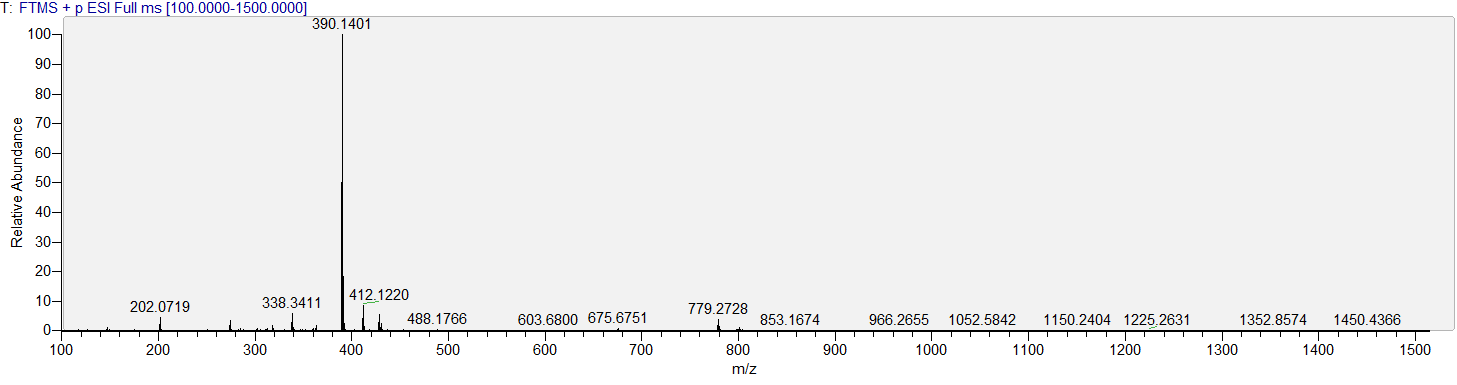


#### HPLC spectrum of 4g


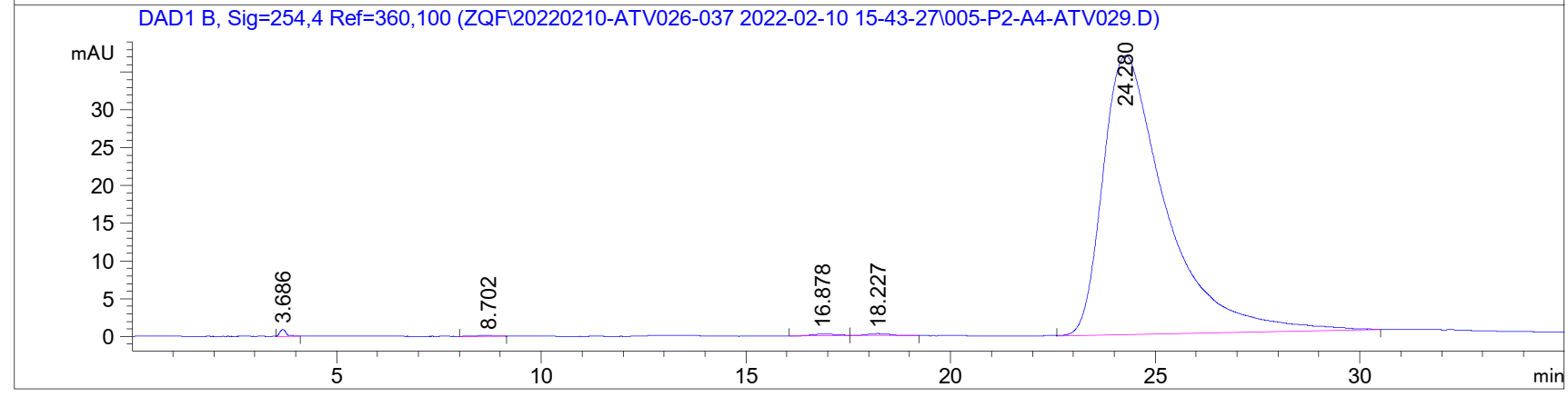


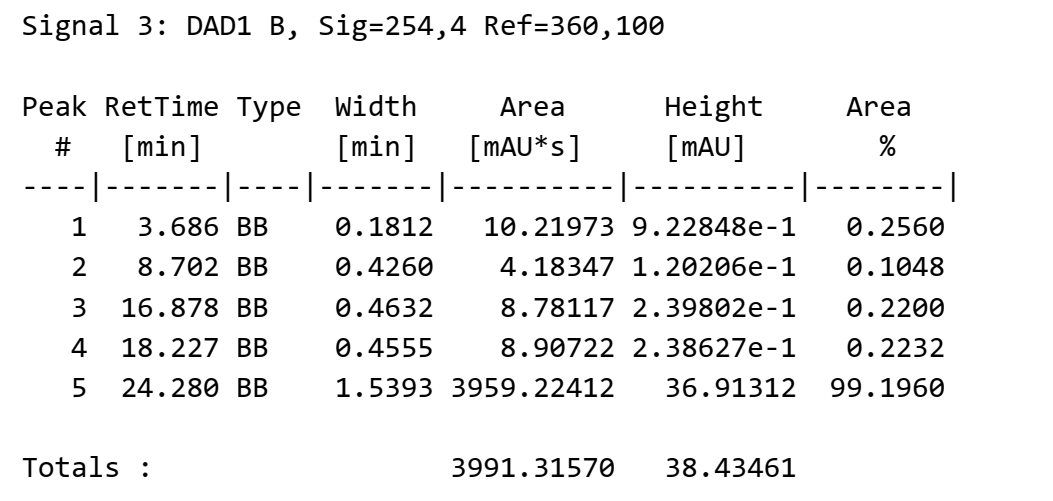


#### Spectrums of 4h

#### ^1^H NMR of 4h


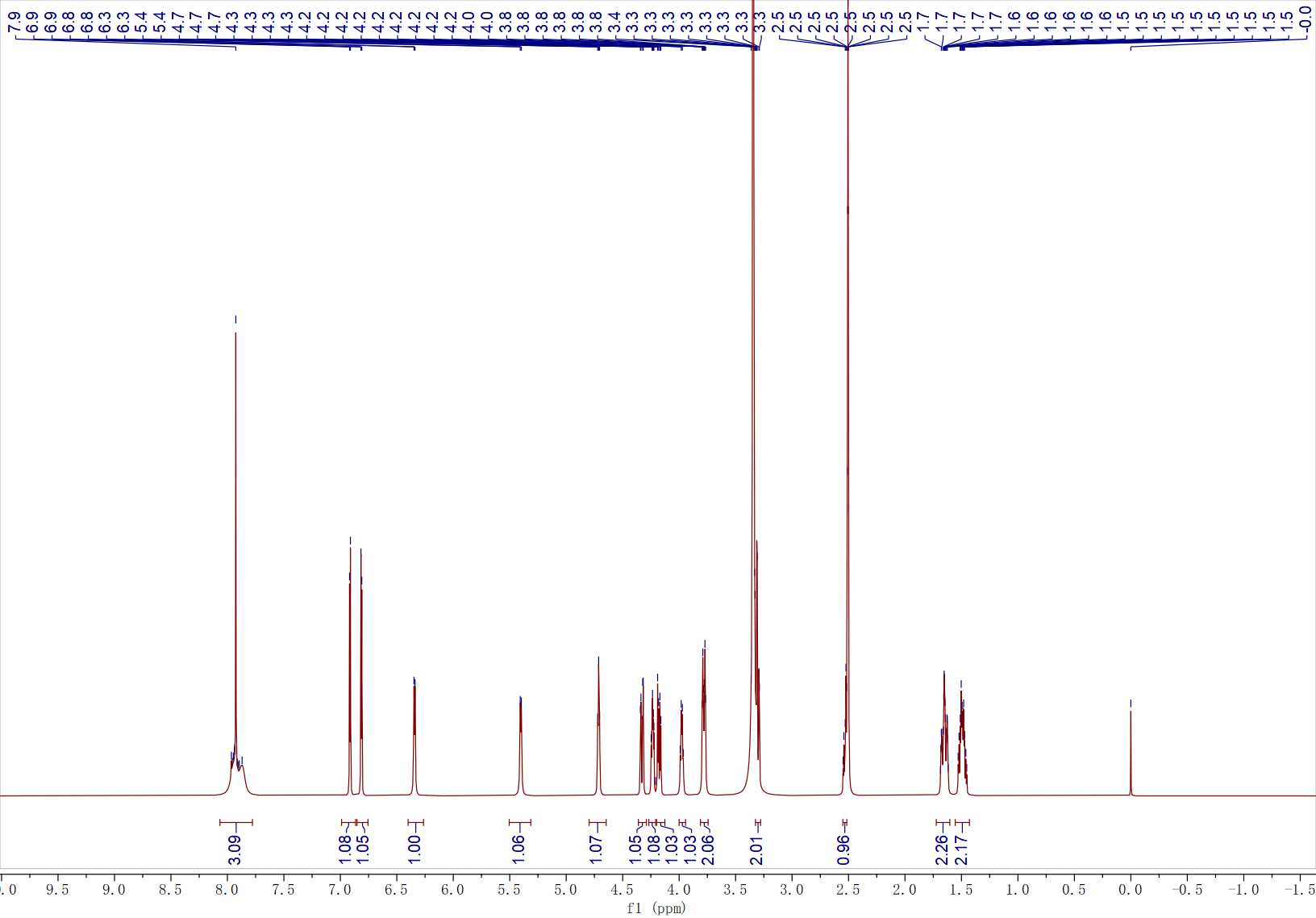


#### ^13^C NMR of 4h


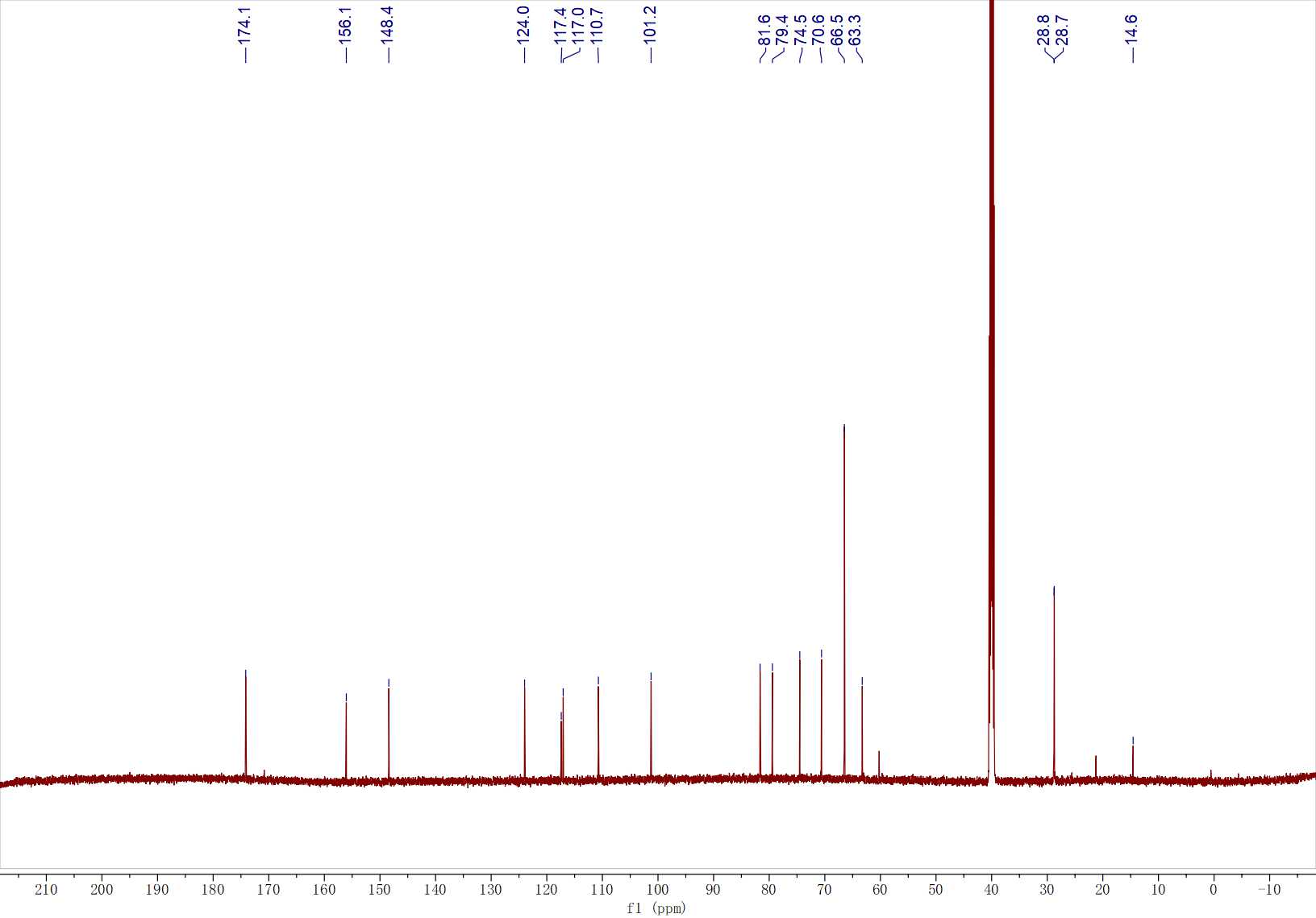


#### ESI-HRMS spectrum of 4h


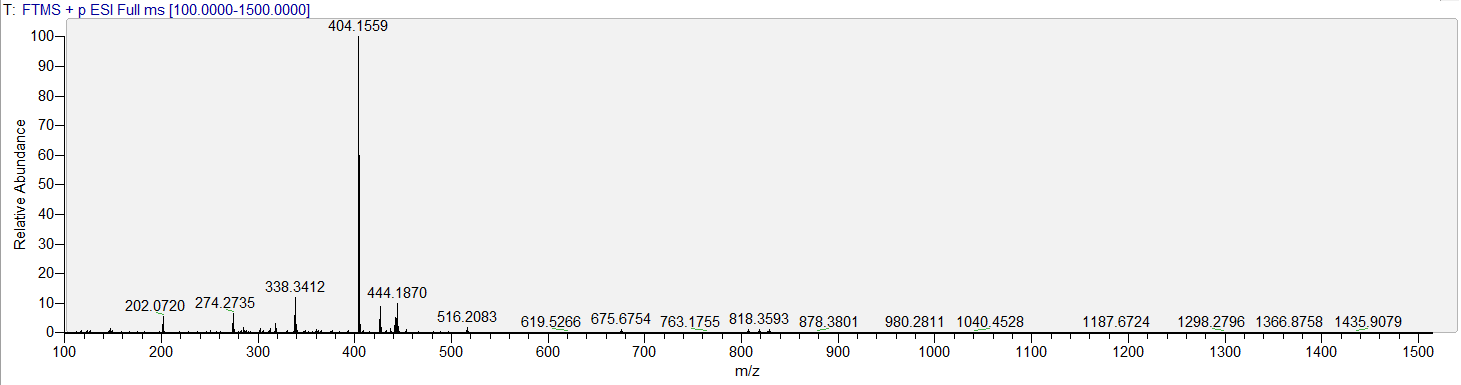


#### HPLC spectrum of 4h


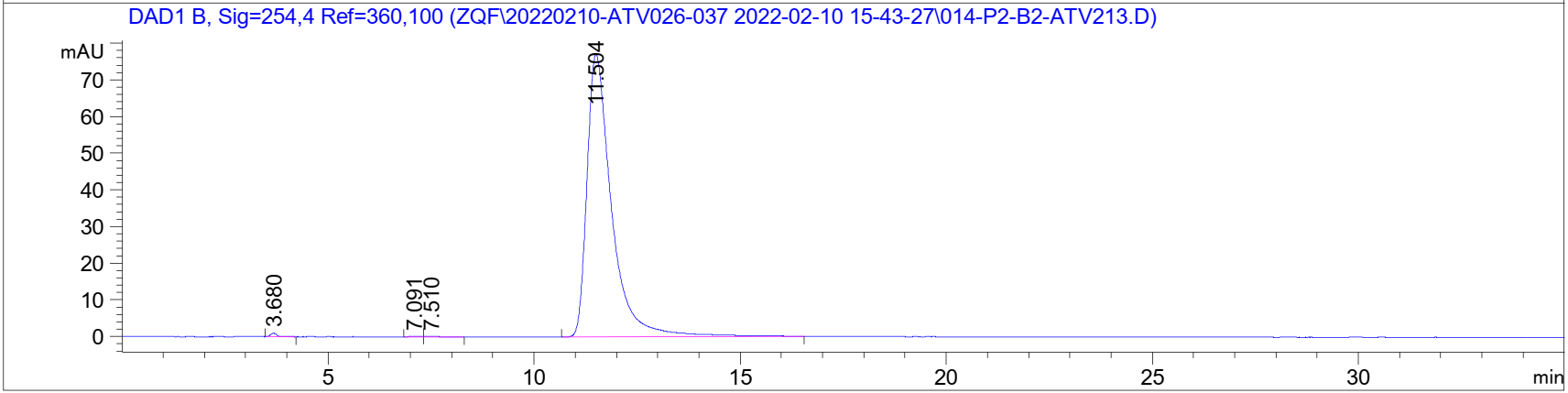


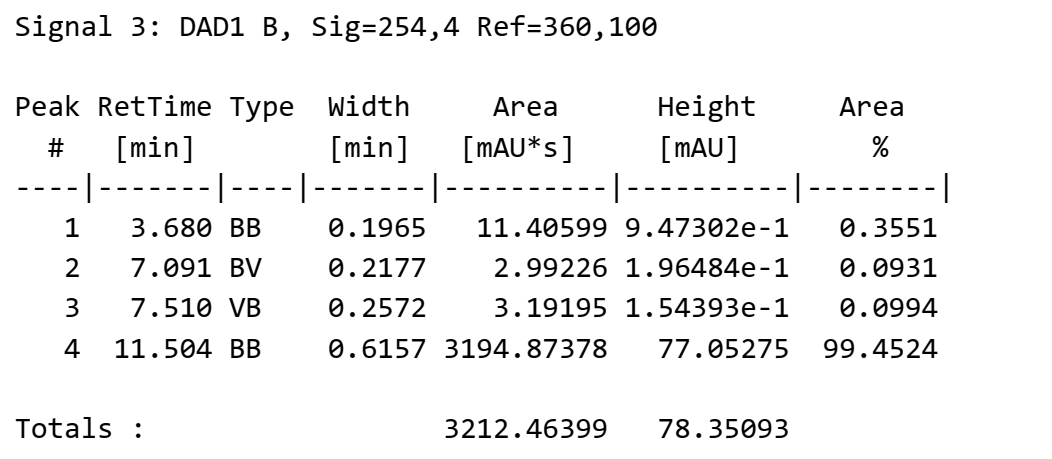


#### Spectrums of 4i

#### ^1^H NMR of 4i


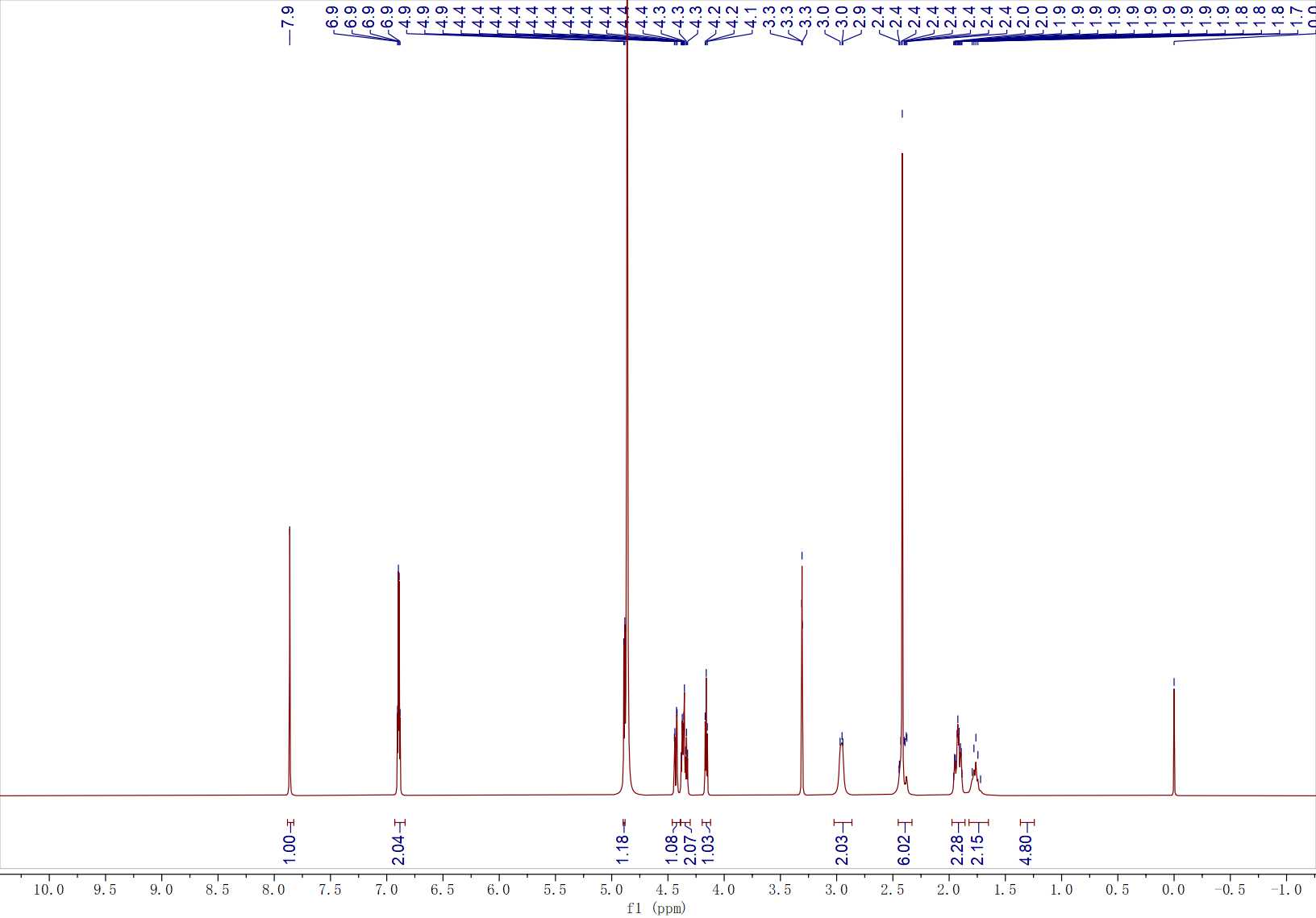


#### ^13^C NMR of 4i


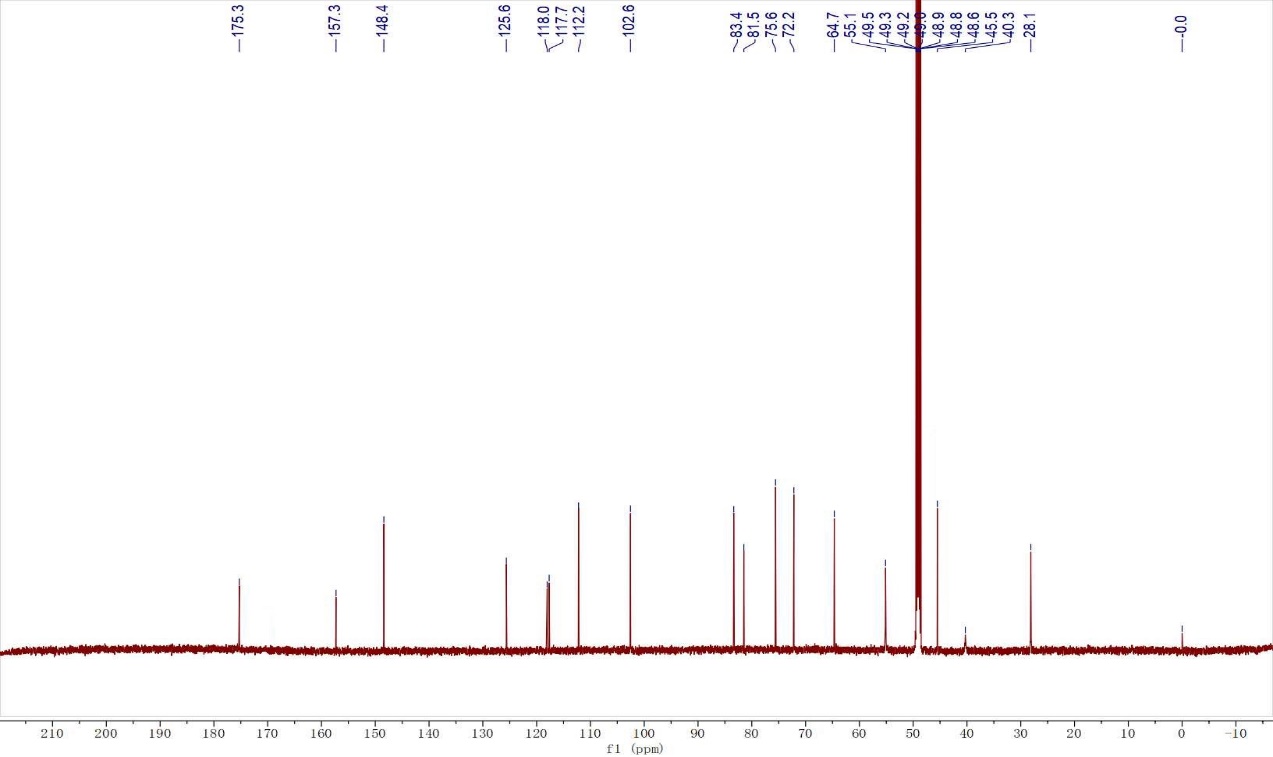


#### ESI-HRMS spectrum of 4i


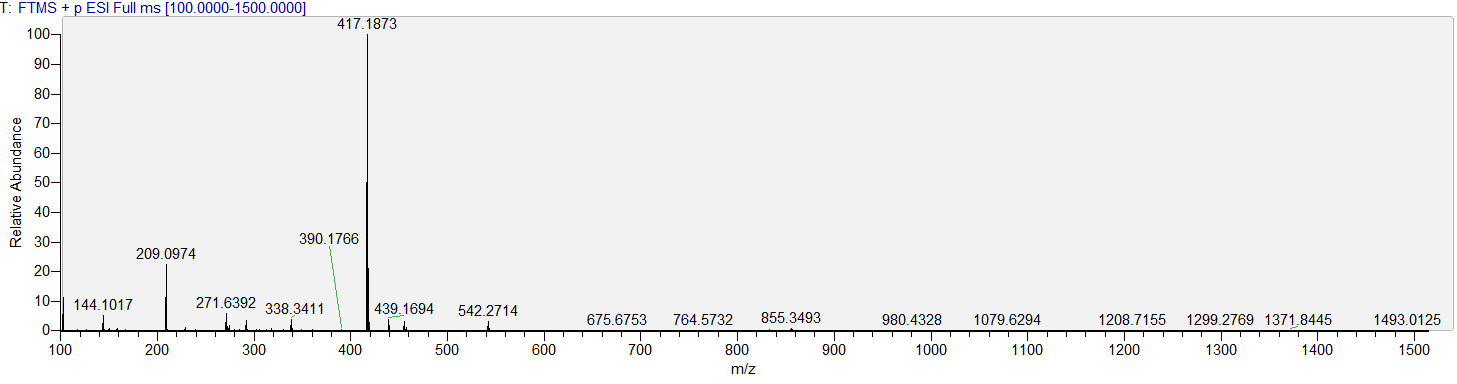


#### HPLC spectrum of 4i


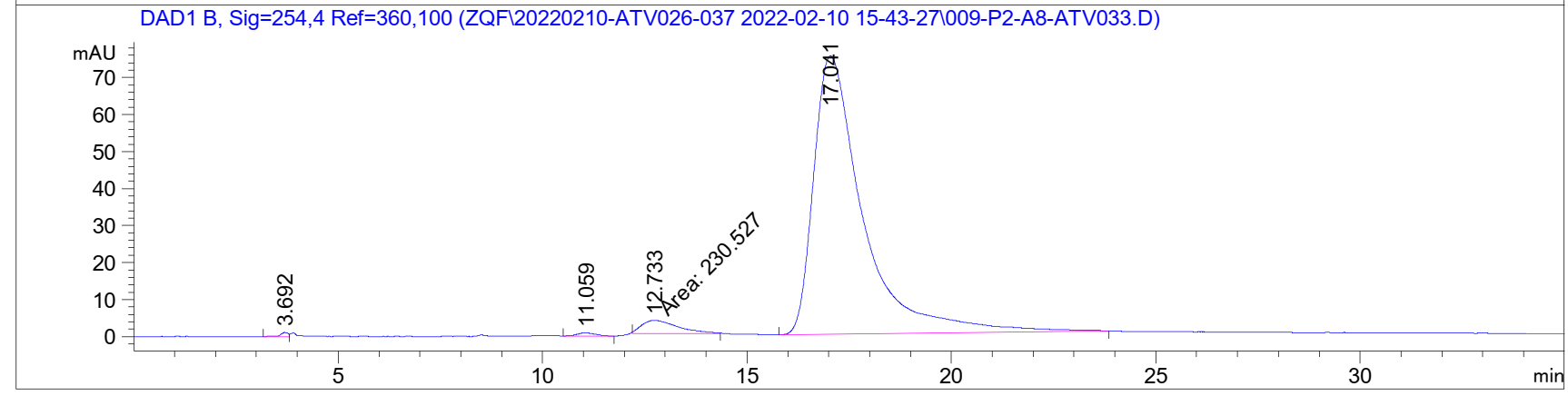


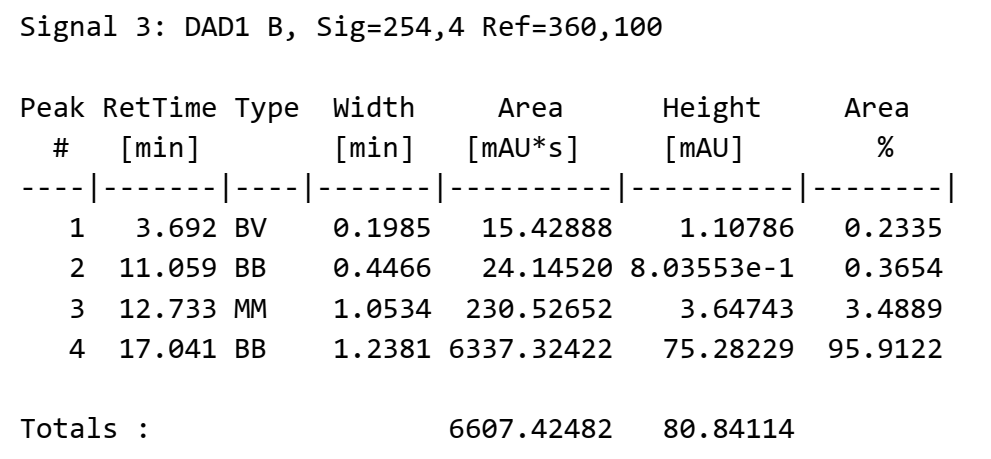


#### Spectrums of 4j

#### ^1^H NMR of 4j


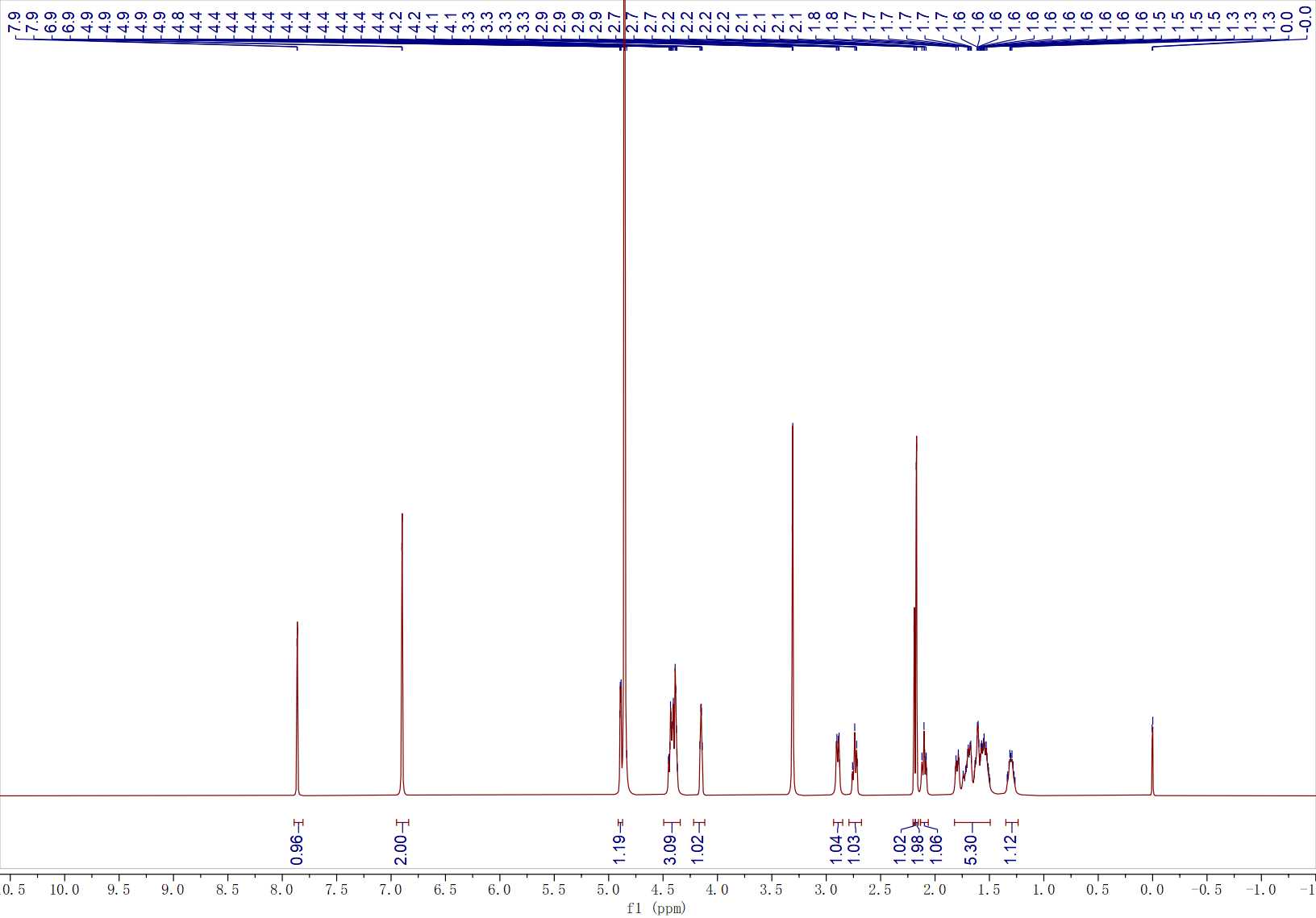


#### ^13^C NMR of 4j


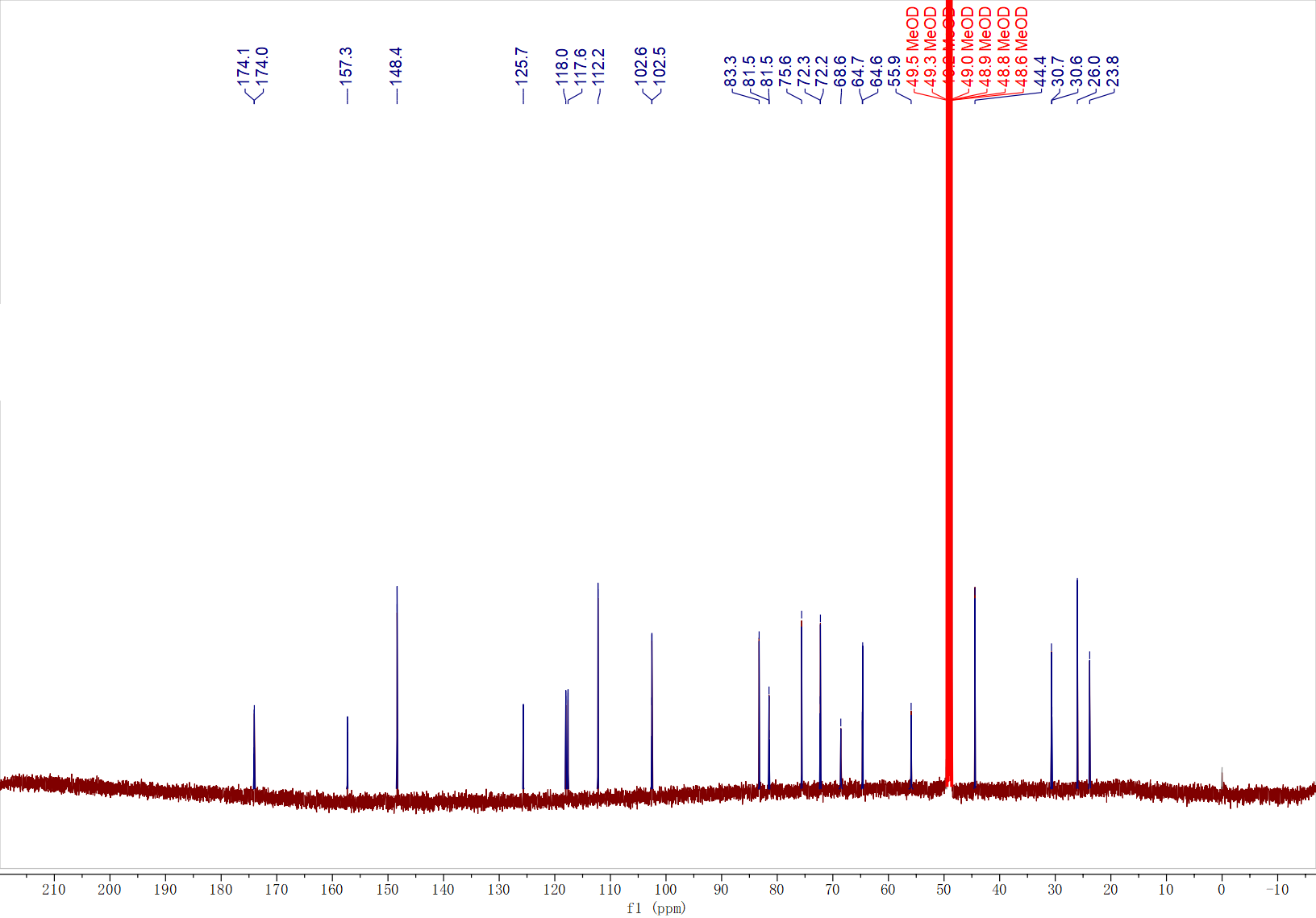


#### ESI-HRMS spectrum of 4j


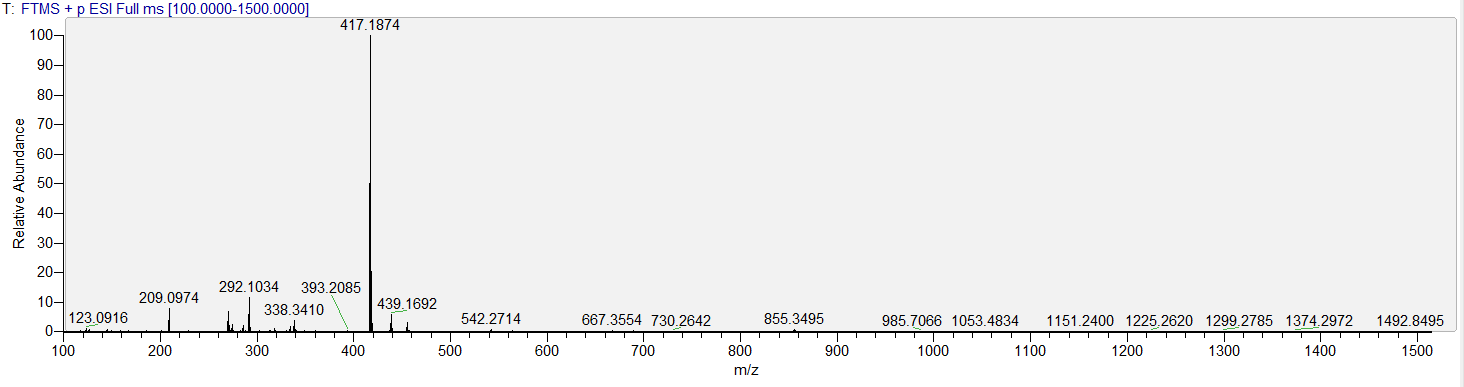


#### HPLC spectrum of 4j


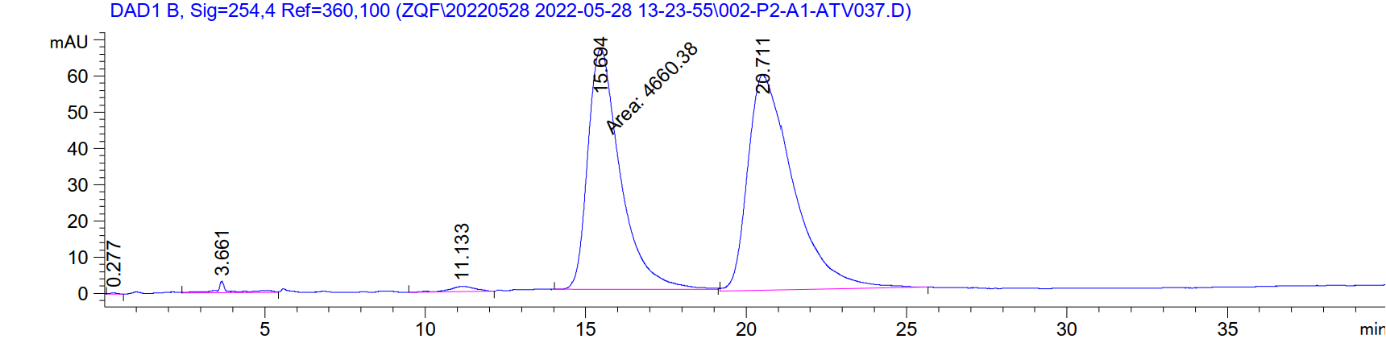


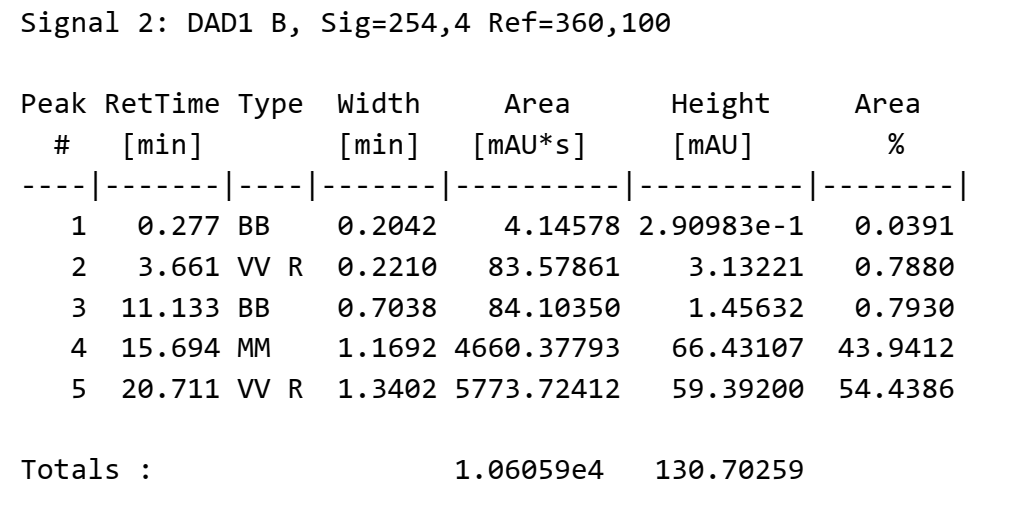


#### Spectrums of 4k

#### ^1^H NMR of 4k


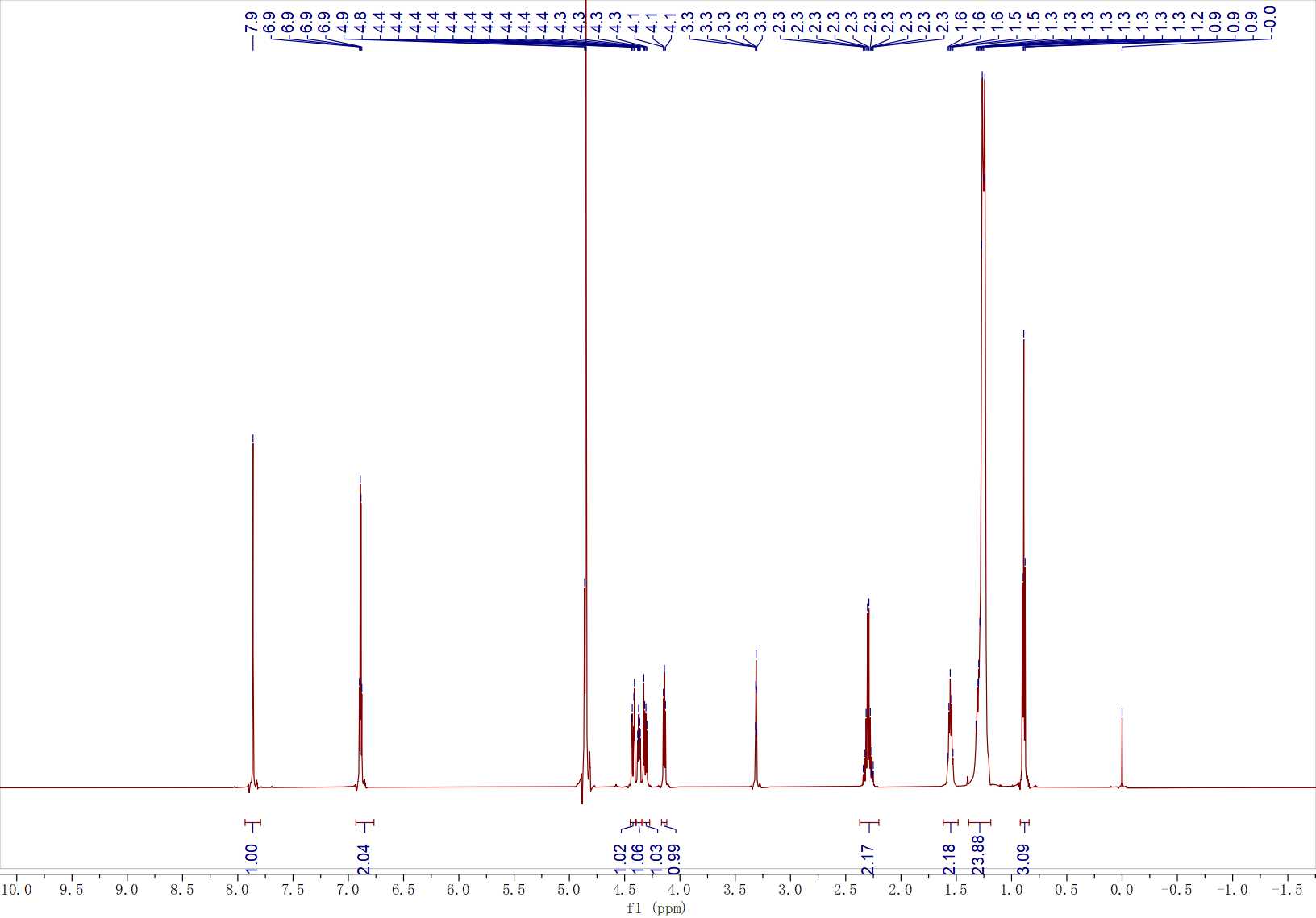


#### ^13^C NMR of 4k


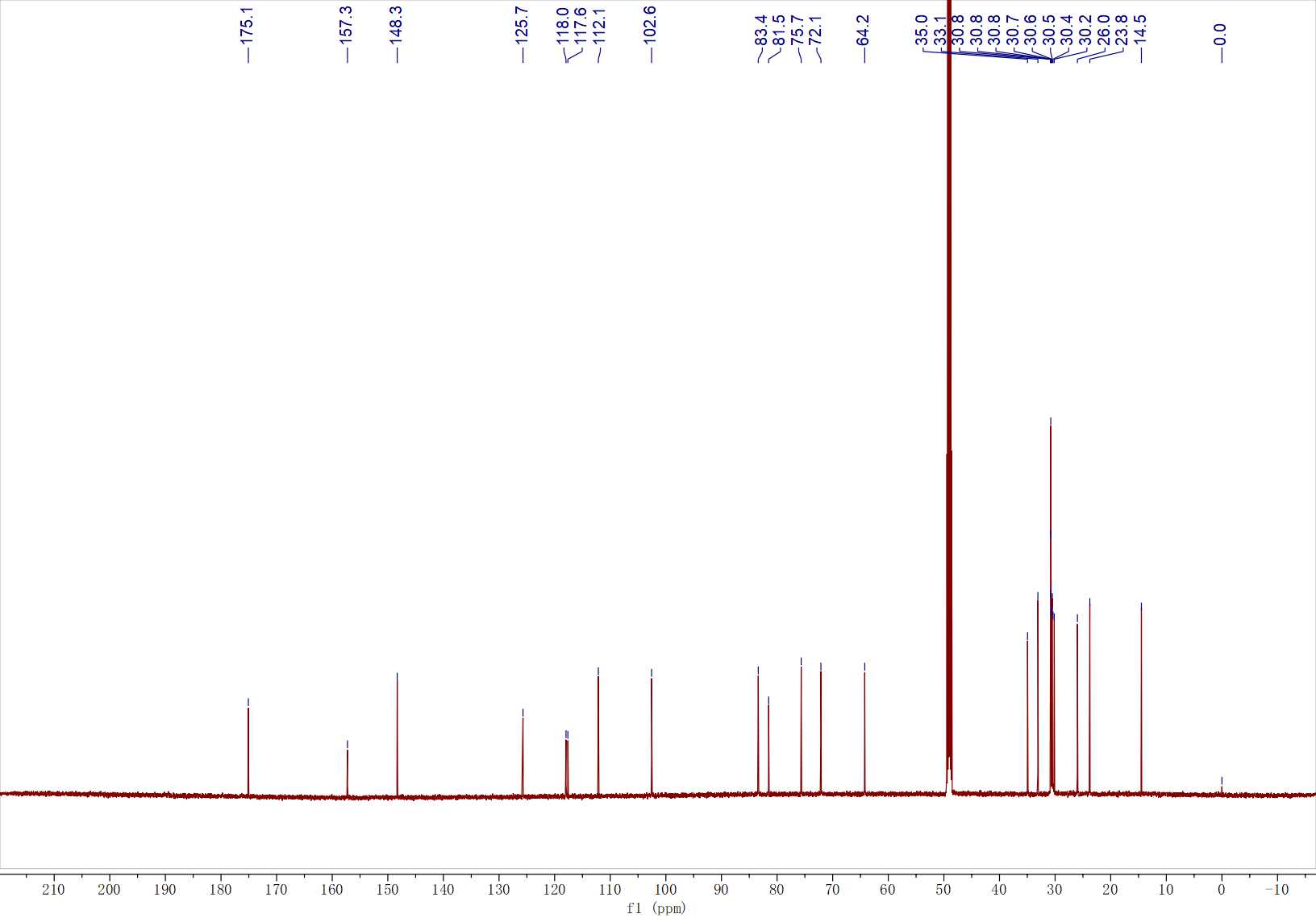


#### ESI-HRMS spectrum of 4k


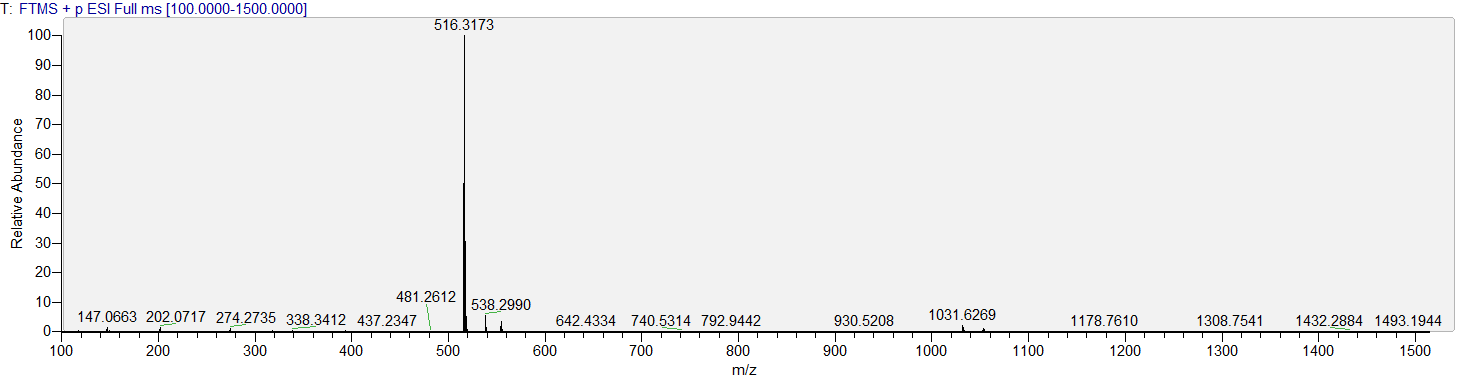


#### HPLC spectrum of 4k


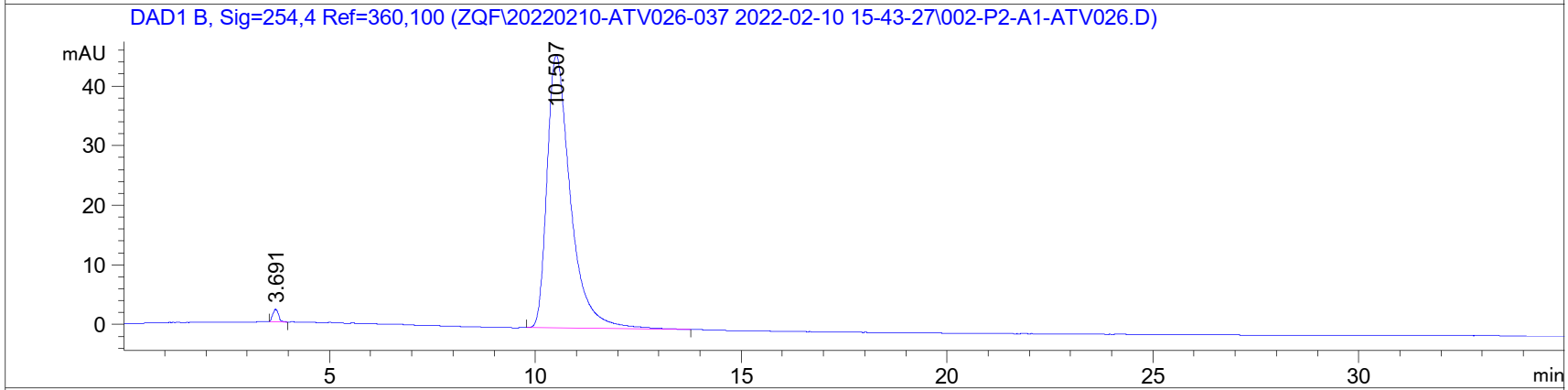


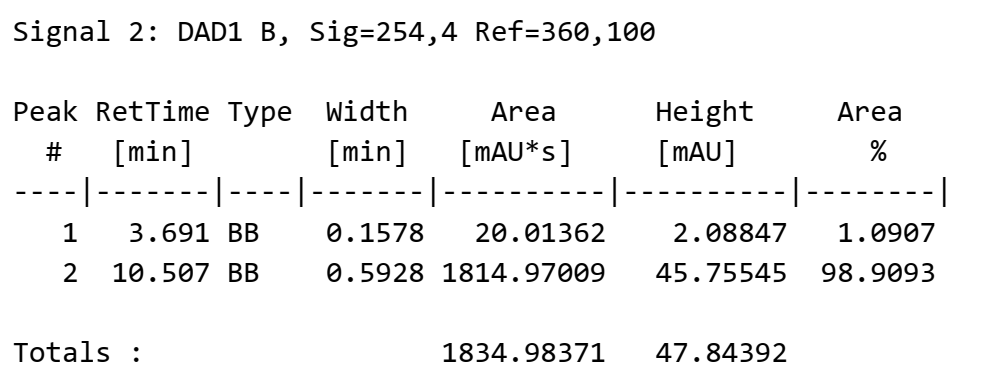


#### Spectrums of 4l

#### ^1^H NMR of 4l


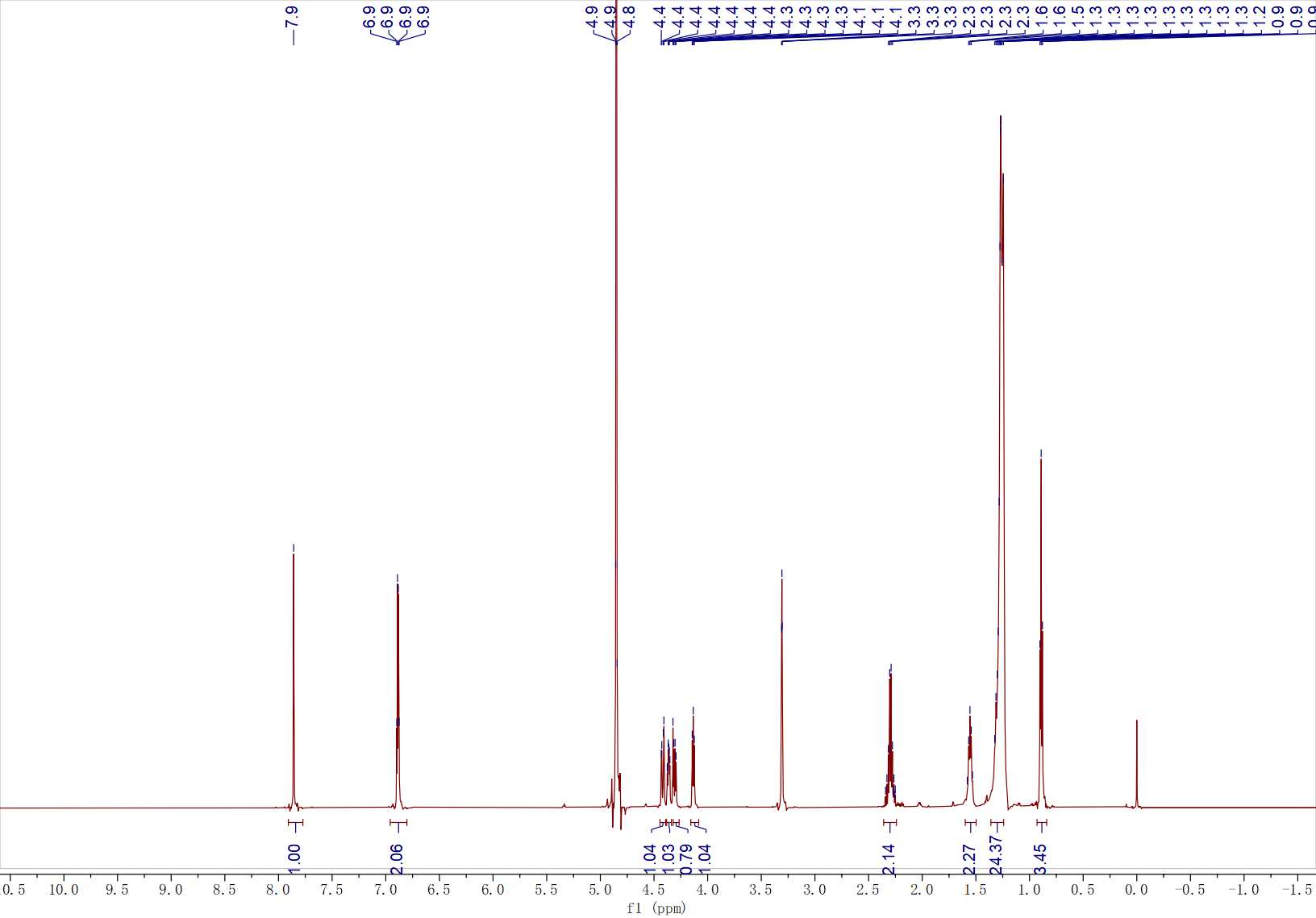


#### ^13^C NMR of 4l


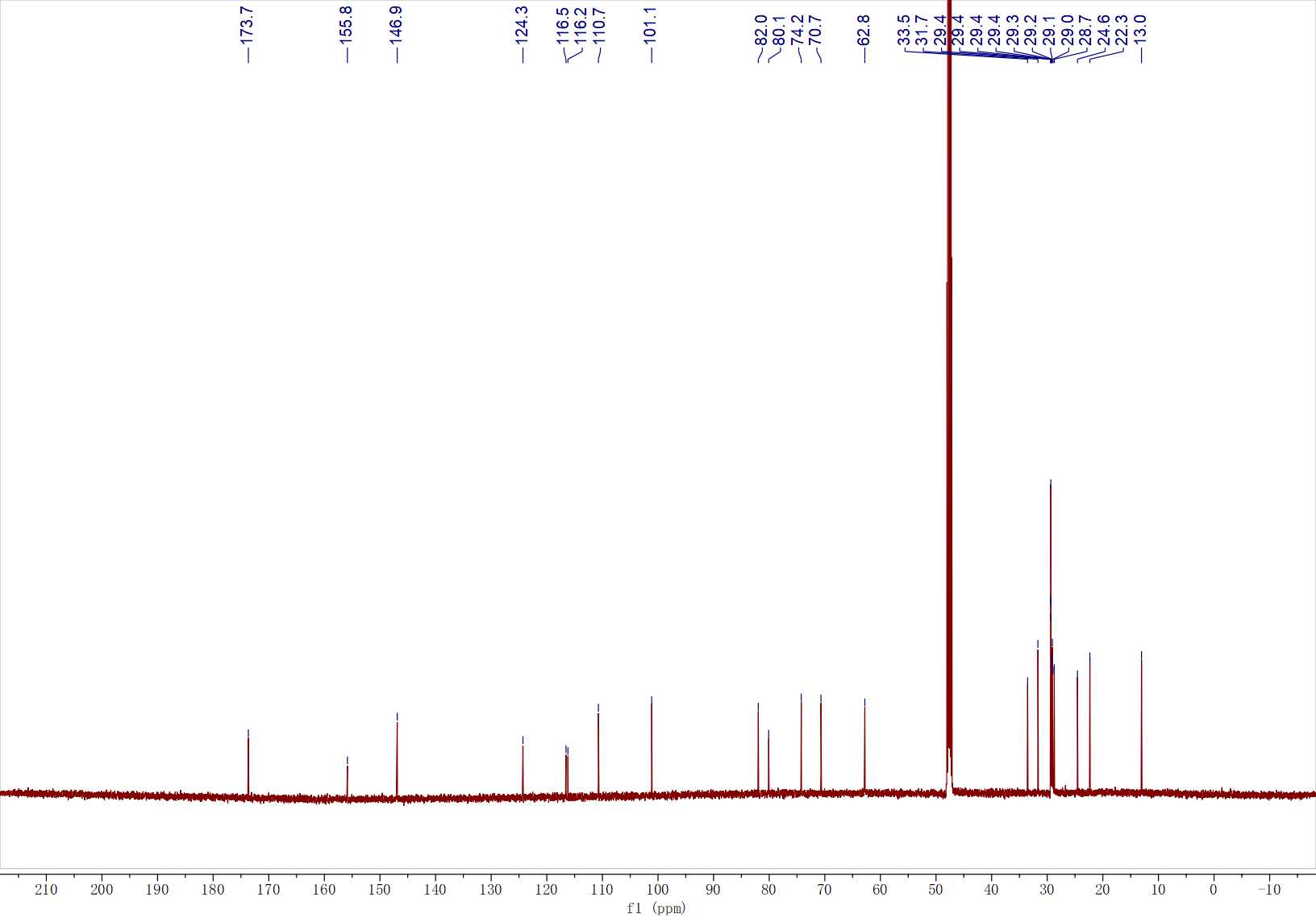


#### ESI-HRMS spectrum of 4l


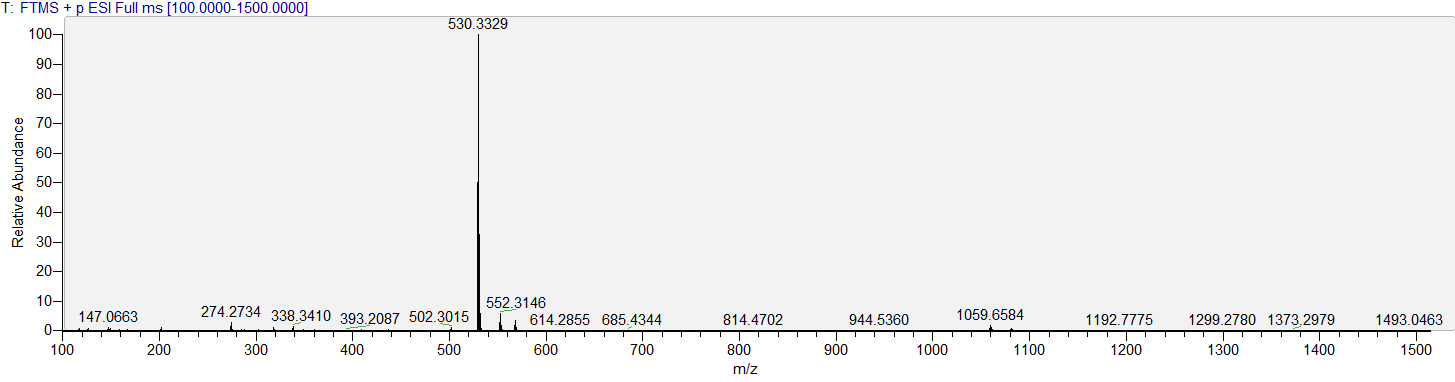


#### HPLC spectrum of 4l


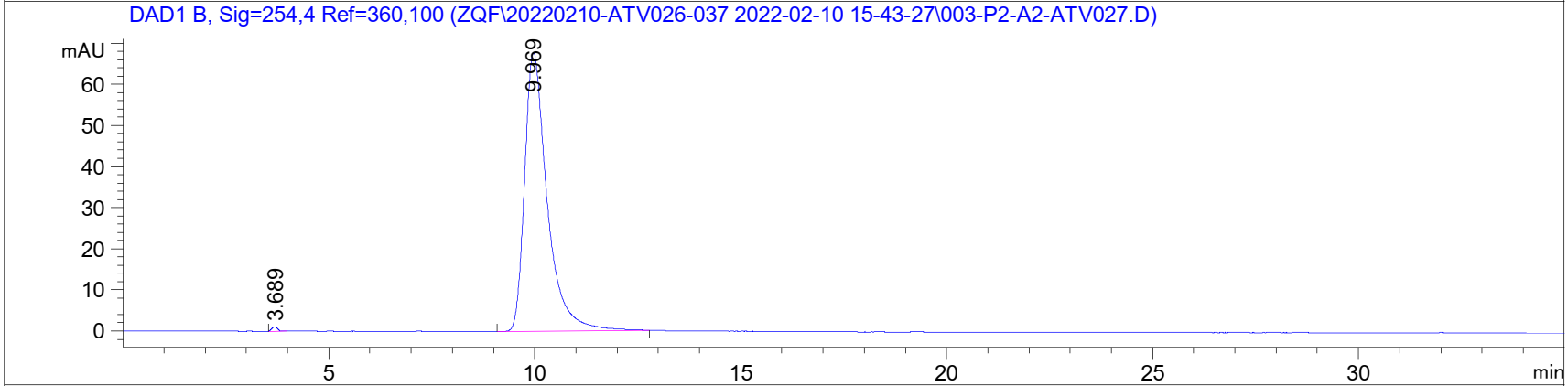


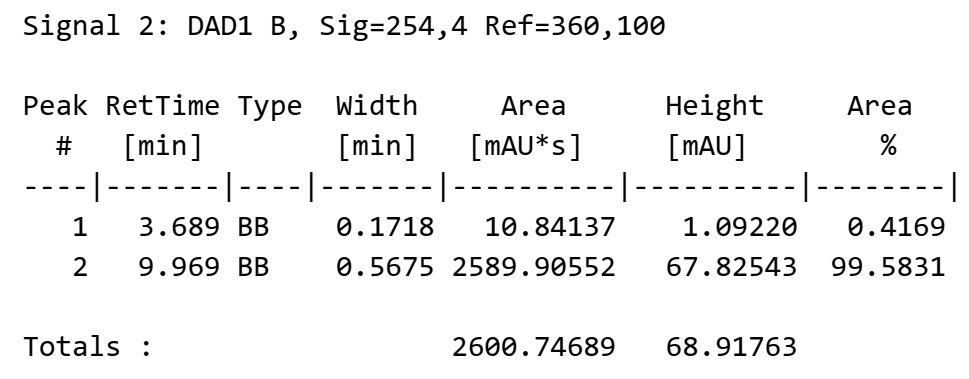


#### Spectrums of 4m

#### ^1^H NMR of 4m


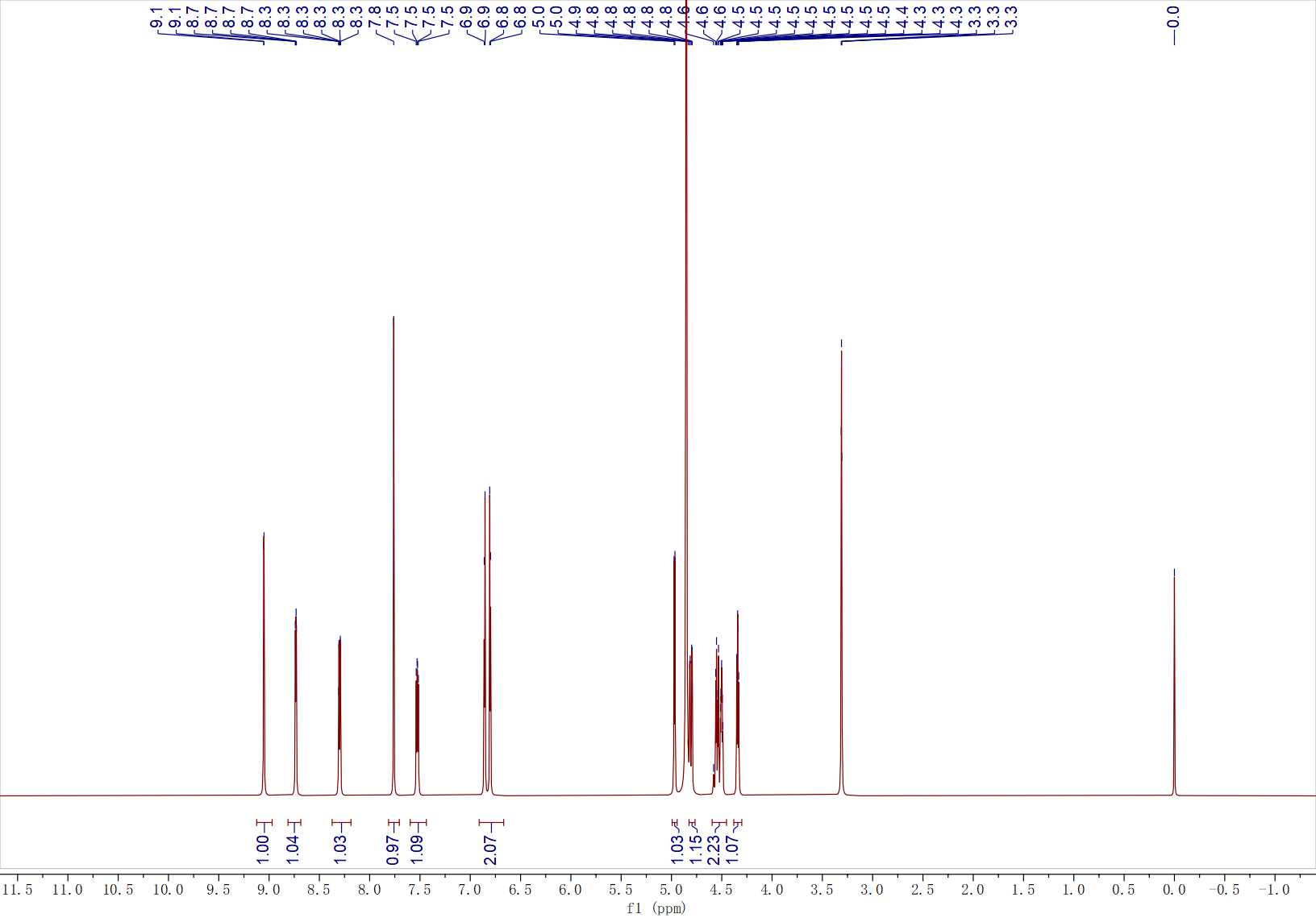


#### ^13^C NMR of 4m


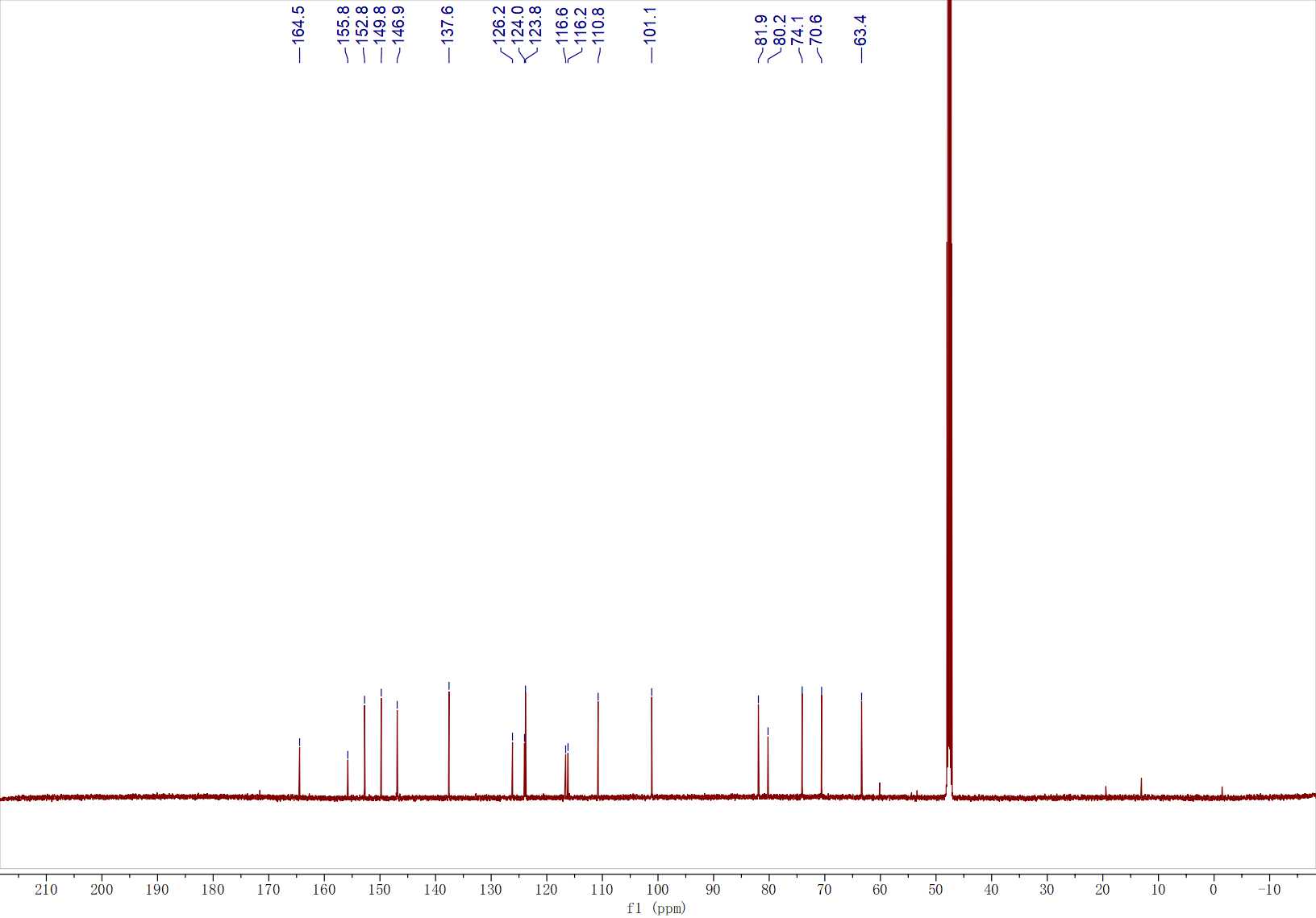


#### ESI-HRMS spectrum of 4m


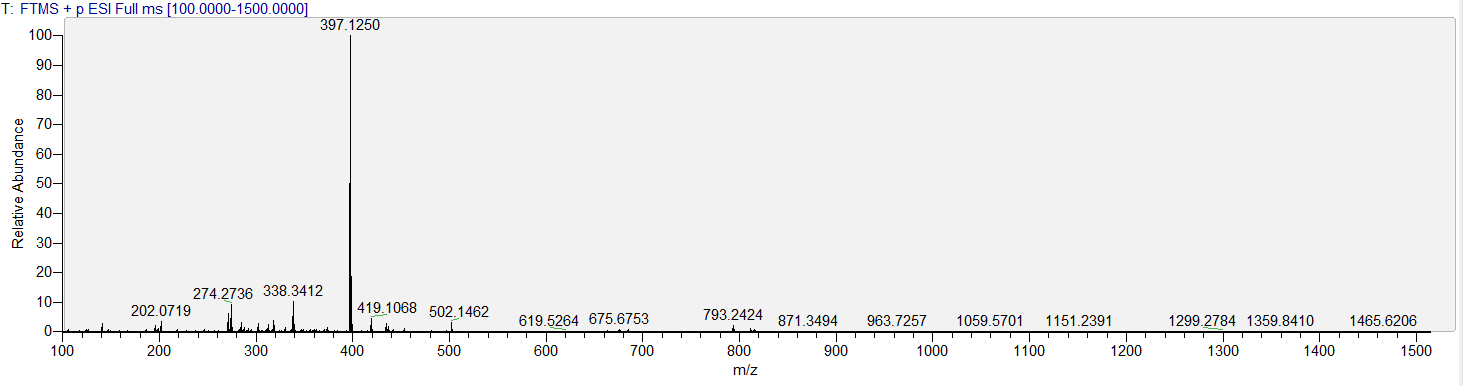


#### HPLC spectrum of 4m


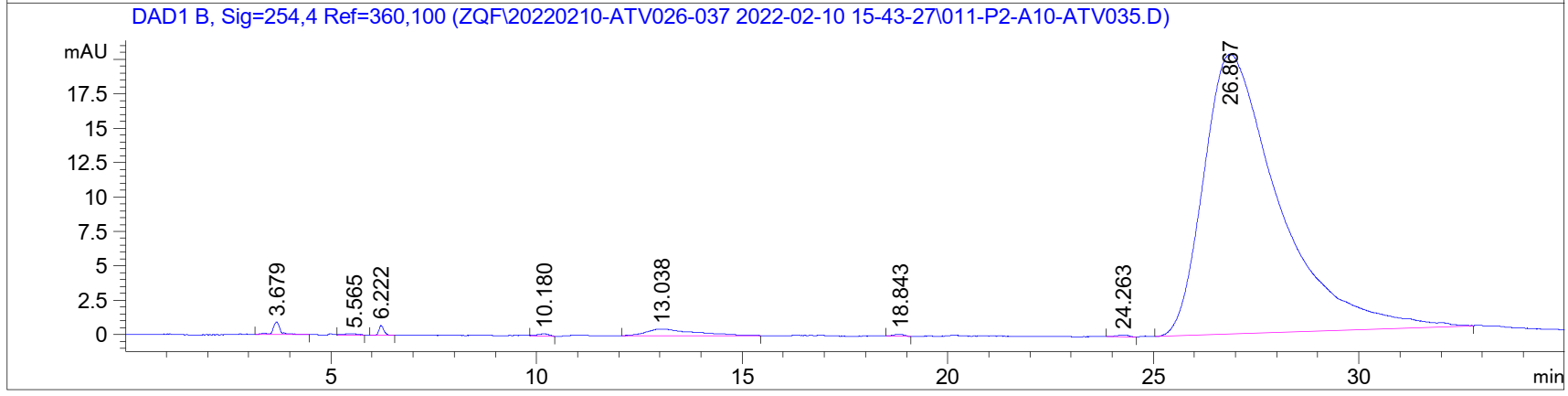


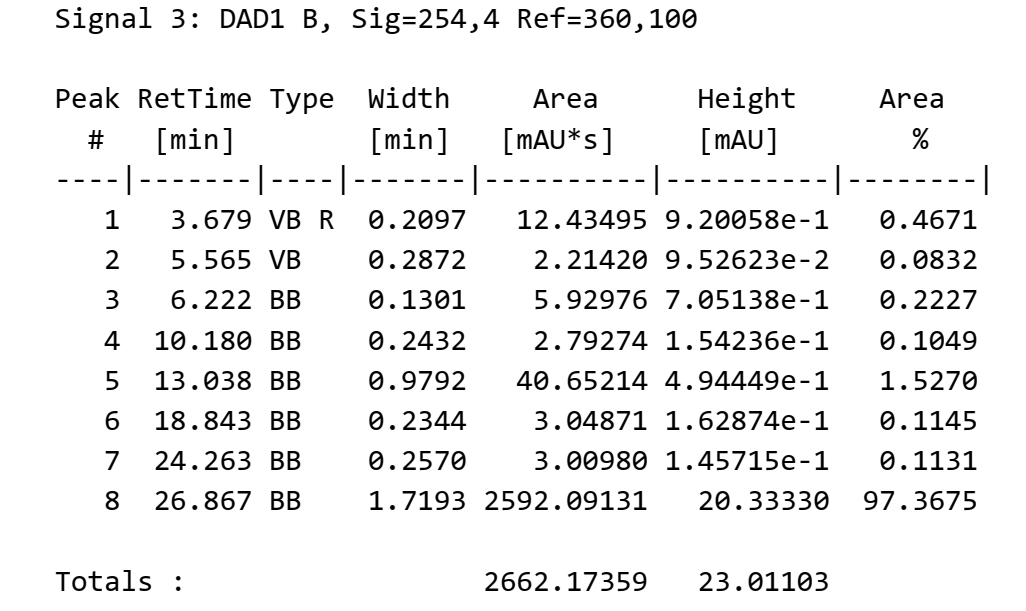

Supplement: Supplementary file 1 — Preclinical characterization and anti-SARS-CoV-2 efficacy of ATV014: Oral cyclohexanecarboxylate prodrug of 1′-CN-4-aza-7,9-dideazaadenosine C-nucleoside [file 41392_2023_1310_MOESM1_ESM.docx]
